# Demonstration of Protein-Based Human Identification Using the Hair Shaft Proteome

**DOI:** 10.1371/journal.pone.0160653

**Published:** 2016-09-07

**Authors:** Glendon J. Parker, Tami Leppert, Deon S. Anex, Jonathan K. Hilmer, Nori Matsunami, Lisa Baird, Jeffery Stevens, Krishna Parsawar, Blythe P. Durbin-Johnson, David M. Rocke, Chad Nelson, Daniel J. Fairbanks, Andrew S. Wilson, Robert H. Rice, Scott R. Woodward, Brian Bothner, Bradley R. Hart, Mark Leppert

**Affiliations:** 1 Department of Biology, Utah Valley University, Orem, Utah, United States of America; 2 Protein-Based Identification Technologies L.L.C., Orem, Utah, United States of America; 3 Department of Human Genetics, University of Utah, Salt Lake City, Utah, United States of America; 4 Forensic Science Center, Lawrence Livermore National Laboratory, Livermore, California, United States of America; 5 Department of Chemistry and Biochemistry, Montana State University, Bozeman, Montana, United States of America; 6 Mass Spectrometry and Proteomics Core Facility, University of Utah, Salt Lake City, Utah, United States of America; 7 Department of Public Health Sciences, University of California, Davis, California, United States of America; 8 School of Archaeological Sciences, University of Bradford, Bradford, United Kingdom; 9 Department of Environmental Toxicology, University of California, Davis, California, United States of America; 10 Sorenson Molecular Genealogical Foundation, Salt Lake City, Utah, United States of America; Universitat Pompeu Fabra, SPAIN

## Abstract

Human identification from biological material is largely dependent on the ability to characterize genetic polymorphisms in DNA. Unfortunately, DNA can degrade in the environment, sometimes below the level at which it can be amplified by PCR. Protein however is chemically more robust than DNA and can persist for longer periods. Protein also contains genetic variation in the form of single amino acid polymorphisms. These can be used to infer the status of non-synonymous single nucleotide polymorphism alleles. To demonstrate this, we used mass spectrometry-based shotgun proteomics to characterize hair shaft proteins in 66 European-American subjects. A total of 596 single nucleotide polymorphism alleles were correctly imputed in 32 loci from 22 genes of subjects’ DNA and directly validated using Sanger sequencing. Estimates of the probability of resulting individual non-synonymous single nucleotide polymorphism allelic profiles in the European population, using the product rule, resulted in a maximum power of discrimination of 1 in 12,500. Imputed non-synonymous single nucleotide polymorphism profiles from European–American subjects were considerably less frequent in the African population (maximum likelihood ratio = 11,000). The converse was true for hair shafts collected from an additional 10 subjects with African ancestry, where some profiles were more frequent in the African population. Genetically variant peptides were also identified in hair shaft datasets from six archaeological skeletal remains (up to 260 years old). This study demonstrates that quantifiable measures of identity discrimination and biogeographic background can be obtained from detecting genetically variant peptides in hair shaft protein, including hair from bioarchaeological contexts.

## Introduction

The forensic science and bioarchaeological communities depend on methods, particularly DNA typing, that identify individuals in ways that are scientific and statistically valid[1]. This study provides the scientific basis and seeks to establish the utility of using protein typing as an additional genetic forensic tool. DNA typing has the ability to statistically place individuals at specific locations, to associate them with physical evidence, and to determine biometric and biogeographic genetic information[2–5]. In a bioarchaeological context, ancient DNA allows calculation of biodistance when compared to other samples and existing biogeographic populations[6, 7]. DNA methods depend on the presence of DNA template of sufficient quantity and quality to amplify *via* PCR and produce genotype information for short-tandem repeat loci (STR), single nucleotide polymorphisms (SNPs), or mitochondrial DNA haplotypes[2, 7]. A major limitation of these techniques however, is the susceptibility of DNA to biological, environmental, and chemical processes that reduce template length and modify base structure[8]. These processes result in a loss of template DNA in samples, sometimes beyond the capacity of PCR and sequencing strategies to compensate[9]. In the event that DNA typing yields a partial or null result, few quantifiable genetic alternatives are available to the investigator[1]. Development of identifying technologies, beyond those that depend solely on DNA typing, is a fundamental need for the forensic and bioarchaeology communities[1, 10].

Protein is chemically more stable, abundant, and environmentally persistent than DNA[11–15]. The condition of protein in bioarchaeological samples is commonly used as an indicator of biomolecular integrity. For example, protein yield and carbon-to-nitrogen atomic ratio are considered a necessary, but not sufficient, indicator of the presence of residual endogenous DNA template[11]. Hair keratin, bone collagen, and tooth collagen are now routinely used for ^14^C dating and in stable light-isotope analysis for palaeodietary and related information[16–19]. Significantly, protein contains genetic variation in the form of single amino acid polymorphisms (SAPs) that result from non-synonymous single nucleotide polymorphisms (nsSNPs)[20]. Based on exome analysis, there are over 35,000 nsSNPs with genotype frequencies greater than 0.8% in the European–American (EA) population (Exome Sequencing Project (ESP), evs.gs.washington.edu/EVS/; S1 Fig)[21]. Genetically variant peptides (GVPs) containing SAPs can be identified using mass spectrometry-based shotgun proteomics[20, 22]. Identification of these peptides allows imputation of nsSNP alleles in an individual genome regardless of the presence of DNA template in the sample.

The status of separate imputed nsSNP alleles can be aggregated to provide a profile of genetic variation for a particular individual. The probability of a particular profile occurring in a population can then be estimated by applying the product rule[2, 23]. Overall probabilities vary as a function of genetic background, for reasons including selection, founder effects, genetic drift, and admixture[21, 24, 25]. Therefore, as with STR allele profiles and mtDNA haplotypes, imputed nsSNP alleles can potentially be used to obtain both individualizing and biogeographic information[26–28].

To test the feasibility of protein-based measures of human identification, we focused on the human hair shaft proteome. Hair is often a forensically relevant component of crime scenes and archaeological sites, where it persists under a wide range of environmental conditions[18, 29–31]. The hair shaft is composed primarily of coiled-coil proteins with a high degree of intermolecular disulphide and isopeptide covalent bonds that account for both the physical flexibility and robustness of the structure [32, 33]. Despite the physical properties of hair, it is a poor source of nuclear DNA template due to keratinocyte apoptosis during hair shaft biogenesis, subsequent weathering in life, and biological and environmental processes post-mortem[34, 35]. Regardless of the status of residual nuclear or mitochondrial DNA, hair retains a high protein content and more than 300 proteins have been detected in the hair proteome [36, 37]. This protein population provides a sufficiently broad representation of the genome to test the validity of using proteome-based nsSNP imputation to develop forensically and bioarchaeologically useful measures of identity and biogeographic origin.

## Materials and Methods

### Tissue Procurement

Cranial hair shafts and buffy coat DNA were collected from a cohort of 60 self-identifying unrelated European–Americans (EA1, Sorenson Forensics LLC, Salt Lake City). Genomic DNA from each subject was screened using the Investigative LEAD^™^ Ancestry DNA Test (Sorenson Forensics LLC, Salt Lake City, UT) and genotype data was generated for 190 SNPs that are ‘Ancestry Informative Markers’, which span all 22 autosomal chromosomes[38]. Nine individuals had measurable non-European admixture and were excluded from the analysis (S1 Table). An additional collection was conducted using cranial hair shaft and nuclear DNA from another cohort of self-identified unrelated European–Americans (EA2, n = 15). All material was collected using protocols, informed consents, and questionnaires that were approved by the Institutional Review Boards at Utah Valley University (IRB #00642) and Lawrence Livermore National Laboratory (IRB#11–007). Hair shaft material was also collected from a cohort of five African-American and five Kenyan subjects[39]. Cranial hair shafts were additionally collected from six individuals from two separate archaeological assemblages excavated in London and Kent: three individuals (S1–S3), dating from circa 1750–1850, and three individuals (S4–S6) from a cemetery in active use 1821–1853.

### Proteomic Data Acquisition and Identification of Single Amino Acid Polymorphism-containing Peptides

Hair from subjects was processed physically and biochemically and data was acquired as described (S1 Methods). Briefly, hair was ground or milled; treated in a solution of urea, DTT, and detergent; alkylated; and then proteolyzed with trypsin. Resulting peptide mixtures were analyzed using tandem liquid chromatography mass spectrometry. The resulting proteomic datasets were converted to the Mascot generic format and analyzed using three different approaches: Mascot (software version 2.2.03, Matrix Science, Inc., Boston, MA), X!Tandem, using the GPM manager software (www.thegpm.org, release SLEDGEHAMMER (2013.09.01)), or X!Tandem using the Petunia Graphic User Interface (TANDEM CYCLONE TPP, download = 2011.12.01.1 –LabKey, Insilicos, ISB). A custom protein reference database was used (S1 Methods; https://zenodo.org/record/58223; DOI: 10.5281/zenodo.58223) to ensure the identification of genetically variant peptides by both Mascot and the Petunia GUI peptide spectra matching algorithms[20]. Resulting peptide lists were screened for the presence of genetically variant peptides and identifications were collated for each subject. Imputations made through the use of GPM manager or the use of the customized reference database, in either X!Tandem or MASCOT, were compared for redundancy (S2 Table). The mass spectrometry proteomics data that has been submitted to the Global Proteome Machine (www.thegpm.org, S1 Methods) can be publically accessed (S1 File)[40].

### Validation of Identified Genetically Variant Peptides

Identified candidate genetically variant peptides were filtered to reduce false positive assignment using the following criteria for exclusion: low quality expectation scores (X!Tandem, log(e) < –2; Mascot, expectation score >0.05), if the corresponding nsSNPs were distributed at less than 0.8% in the sample population (minor allelic frequency < 0.4%), the presence of masses in a MS/MS fragmentation spectrum from a GVP consistent with the alternative allele, the incorporation of biological post-translational modifications in the assigned sequence (such as phosphorylation), and high variance between theoretical and observed primary masses (> 0.2 Da). Amino acid polymorphisms assigned due to likely chemical modification or conversion were also excluded from the analysis (www.unimod.org)[41–43]. Rejected single amino acid polymorphisms include methionine to phenylalanine, asparagine to aspartate, glutamine to glutamate and cysteine to serine[41, 43, 44]. Peptides that were potentially derived from paralogous sequences, or that were potentially expressed in more than one gene product, were removed from the analysis (S2 File). Imputed nsSNP loci were directly validated by Sanger sequencing of the subjects’ nuclear DNA (S1 Methods).

### Statistical Treatment of Individual Imputed nsSNP Profiles

An estimation of the probability of a given imputed nsSNP allele profile being detected in a sample population was calculated using a frequentist estimation of allele frequency, or frequency of an allele combination, within the reading frame of a gene (Pr(imputed nsSNP allele gene combination|population)), and a Bayesian application of the product-rule[2, 23]. The occurrence of alleles, or allele combinations, was counted in European (n = 379) and African (n = 246) sample populations (S3–S8 Tables, www.1000genomes.org; Phase 1)[45]. The 1000 Genome Project sample populations were selected as sample populations because the African population did not have European admixture. The final probability of an individual SNP, or SNP combination, occurring within a gene reading frame, was estimated as (x + ½)/(n + 1), where x is the number of individuals with a given SNP, or combination of SNPs, in a sample population of size n[46]. The above expression represents the Bayesian posterior mean of a binomial probability using the Jeffreys Beta (½, ½) prior, which has the advantage of giving a non-zero estimate of the population probability even for x = 0[46, 47]. Full independence between genes was assumed. The effect of observed allele variation on the overall profile probability was estimated by parametric bootstrap resampling from a binomial (n, (x + ½)/(n + 1)) distribution for each gene, multiplying the resulting probability estimates across genes, and taking the 5^th^ and 95^th^ percentiles of the resampling distribution (90% CI)[47]. A comparison of the imputed nsSNP profile probability in the sample European and African population was calculated as a likelihood (L) ratio (L = Pr(profile|EUR population)/Pr(profile|AFR population))[23].

## Results

### Genetically Variant Peptides Can Be Used to Impute nsSNP Alleles

Cranial hair shafts and corresponding buffy coat DNA were obtained from two cohorts of European–American subjects (EA1, n = 51; EA2, n = 15). Peptides were generated from hair shaft material by milling, denaturation, reduction, alkylation, and trypsinization. Proteomic datasets were obtained using liquid chromatography tandem mass spectrometry (LC-MS/MS). Proteomic analysis of the European American cohorts EA1 and EA2 identified, respectively, 182 and 401 proteins that were found in datasets from 15% or more of the individuals in each cohort (S3 and S4 Files). The most abundant proteins identified in each individual proteome were keratins and keratin-associated proteins, but proteomes also consistently included under characterized proteins such as calmodulin-like protein 3, protein S100A3, V-set and immunoglobulin domain-containing protein 8, and selenium-binding protein 1[36, 37]. Consistent with the biogenesis of hair shaft, other protein classes were also detected, although at lower levels[35]. Included were housekeeping proteins, metabolic enzymes, and proteins associated with cellular structures such as the nucleus, mitochondrion, plasma membrane, and lysosome [36, 37]. Across all samples, the total number of peptides detected ranged from 376 to 18,563 ($\overset{-}{x}\pm s$ = 3,270 ± 2,591, median = 2,281) and yields of unique peptide spectral matches ranged from 156 to 2,011 ($\overset{-}{x}\pm s\;$ = 708 ± 355, median = 615).

Publicly available peptide spectral matching software was employed to make sequence database-based peptide identifications (X!Tandem and GPM manager, S1 Methods). A custom reference protein database was developed for use with X!Tandem that contained all single amino acid polymorphisms (SAP) with a greater than 0.4% allelic frequency in either European–American or African-American sample populations (evs.gs.washington.edu/EVS). In the case of GPM manager an open-source database (www.thegpm.org) was used[48]. Genetically variant peptides (n = 89) containing SAPs from 53 SNP loci in 33 genes (S9 Table) were identified in each individual proteomic dataset and collated for each individual (S5–S7 Files).

**Fig 1:**
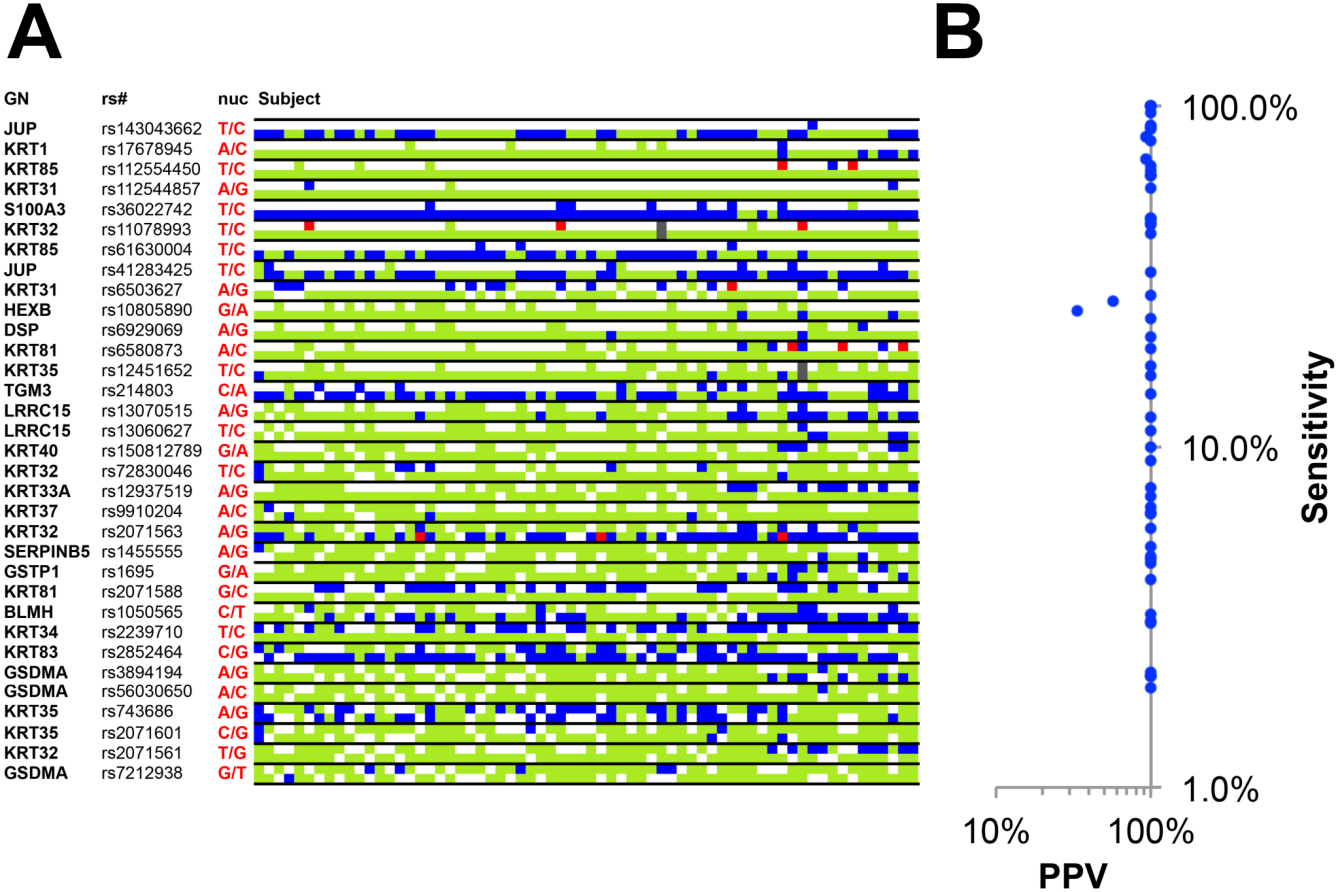
Direct validation of imputed non-synonymous SNP alleles. **A**) Genetically variant peptides (GVPs) that contained single amino-acid polymorphisms (SAPs) were identified in both European-American cohorts (EA1 and EA2) and collated for each subject. Imputed nsSNP alleles (Gene Name = GN, SNP accession number = rs#, allele nucleotide = nuc) were directly compared to the genotype resulting from direct Sanger sequencing (S1 Methods). Correctly imputed nsSNP alleles (TP, true positives) are indicated by a blue square. Imputed alleles that were incorrectly predicted (FP, false positive) are indicated by red squares. Alleles that were identified using Sanger sequencing, but did not contain a resulting GVP in the matching proteomic dataset (FN, false negative) are indicated by light green squares. Alleles absent in both subjects DNA and in resulting proteomic datasets (TN, true negatives) are indicated by white squares[49]. Failed Sanger sequencing determination of nsSNP allelic status is indicated by grey. **B**) The effectiveness of each SAP-containing peptide to impute nsSNP alleles was also quantified. The sensitivity of each genetically variant peptide, measured as the proportion of nsSNP-alleles that are correctly detected and imputed (TP/(TP+FN)), was calculated as a percentage (log_10_(%). The positive predictive value (PPV) of genetically variant peptide-based SNP imputations was calculated as the percentage of correct validated SNP imputations of all imputations (TP/(TP + FP); log_10_(%))[49]. **C**)

Direct validation of SAP-containing, genetically variant peptide (GVP) was then conducted through Sanger sequencing of 32 loci in 22 genes of the subjects’ DNA (S2 and S10 Tables). The genotype at each non-synonymous SNP locus for each individual was collated and compared to the imputed alleles based on identification of GVPs in proteomic datasets. A total of 608 imputed genotype determinations were made (Fig 1A, S2 Fig, S2 and S10 Tables) of which 596 were true positives (TP) that were confirmed with DNA sequencing (blue squares) and 12 were false positives (FP, red squares)[49]. Alleles that were not represented by GVPs in the proteomic datasets (FN, false negatives) were indicated with light grey squares. The false discovery rate (FP/(FP+TP) was 1.98% and the overall positive predictive value (PPV, TP/(TP+FP)) was 98.3%. The sensitivity of each genetically variant peptide, defined as the portion of correct imputations made out of all possible imputations (TP/(TP+FN)) and was calculated, along with positive predictive value (PPV), for each individual GVP (Fig 1B, S11 Table) [49]. Only 5 peptides had positive predictive values that were not 100%, whereas sensitivity (TP/(TP+FN)) ranged widely.

### Estimation of Individual Imputed nsSNP Profile Probabilities

**Fig 2:**
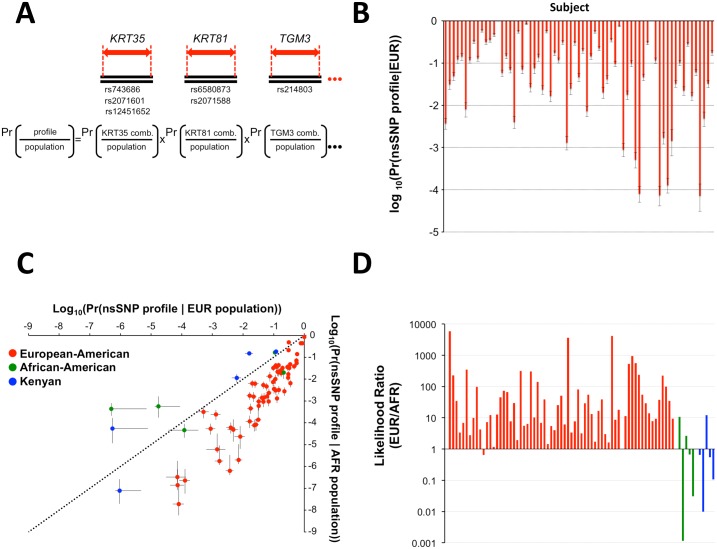
Imputed nsSNP profile probabilities in European and African populations. **A**) The probability of an overall individual nsSNP profile in the population (Pr(profile|population)) was estimated by determining the probability of detected nsSNP alleles, or allele combination, in each gene (Pr(nsSNP gene profile|population)), and then using the product rule to multiply these probabilities together (Pr(overall profile|population)). **B**) The probability of overall imputed nsSNP profiles occurring in the European population (Pr(profile|EUR population)) was calculated using imputed nsSNP alleles from individuals in the two European-American cohorts (EA1 and EA2) and the product rule. Values are presented as a logarithm (log_10_(Pr(profile|EUR population))). Confidence intervals (90% CI) are estimated using parametric bootstrapping. **C**) The overall imputed nsSNP profile probability in the African population was also calculated (Pr(profile|AFR population)) and plotted versus the probability of the profile occurring in the European population (Pr(profile|EUR population)). Confidence intervals (90% CI) were estimated using parametric bootstrapping. In addition to European–American subjects (red), imputed nsSNP profile probabilities were also estimated from proteomic datasets derived from an African-American (green) and Kenyan (blue) cohort. The line of equal profile probability in the European and African population is indicated (dotted line). **D**) The likelihood of hair samples coming from a European relative to African genetic background was calculated as the ratio of overall imputed nsSNP profile probabilities in the European and African populations (EUR/AFR = Pr(profile|EUR population)/Pr(profile|AFR population)); European-American subjects (red), African-American subjects (green), and Kenyan subjects (blue) are indicated.

The aggregate of identified SAP-containing genetically variant peptides represents a considerable degree of genetic variation. If the imputed individual nsSNP profiles are present at a sufficiently low proportion in the population, they can be useful to forensic investigators or archaeologists. To estimate the probability of individual nsSNP profiles in the population, a modification of the product rule was used. The observed number of SAP alleles, or combination of alleles, within an open reading frame of a gene, was counted in a sample population to estimate the probability of each allele occurring in that population. The product of all detected alleles, or allele combinations, was used to estimate the probability that the overall imputed nsSNP profile occurred in the sample populations (Fig 2A). When estimated using a sample European population, the resulting overall profile probabilities ranged from 9.98 x 10^−1^ to 7.21 x 10^−5^ ($\overset{-}{x}\pm s$ = 1.65 x 10^−1^ ± 2.20 x 10^−1^, median = 7.26 x 10^−2^) (Fig 2B). To model stochastic sampling effects, confidence intervals (90%) for the imputed nsSNP profile probabilities were estimated by parametric bootstrap resampling[47]. Imputed nsSNP profile probabilities improved exponentially as a function of proteomic dataset quality (r = 0.6811, n = 51, p < 0.001; S3 Fig).

### Estimation of Individual Imputed nsSNP Profile Probabilities in Other Populations

The allelic probabilities of many SNPs show considerable variation among populations[50–54]. When the probability of the overall imputed nsSNP profile was estimated using frequencies of nsSNP alleles in the sample population of African individuals, the profile probabilities decreased to a range of 8.56 x 10^−1^ to 1.90 x 10^−9^ ($\overset{-}{x}\pm s$ = 5.03 x 10^−2^ ± 1.41 x 10^−1^, median = 3.37 x 10^−3^). This indicated that the observed profile probabilities in the sample African population were lower compared to those in the sample European population (Fig 2C). This is consistent with the biogeographic origin of the subjects. When datasets from African-American and Kenyan individuals were also analyzed, and estimates of imputed nsSNP profile probabilities obtained for both populations, different probability patterns emerged. Contrary to imputed nsSNP profiles from European–American donors, the profile probabilities of some African American and Kenyan individuals were less frequent in the European relative to the African population (Fig 2C). Both populations contained individuals that distributed in the probability space close to the line of equal likelihood. When quotients of the values for each individual were calculated, likelihood ratios were obtained for the European relative to African populations (L = Pr(profile|EUR population)/Pr(profile|AFR population)). European-American hair shaft protein samples produced likelihood ratios that ranged from 6.50 x 10^−1^ to 5.85 x 10^3^ ($\overset{-}{x}\pm s$ = 2.82 ± 9.72 x 10^2^, median = 1.50 x 10^1^, Fig 2D). Likelihood ratios derived from African-American and Kenyan samples ranged from 1.07 x 10^1^ to 1.15 x 10^−3^ and 1.21 x 10^1^ to 9.9 x 10^−3^ respectively (Fig 2D). This observation indicates that imputed nsSNP allele profiles derived from hair shaft proteins have the potential to provide quantifiable statistical information about the relative biogeographic ancestral background of individuals.

### Comparison of Profile Probabilities from Imputed nsSNPs and Mitochondrial DNA Haplotypes

**Fig 3:**
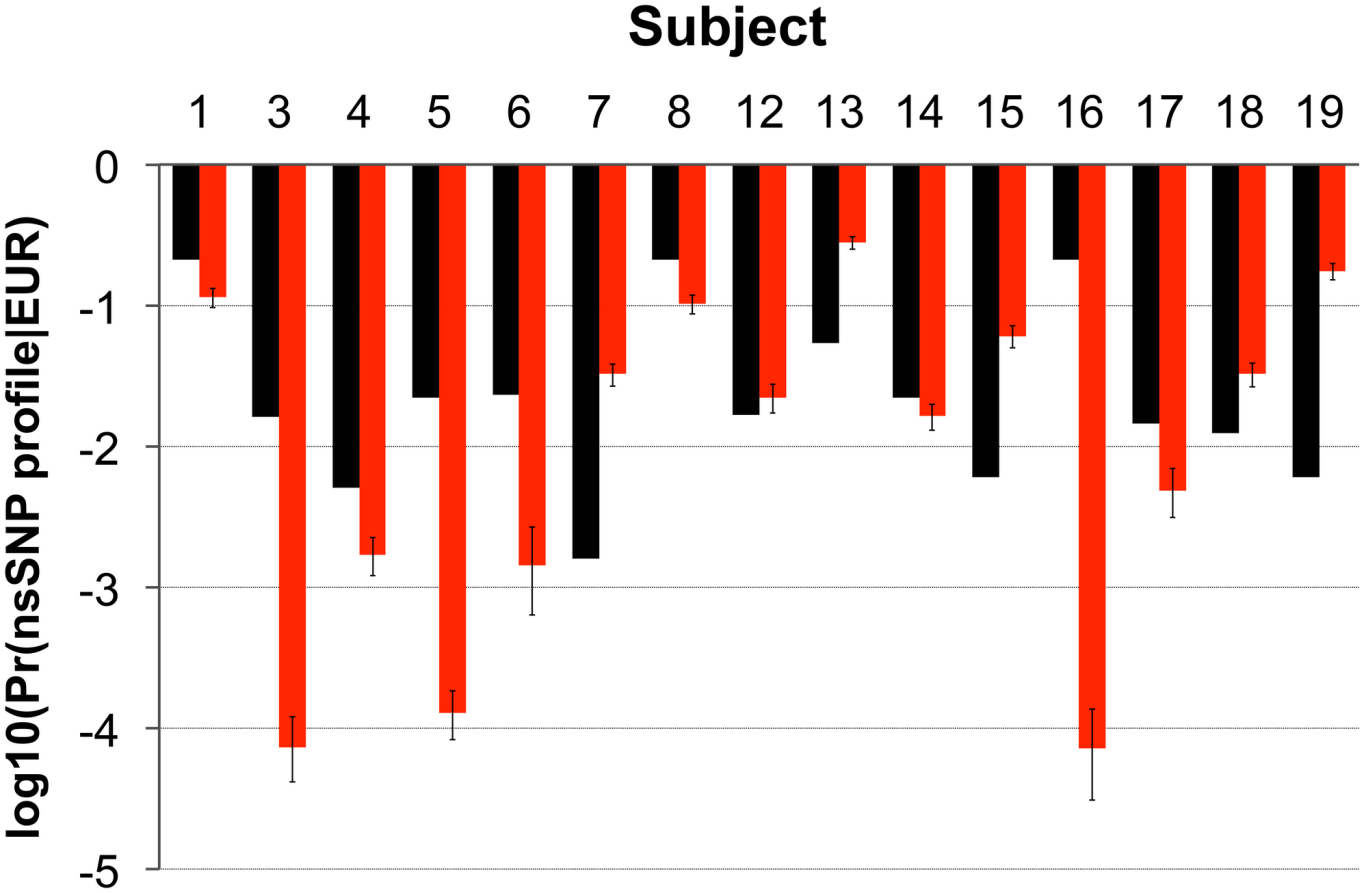
Comparison of probability estimates based on imputed nsSNPs and mitochondrial DNA haplotype. The mitochondrial DNA haplotype and subgroup from one of the European-American cohorts (EA2, n = 15) were classified, compared to a database of subjects from an American sample population (Utah, n = 9,372), and the logarithm of haplotype probability was calculated (log_10_(Pr(mtDNA haplotype|Utah population)), black bars). Genetically variant peptides containing single amino acid polymorphisms were identified in the hair shaft proteomic datasets of the same subjects, an overall profile of imputed nsSNP loci determined, and logarithm of the probability of each profile occurring in the European population was calculated as described in the Materials and Methods section (log_10_(Pr(imputed nsSNP profile|EUR population)), red bars). Confidence intervals (90% CI) were estimated using parametric bootstrapping. Each measure is represented using the same axis (log_10_(Pr(profile|population))).

While DNA is degraded as a function of biological processes, mitochondrial DNA has a higher template number than nuclear DNA and is more likely to survive apoptotic and subsequent environmental processes[35]. The current best practice to gain forensically informative genetic information from hair shafts is to obtain the mitochondrial DNA haplotype and determine the probability of occurrence in reference sample populations[55]. Cranial hair shafts and buffy coat DNA were collected from a cohort of European-American subjects (EA2) and mitochondrial haplotypes obtained by sequencing the D-loop of mitochondrial DNA. The probability that each mitochondrial sub-clade haplotype would be observed in a database of a Utah sample population (n = 9,372) was estimated and ranged from a value of 2.13 x 10^−1^ to 1.60 x 10^-3^ ($\overset{-}{x}\pm s$ = 5.59 x 10^−2^ ± 8.21 x 10^−2^, median = 1.66 x 10^−2^) (Fig 3, S12 Table). The probability of individual imputed nsSNP profiles ranged from 2.80 x 10^−1^ to 7.21 x 10^−5^ ($\overset{-}{x}\pm s$ = 5.63 x 10^−2^ ± 8.10 x 10^-2^, median = 2.22 x 10^−2^) in the same cohort (Fig 2B). In most subjects (9 out of 15), profiles of genetically variant peptides were more discriminatory than mitochondrial haplotypes.

### Changes in the Proteomic Profile as a Function of Taphonomic Processes

**Fig 4:**
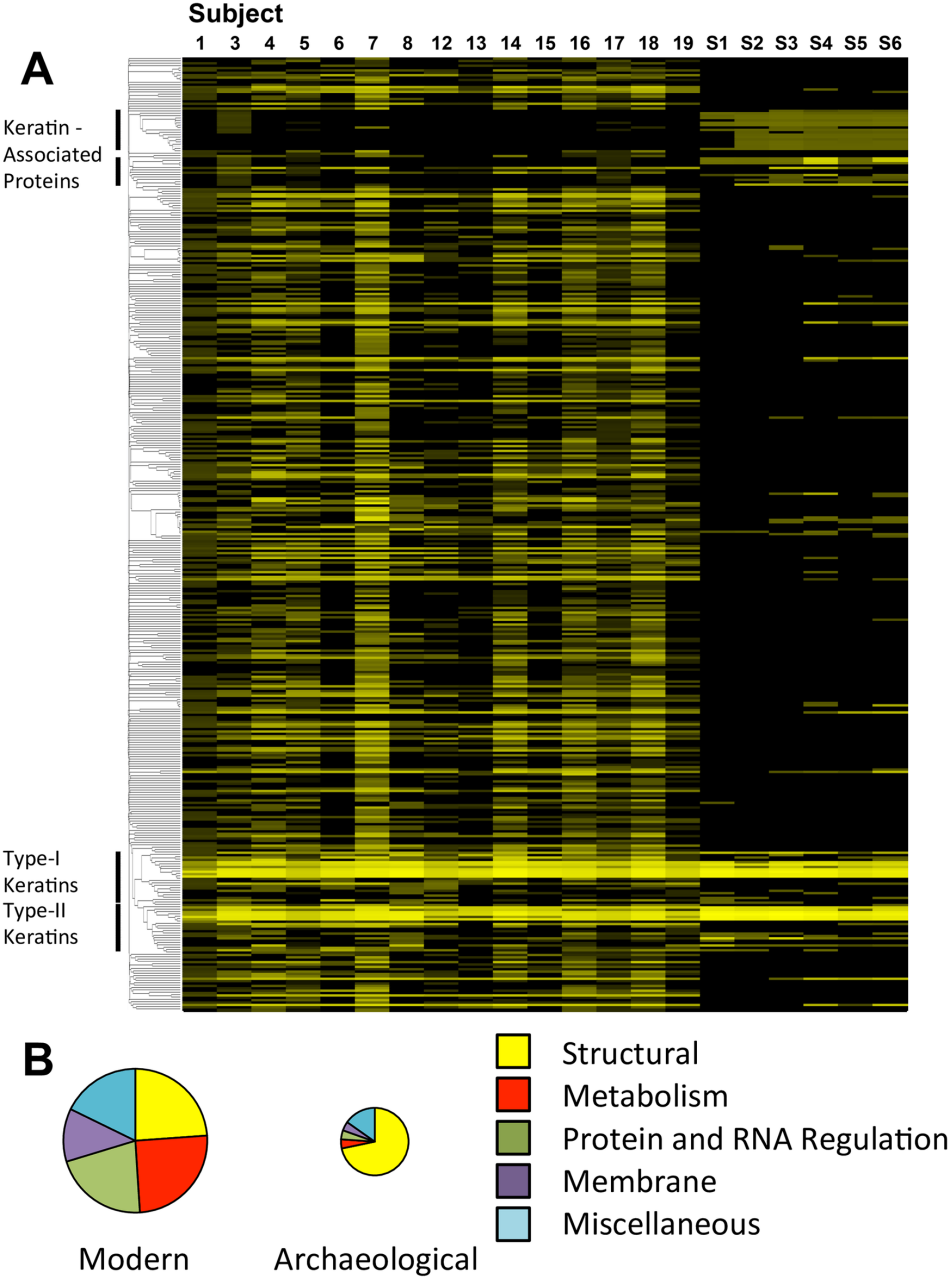
Hair shaft proteomic profile in modern and archaeological samples. **A**) Absolute protein abundance from all datasets corresponding to a cohort of European-American subjects (EA2, subjects 1 to 19) and archaeological subjects (S1 to S6) was measured (www.thegpm.org) and collated. Proteins that appeared in proteomic datasets of 15% or more of the subjects (n = 401) were aligned as a paralogous neighbor-joining tree in order to cluster detected proteins with higher levels of homology (www.uniprot.org.). The neighbor-joining tree based on protein paralogy is aligned on the vertical and subjects on the horizontal. Protein abundance is indicated by conditional formatting (maximum value = yellow, minimal value = black). **B**) The function of individual proteins was obtained (www.uniprot.org) and collated for both modern (EA2, 1 to 19) and archaeological (S1 to S6) hair shaft samples (categories = structural, metabolism, protein and RNA regulation, membrane proteins, and miscellaneous). The relative abundance of the different protein classes is indicated by area. The size of each circle is proportional to the relative abundance of total detected peptides in each sample class.

Six archaeological hair samples were collected from the area of London and Kent: three individuals (S1-S3), dating from circa 1750–1850, and three individuals (S4-S6) from a cemetery in active use from 1821 to 1853. The samples were ground, reduced and alkylated, and treated with trypsin in the presence of Protease-Max (Promega) or deoxycholate (S1 Methods). Digests from 1 mg of sample were analyzed by LCMS/MS on a high-resolution qToF, and the resulting data processed using X!Tandem and an open-source database (www.thegpm.org). Absolute protein levels in the hair shaft proteome, determined by the frequency by which expected peptides appeared in a dataset, were collated and values summed for each individual in one of the European-American (EA2, n = 15) and archaeological cohorts (n = 6) (www.thegpm.org)[56]. Proteins that were found in proteomic datasets from 15% or more of individuals in the cohort were arranged in a neighbor-joining tree based on sequence homology (y-axis), and their abundance indicated through conditional formatting with yellow color (Fig 4A). There was a significant reduction in hair proteome complexity in the archaeological samples. The reduction in complexity of the proteome in these samples results in greater proportional representation of remaining proteins, mainly trichocyte keratins (Types I and II), and cysteine-rich keratin-associated proteins. Non-structural proteins were apparently degraded or removed through environmental processes (Fig 4B)[15]. This is consistent with the observation that microfibrillar structures, and particularly the sulfur-rich inter-microfibrillar matrix, persist longer in the environment relative to other internal anatomical components of the hair shaft[57].

### Detection of Genetically Variant Peptides in Archaeological Hair Samples

**Fig 5:**
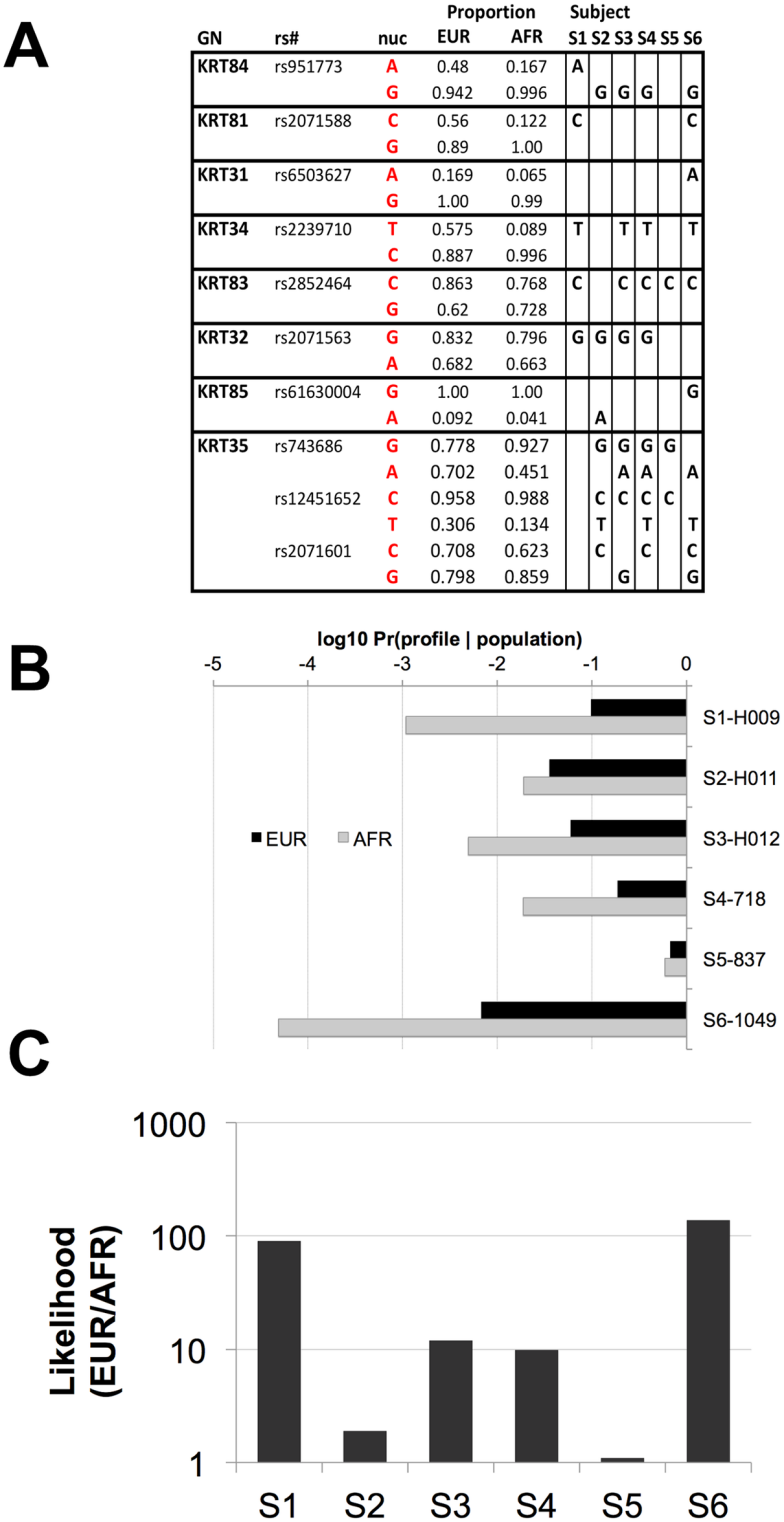
Imputed nsSNP loci in archaeological hair shaft proteomes. **A**) Hair was obtained from six individuals from two separate post-medieval archaeological assemblages excavated in London and Kent (S1 to S6) and proteomic datasets obtained (S1 Methods). Peptides containing single amino acid polymorphisms (Gene Name; GN) were identified, collated, and nsSNP loci and alleles imputed (dbSNP identifier and nucleotide = rs# and nuc) in Subjects S1 to S6. The proportion of each allele in the European (EUR) and African (AFR) population is included. **B**) The overall imputed nsSNP profile probability (Pr(profile|population)) in the European (EUR, black bars) and African (AFR, grey bars) population was calculated as the product of imputed nsSNP, or combination of nsSNP, probabilities for each gene. **C**) Likelihood measurements of European compared to African genetic origin were calculated as a quotient of overall imputed nsSNP profile frequencies (Pr(profile|EUR population))/(Pr(profile|AFR population)).

Peptides containing SAPs were identified in each dataset and collated for each individual archaeological sample, and the profile of nsSNP alleles was imputed (Fig 5A). The probability of each imputed nsSNP profile was estimated. The values ranged from 6.69 x 10^−1^ to 6.76 x 10^−3^ ($\overset{-}{x}\pm s$ = 1.76 x 10^−1^ ± 2.49 x 10^−1^, median = 7.85 x 10^−2^) (Fig 5B). When the same calculations were conducted using occurrence of nsSNPs in the African population, profile probabilities were relatively less, ranging from 5.91 x 10^−1^ to 4.90 x 10^−5^ ($\overset{-}{x}\pm s$ = 1.06 x 10^−1^ ± 2.38 x 10^−1^, median = 1.19 x 10^−2^) (Fig 5B). The likelihood ratio of nsSNP profile probabilities from the European and African population ranged from 1.13 x 10^0^ to 1.38 x 10^2^ ($\overset{-}{x}\pm s$ = 4.22 x 10^1^ ± 5.78 x 10^1^, median = 1.10 x 10^1^) (Fig 5C). The positive likelihood values indicate that the imputed nsSNP profiles are more common in the European population, which was consistent with the archaeological location of the hair samples.

## Discussion

Genetically variant peptides that contain single amino acid polymorphisms (SAP) detected in hair shaft proteomic datasets were used to impute the status of SNP alleles in subject genomes. An estimate of the proportion of the European population containing the overall imputed non-synonymous SNP (nsSNP) profile was then calculated using the product rule. Based on differences in imputed nsSNP allelic frequencies in different genetic backgrounds, likelihood measurements were calculated for European relative to African genetic backgrounds, with distinct patterns emerging as a function of genetic background. The resulting nsSNP allele profile probabilities were of the same order of discrimination as mtDNA haplotypes. When the approach was extended to bioarchaeological hair samples, these individual measures of discrimination and likelihood of biogeographic background, were also obtained.

There is a long history of using hair shafts for anthropologic and forensic analyses[58]. Recently hair shafts collected from an extinct Paleo-Eskimo (~4,000 yr BP) and an Australian Aboriginal (~100 yr BP) were used to obtain complete mitochondrial and nuclear genomes[59, 60]. These are exceptional cases using gram quantities of hair; most hair shafts are a poor source of nuclear DNA, and obtaining full STR-profiles is problematic and not routinely recommended by the Scientific Working Group on Materials Analysis (SWGMAT)[34, 35, 61–64]. Current best practice includes sequencing of hair shaft mitochondrial DNA to identify haplotype and sub-clade. This method provides identification and biogeographic information (Fig 3), but is less discriminating than STR-typing, requires careful handling and sequencing, and is susceptible to environmental factors[55, 65, 66]. Other hair shaft-based forensic methods can be problematic. Microscopic hair comparison, while heavily used historically, does not have the potential for rigorous statistical and scientific analysis[1, 29, 62, 67, 68]. Previous attempts to use abundance patterns of solubilized hair proteins in two-dimensional electrophoresis protein gels were insensitive, irreproducible, and proved susceptible to environmental factors[69–71]. However, the relative abundance patterns of expressed proteins in proteomic datasets have been used to develop measures of biodistance in mouse lines and human genetic groups[39, 72].

The ability of a single amino acid polymorphism (SAP) to impute the status of a non-synonymous single nucleotide polymorphism (nsSNP) assumes that only one SNP accounts for the change in protein primary structure and vise versa. Clearly there is degeneracy in the genetic code and more than one nucleotide change can account for a given amino acid. However, the GVPs analyzed in this study originate from one position on the genome and genetic databases allow for accurate estimation of the distribution of a particular SNP in a sample population. The SNPs analyzed in this study are common (MAF > 0.8%) and, with some exceptions, widely distributed across all current human populations[24, 73, 74]. The originating random nucleotide mutations analyzed in this study occurred in an ancestor to all extant human populations, possibly even pre-dating the emergence of anatomically modern traits[24, 75]. While theoretically another novel mutation may account for an identical single amino acid polymorphism, the probability of this event would be highly rare and unlikely. Of the SNPs characterized in this study there is no evidence of a tri-allelic SNP where two alleles account for a single amino acid polymorphism. Because the imputation is based on the observation of GVPs, the genotype, instead of the allelic, frequency is the appropriate basis of estimating probability. The probabilities of both corresponding GVPs, major and minor allele, will always have a sum that is greater than one (S9 Table). Other mechanisms also have the potential to prevent imputation of SNP alleles based on detection of GVPs. Chemical or biological modification of a peptide may potentially result in mass shifts at specific amino acids that may correspond to the mass shift of a genetically caused single amino acid polymorphism. This contingency is dealt with by focusing on genetically variant peptides that result from common nsSNPs, which are more likely, eliminating amino acid polymorphisms that have the same mass shift as commonly occurring peptide modifications, and excluding fragmentation mass spectra that show signatures of chemical modification or fall below quality thresholds.

Identification of peptides in a tandem LC-MS/MS dataset depends on peptide spectral matching software that statistically compares peptide collision-induced dissociation (CID) fragmentation spectra with masses derived from a theoretical tryptic peptide amino acid sequence in a protein reference database[76–78]. Standard databases, such as the RefSeq or UniProt protein database, consist solely of reference protein sequences resulting in the absence of non-reference variant alleles in the resulting assigned peptide lists. Databases therefore need to be customized to contain all possible SAPs. Large comprehensive databases, however, are highly inefficient and result in loss of sensitivity[76, 78, 79]. The approach used in this study balanced these factors and generated a customized database containing an additional sequence of each reference protein but with the inclusion of all SAPs with an allelic frequency of greater than 0.5% in either the European or African populations in a single protein sequence[76, 78, 79]. The removal of rare (MAF < 0.5%) nsSNPs from the analysis decreased the likelihood of false positive assignment, in which a mass shift at a point on a peptide may be falsely attributed to a relatively unlikely genetic, as opposed to chemical or biological, mechanism. Further refinements to the reference protein databases, generation of spectral databases from synthetic peptides, and search strategies incorporating *de novo* protein sequencing and redundant search engines will all result in greater sensitivity, predictability, and efficiency of genetically variant peptide identification[80–83].

The ability of detected SAP-containing peptides to accurately impute the status of corresponding nsSNP alleles was tested through direct Sanger sequencing of each subject’s DNA. Almost all peptides had positive predictive values of 100%, indicating that GVPs can accurately impute the associated SNP allele in a subjects’ genome. Naturally for GVPs with a high genotype frequency, or high prevalence, a high predictive value is less informative[49, 84]. Some apparent SAP-containing peptides, however, were false-positive assignments that fell into two categories: those with no or few correct assignments (KRT85_D189N, KRT32_R369Q), and those that were highly sensitive and specific but with an occasional false-positive assignment (KRT31_A82V, KRT32_T395M). The former category was not used for probability estimation. The latter category requires a complete replication of the analysis and comparison with data obtained from synthetic peptides. The sensitivity of SAP-containing peptides to detect the status of an nsSNP allele ranged broadly. Sensitivity values (TP/(TP+FN)) will increase as sample processing and data acquisition protocols improve, with better instrumentation, and refinements in bioinformatics processing[49]. Reduction of sample size to a single hair is a necessary, and we believe achievable, requirement for forensic casework analysis and physical anthropology fieldwork samples.

To estimate the probability that an overall individual nsSNP profile is present in a given population, two steps were taken (Fig 2A). Firstly, the probability of detected nsSNP alleles, or combination of nsSNP alleles, in each gene (Pr(nsSNP gene combination|population)) was estimated by directly counting the occurrence of each gene profile in the sample population and dividing by the sample size, a statistically frequentist approach that makes no assumptions about dependencies within the gene boundary (www.ensembl.org)[23]. This was refined using a Bayesian posterior mean of a binomial probability using the Jeffreys Beta (½, ½) prior, which has the advantage of giving a non-zero estimate of the population probability even when the nsSNP allele is not present in the sample reference population[46, 47]. Secondly, the probabilities of imputed nsSNP alleles in each gene were then multiplied together to provide an estimate of the overall imputed nsSNP profile in the population (Pr(profile|population)). The Bayesian use of the product rule in this context assumes independence between the genotype status of nsSNP allele, or allele combinations, in one gene and those in other genes. The trichocyte keratin genes reside in two clusters on chromosomes 17 (Type I keratins) and 12 (Type II keratins) that are roughly 140 kb and 300 kb long respectively[85–87]. Some of these genes therefore are within the typical linkage disequilibrium range of 60 kb[88]. A formal study of linkage dependencies therefore needs to be conducted. One solution would be to extend the boundaries of linkage disequilibrium to incorporate the whole gene cluster and account for evolutionarily conserved haplotypes.

Each estimate of nsSNP allele probability, and consequently imputed nsSNP-profile probability, exists within a confidence interval surrounding the sample value. To approximate the effect of a binomial distribution of allelic occurrence in the sample population on the overall imputed nsSNP-profile probability, a parametric bootstrapping approach was used, to provide a confidence interval surrounding the calculated profile probability[23, 46, 47, 89–91]. Application of the these calculations to proteomic data obtained from a forensic context requires an understanding of underlying population genetics[50]. For the purposes of developing match probabilities, ideally nsSNPs would be selected that are uniformly distributed across all populations. However selection is necessarily restricted to SNPs represented in proteomic datasets. The most conservative approach therefore would be to use the highest, least discriminating, probability derived from candidate genetic groups.

The individual power of discrimination obtained by this method currently is roughly equivalent to that obtained using mtDNA haplotype analysis, the current best practice for obtaining genetic information from hair shafts (Fig 3, S12 Table). Ideally incorporation of both measures into a single measure of discrimination, or for that matter incorporation with partial STR-profile probabilities, would maximize the probative value of hair shafts. Both imputed nsSNP profile probabilities and mtDNA haplotype probability have non-uniform biogeographic distributions, so some statistical dependence is likely[92]. Elucidation of dependence patterns is necessary to integrate the results of both methods, which may be become possible with the advent of larger cohorts of high quality genomic datasets. Integration of imputed nsSNP profile probabilities with partial STR-based DNA typing profiles would be easier since both are autosomal.

The utility of the method on compromised samples was demonstrated on archaeological hair samples that were up to 250 years old. Approximately 1 mg of sample was used to obtain the datasets used in that analysis (S1 Methods). Environmental chemistries and taphonomic processes reduced the complexity of the proteome derived from the sample, and consequently reduced the scope of proteins available for imputed nsSNP loci analysis. This effect was alleviated by increased protein coverage of remaining keratins, and analyses were still able to provide usable estimates of probability and allow comparison of profile probabilities in other biogeographic populations.

This study explores the theoretical and practical basis for using identification of SAP-containing peptides in proteomic datasets to impute nsSNP alleles in an individual’s genome. The resulting profile of imputed nsSNP alleles allows an estimation of the probability that a given profile exists in the population and allows likelihood measures of biogeographic background[93]. Additional steps need to be taken for the method to be applied in a forensic, as well as bioarchaeological, context[94]. Sensitivity needs to increase to the point where sufficiently discriminating information can be obtained from a single hair, or fraction of a single hair, to justify consumption of valuable or legally relevant samples. Statistical treatments of the nsSNP loci used in the study need formal independent validation. With the exception of DNA analysis no forensic method has been rigorously shown to have the capacity to consistently, and with a high degree of certainty, demonstrate a connection between evidence and a specific individual or source[1]. The use of SAP-containing peptides to impute the allelic status of non-synonymous SNPs provides the potential for a complementary and, if necessary, alternative method for use in forensic and bioarchaeological practice.

## Supporting Information

S1 Fig Observation density of missense SNPs in exomes of European-American and African-American individuals. Missense SNP variants (nsSNP) were identified and counted in the NHLBI Exome Sequencing Project (ESP) database (Exome Variant Server, NHLBI GO Exome Sequencing Project, evs.gs.washington.edu/EVS/) [accessed August 1, 2013]. The Exome Variant Server contained 748,407 nsSNPs in the European–American (red) and/or African-American population (green). Counts of minor alleles (nsSNP #) at, or above, indicated frequencies (Minor Allele Frequency (%)) are plotted.
(TIFF)

S2 Fig Validation of imputed non-synonymous SNP profiles. Genetically variant peptides (GVPs) that contained single amino-acid polymorphisms (SAPs) were identified in both European-American cohorts (EA1 and EA2) and directly evaluated for the ability to impute non-synonymous SNP loci in corresponding subjects’ DNA (Gene Name = GN, SNP accession number = rs#). Imputed nsSNP alleles (allele nucleotide = nuc) were directly compared to the genotype resulting from direct Sanger sequencing (S1 Methods). Correctly imputed nsSNP alleles (TP, true positives) are indicated by a colored square containing the respective nucleotide. Genetically variant peptides identified using X!Tandem and a customized database are indicated by yellow. Peptides identified using the GPM manager are indicated by blue, with redundant identifications indicated by green. False-positive identification (FP) is indicated by red squares. Alleles that were identified using Sanger sequencing, but did not contain a resulting GVP in the matching proteomic dataset (FN, false negative) are indicated by pink. Alleles absent in both subjects DNA and in resulting proteomic datasets (TN, true negatives) are indicated by white squares[49]. Failed Sanger sequencing determination of nsSNP allelic status is indicated by grey. Genetically variant peptides that could not be localized to a single genomic locus, could not be used for imputation and are not shown. Genetically variant peptides are sorted based on increasing proportion of the minor allele in the European Population (1000 Genome Project, phase 1).
(TIFF)

S3 Fig Imputed nsSNP profile probability as a function of proteomic dataset quality. The power of discrimination, or proportion of overall imputed nsSNP profiles in the European population (Pr(imputed nsSNP profile|EUR population)), was calculated for each European-American subject (EA1, S1 Methods), and plotted against the corresponding number of unique peptides identified in the proteomic dataset (red circles). Confidence intervals (90%) were calculated using parametric bootstrapping (S1 Methods). To guide the eye, a line indicating exponential regression is also plotted (y = 1.73e^−0.005x^, r = 0.6811, P < 0.0001).
(TIFF)

S1 File Publically accessible proteomic datasets of hair shaft trypsin digests. Mass spectrometry datafiles in either MzML or Mascot Generic Format (mgf) were submitted to the Global Proteome Machine (www.thegpm.org) for peptide spectra matching using the X!Tandem algorithm (X! Tandem Piledriver (2015.04.01.1)). Default search parameters were used including use of the GRCh38 (ENSEMBL) male reference protein database, complete carbamidomethylation of cysteine (C+57), and potential modification of asparigine (N) and glutamine (Q) residues by deamidation (N+1, Q+1) and methionine (M+16) by oxidation. Non-default parameters that were used include the use of the point mutation (sAPS) function and inactivation of the anonymous function. Processed data files in XML format are anonymously accessible using the Global Proteome Machine accession numbers (GPM#) provided.
(PDF)

S2 File Analysis of nsSNP loci for uniqueness and paralogy. Peptides that occur in more than one gene product cannot be used for imputation. Every peptide therefore was analyzed by submission to the PROWL website for protein information (http://prowl.rockefeller.edu/prowl/proteininfo.html) and searched against the IPI human database. Only peptides with a match to a single gene product, or no matches, were accepted as unique. Additional scrutiny, specifically the elimination of the possibility of false polymorphism due to paralogy, was conducted by submitting each sequence to a tblastn search (http://blast.ncbi.nlm.nih.gov/Blast.cgi) and analyzing the resulting sequence alignments. In the event that a tblastn search did not conclusively eliminate the possibility of false paralogy (as is the case with rs114488848, rs140635030, rs139895699) then each wild type peptide sequence was submitted to the PROWL database, number of gene products containing the sequence identified and the presence, or absence, of each polymorphism examined using the ESP exomic database (http://evs.gs.washington.edu/EVS/). In each case where we could not conclusively eliminate false polymorphism, there was only one gene product containing the polymorphism. However, at this stage we cannot formally exclude the possibility that some polymorphisms may also exist in pseudogenes.
(PDF)

S3 File Hair proteome. Datasets from a subset of European–American Subjects (EA1, L1.001 to L1.060) were processed for absolute abundance values using the X!Tandem algorithm (www.thegpm.org) and sorted according to the absolute abundance values in the proteome. Overall abundance values were generated by the following formula: abundance values were averaged and multiplied by the quotient of number of datasets with the detected gene product by the total number of datasets (abundance; n = 54). Each gene product (Ensembl Accession) and proportion of individuals with the detected gene product (count) are described. Primary protein accession numbers (primary acc#), and the mnemonic identifier of a UniProtKB entry was entered (UNIPROT#), along with protein name (protein name) and gene name (GN). Duplicate entries were pooled. Gene products that were detected in less than 7 individuals were not analyzed.
(PDF)

S4 File Hair proteome in modern and archaeological European subjects. Using a population European–American subjects (EA2, n = 15) and the archaeological samples (n = 6) absolute abundance measurements were obtained using the X!Tandem algorithm (www.thegpm.org) and sorted according to the overall abundance values in the proteome. Overall abundance values were generated by the following formula: abundance values were averaged and multiplied by the quotient of number of datasets with the detected gene product by the total number of datasets examined (n = 21) and averaged across all datasets obtained for each individual. The number of subjects where each gene product was observed was determined (observations). Corresponding Primary Protein Accession numbers (Accession #), gene names (GN), Uniprot identifier (UniProt ID) and Ensembl Accession numbers (Ensembl Accession #; www.ensembl.org) are included. The function of each gene product, as recorded in the UniProt database (www.UniProt.org) was also determined and included (fn: s = structural, m = metabolism, mt = mitochondrial, pr = protein regulation and turnover, pm = plasma membrane, mb = membrane associated protein, ml = melanosome associated protein, l = lysome associated protein, u = unknown and miscellaneous, ex = extracellular protein, n = nuclear protein.) Duplicate entries were pooled.
(PDF)

S5 File Imputation of nsSNPs alleles in individual European American (EA1) datasets. Datasets resulting from application of tryptic digests were analyzed using both the Trans Proteomic Pipeline and GPM manager, as outlined in the Supplemental Methods (S1 Methods). Proteomic datasets from a cohort of European–Americans (EA1, n = 51), were analyzed and peptides that contained characterized single amino acid polymorphisms were identified, collated, and summed for each individual. Peptide sequences are included with amino acid polymorphisms indicated in lower case (pept). Single nucleotide polymorphisms that account for the change in amino acid structure are represented in the table by gene name (GN), and dbSNP identifier and allele (rs#_nuc). Multiple alleles occurring within the gene boundary, either through heterozygosity or multiple SNPs are also indicated. The number of observations of alleles, or combination of alleles within a gene boundary, are recoded for both the European (EUR; n = 379) and African (AFR; n = 246) populations (1000 Genomes Project; 1000genomes.org). If a SAP-containing peptide was identified in any of the proteomic datasets associated with an individual, this was indicated by a "1" in the matrix. False positives, identified by genotyping have been removed. A maximum of 1 observation of allele, or combination of alleles, occurs per gene.
(PDF)

S6 File Imputation of nsSNPs alleles in individual European American (EA2) datasets. Datasets resulting from application of tryptic digests were analyzed using both the Trans Proteomic Pipeline and GPM manager, as outlined in the Supplemental Methods (S1 Methods). A cohort of European–Americans (EA2, n = 15) were analyzed and peptides that contained characterized single amino acid polymorphisms were identified, collated, and summed for each individual. Peptide sequences are included with amino acid polymorphisms indicated in lower case (pept). Single nucleotide polymorphisms that account for the change in amino acid structure are represented in the table by gene name (GN), and dbSNP identifier and allele (rs#_nuc). Multiple alleles occurring within the gene boundary, either through heterozygosity or multiple SNPs are also indicated. The number of observations of alleles, or combination of alleles within a gene boundary, are recoded for both the European (EUR; n = 379) and African (AFR; n = 246) populations (1000 Genomes Project; 1000genomes.org). If a SAP-containing peptide was identified in any of the proteomic datasets associated with an individual, this was indicated by a "1" in the matrix. False positives, identified by genotyping have been removed. A maximum of 1 observation of allele, or combination of alleles, occurs per gene.
(PDF)

S7 File Imputation of nsSNPs alleles in individual African and African-American datasets. Datasets resulting from application of tryptic digests were analyzed using both the Trans Proteomic Pipeline and GPM manager, as outlined in the Supplemental Methods (S1 Methods). A cohort of 5 African-American subjects, and 5 Kenyan subjects (**S5**)[39], were analyzed and peptides that contained characterized single amino acid polymorphisms were identified, collated, and summed for each individual. Peptide sequences are included with amino acid polymorphisms indicated in lower case (pept). Single nucleotide polymorphisms that account for the change in amino acid structure are represented in the table by gene name (GN), and dbSNP identifier and allele (rs#_nuc). Multiple alleles occurring within the gene boundary, either through heterozygosity or multiple SNPs are also indicated. The number of observations of alleles, or combination of alleles within a gene boundary, are recoded for both the European (EUR; n = 379) and African (AFR; n = 246) populations (1000 Genomes Project; 1000genomes.org). If a SAP-containing peptide was identified in any of the proteomic datasets associated with an individual, this was indicated by a "1" in the matrix. False positives, identified by genotyping have been removed. A maximum of 1 observation of allele, or combination of alleles, occurs per gene.
(PDF)

S1 Methods Detailed outlines of the physical and chemical treatment of hair shafts are described to allow correspondence of experimental treatments with resulting proteomic datasets. Detailed protocols for data acquisition on a Thermo Hybrid FT/LTQ, a Bruker maXis Impact qToF, and Agilent 1290/Agilent 6530 Accurate-Mass Q-ToF are outlined. A description of the discovery process of genetically variant peptides is included, particularly the creation and characterization of a custom reference protein variant database (RefSeq_Protein_Variant_Database.txt; https://zenodo.org/record/58223; DOI: 10.5281/zenodo.58223).
(DOCX)

S1 Table Admixture estimation from cohort of 60 self-identified European-Americans. Before hair samples in the European–American Cohort (EA1) were processed, DNA from each subject was evaluated for biogeographic background using the Investigative LEAD^™^ Ancestry DNA Test (Sorenson Forensics LLC, Salt Lake City, UT) that genotypes data for 190 SNPs that are ‘Ancestry Informative Markers’[38]. All subjects self-identified as European (EUR); however, some individuals were determined to have an admixture of other ancestral backgrounds; and were excluded from further treatment and analysis (subjects 00642–10, 11, 18, 22, 24, 25, 27, 34, and 43). Percent ancestry contributions (%) and standard deviations (SD) are listed for each subject.
(TIFF)

S2 Table Flanking primers for imputed nsSNP loci verification. PCR primers were designed, to flank the variant, using the Primer 3 program (Whitehead Institute for Biomedical Research). PCR reactions were carried out using the AccuPrime^™^ *Taq* DNA Polymerase System (Invitrogen^™^) following the manufacturer’s specifications. PCR product was then treated with ExoSAP-IT^®^ (Affymetrix) and subjected to Sanger Dideoxy Sequence analysis on an Applied Biosystems 3730xl 96-capillary DNA Analyzer by the DNA Sequencing Core Facility, University of Utah Health Science Cores.
(TIFF)

S3 Table Proportion of GSDMA nsSNP loci combinations in European and African populations. Individual genotypes for nsSNP loci (rs3894194, rs56030650) from the 1000 Genome Project (www.1000genomes.org, phase 1) were collated and genotype frequency (*gf*) of each combination calculated for both the European (EUR, n = 379) and African (AFR, n = 246) populations. Corresponding single amino acid polymorphisms are indicated in red.
(PDF)

S4 Table Proportion of KRT32 nsSNP loci combinations in European and African populations. Individual genotypes for nsSNP loci combinations (rs2071561, rs2071563, rs72830046) from the 1000 Genome Project (www.1000genomes.org) were collated (sum) and the genotype frequency of each combination (*gf*) calculated for the European (EUR) and African (AFR) populations. Peptides that do not have a single point of origin in the genome (eg. *ADLEAQVESLK*) are indicated by italics. Corresponding single amino acid polymorphisms are indicated in red.
(TIFF)

S5 Table Proportion of KRT35 nsSNP loci combinations in European and African populations. Individual genotypes for nsSNP loci (rs12451652, rs2071601, and rs743686) from the 1000 Genome Project (www.1000genomes.org) were collated (sum) and the genotype frequency (*gf*) of each combination was calculated for both the European (EUR) and African (AFR) populations. Corresponding single amino acid polymorphisms are indicated in red.
(TIFF)

S6 Table Proportion of KRT40 nsSNP loci combinations in European and African populations. Individual genotypes for nsSNP loci (rs2010027, rs150812789) from the 1000 Genome Project (www.1000genomes.org) were collated (sum) and the genotype frequency (*gf*) of each combination calculated for both the European (EUR) and African (AFR) population. Corresponding single amino acid polymorphisms are indicated. If two peptides are used to infer the presence of a SNP allele then both sequences are included in red.
(TIFF)

S7 Table Proportion of KRT81 nsSNP loci combinations in European and African populations. Individual genotypes for nsSNP loci (rs6580873, rs2071588, and rs79897879) from the 1000 Genome Project (www.1000genomes.org) were collated (sum) and the genotype frequency (*gf*) of each combination calculated for both the European (EUR) and African (AFR) population. Peptides that do not have a single point of origin in the genome are indicated by italics. Corresponding single amino acid polymorphisms are indicated in red.
(TIFF)

S8 Table Proportion of LRRC15 nsSNP loci combinations in European and African populations. Individual genotypes for nsSNP loci (rs13070515, and rs13060627) from the 1000 Genome Project (www.1000genomes.org) were collated (sum) and the genotype frequency (*gf*) of each combination calculated for both the European (EUR) and African (AFR) populations. Corresponding single amino acid polymorphisms are indicated in red.
(TIFF)

S9 Table Peptides containing single amino acid polymorphisms (SAPs) identified in the hair proteome. Peptides bearing single amino acid polymorphisms (SAPs) in the hair proteome are listed in order of Gene Name. The genotype count of each underlying SNP allele in the European (EUR) and African (AFR) population is indicated (1000 Genome Project, phase 1). The SAP is indicated in the peptide sequence in red, with the non-reference allele indicated in lower case (peptide sequence). Minor alleles appear above major alleles. Peptide sequences that were not unique, and could be attributed to more than one position on the genome, were not included. The corresponding non-synonymous SNP locus accession number (rs#) and imputed nsSNP allele nucleotide (nuc) are indicated.
(TIFF)

S10 Table Direct genotyping of subjects using Sanger sequencing. Validation of predicted DNA polymorphisms was executed using PCR primers designed to flank the variant (S2 Table, Primer 3 program, Whitehead Institute for Biomedical Research).
(TIFF)

S11 Table Sensitivity and positive predictive value measurements of genetically variant peptides. Peptides identified in subject datasets that contained single amino-acid polymorphisms (SAPs) (Gene Name = GN, SNP locus = rs#) were directly evaluated for the ability to impute nsSNP loci in corresponding subjects’ DNA using Sanger sequencing (Fig 1, S2 Fig, S4 Fig, and S5 Fig). The amino-acid sequence of the SAP-containing peptide is shown (peptide), with the SAP indicated in red and non-reference allele indicated as lower case. Corresponding nucleotide alleles (nuc) are listed in red with the minor allele appearing above the major allele. Genetically variant peptides are listed in order of increasing genotype frequency (gf) of the minor allele. SAP-containing peptides that occur in more than one gene product, and therefore are not unique, were excluded from the analysis. The percent sensitivity, measured as the proportion of nsSNP-loci that are correctly detected and imputed (true positive/(true positive + false negative)) is listed along with individual counts in parentheses. The ability of each genetically variant peptide to accurately impute the corresponding SNP allele, or positive predictive value (PPV; true positive/(true positive + false positive)), is calculated as a percentage. Individual counts are also shown in parentheses[49]. SAP-containing peptides are sorted based on increasing proportion of the minor allele in the European Population (1000 Genome Project, phase 1).
(TIFF)

S12 Table Mitochondrial haplotype analysis of subset of European-American cohort. Mitochondrial DNA in buffy coat DNA was isolated from a subset of European-American subjects (EA2) and HV1 and HV2 regions sequenced (S1 Methods). Mitochondrial DNA haplotypes and subclades were classified and percentage and population proportion determined relative to the Utah Population Database (Pr(mtDNA haplotype|Utah population)). Calculation of imputed nsSNP profile probabilities (Pr(imputed nsSNP-profile|EUR population)) were calculated relative to the European population as described in the Supplemental Methods (S1 Methods). The effect of binomial distribution on posterior allelic probabilities was determined and upper and lower limits (90% confidence interval) determined using parametric bootstrapping. Full Hardy-Weinberg equilibrium between gene boundaries, and full linkage-disequilibrium within them, were assumed. When independence between mitochondrial DNA haplotype and the imputed nsSNP allele profiles was assumed, the combined probability was calculated as the product of the two values.
(PDF)
