# Supplementary figures and images for: Demonstration of Protein-Based Human Identification Using the Hair Shaft Proteome
Source: PLoS One. 2016 Sep 7;11(9):e0160653. doi: 10.1371/journal.pone.0160653 (PMC5014411; doi:10.1371/journal.pone.0160653)

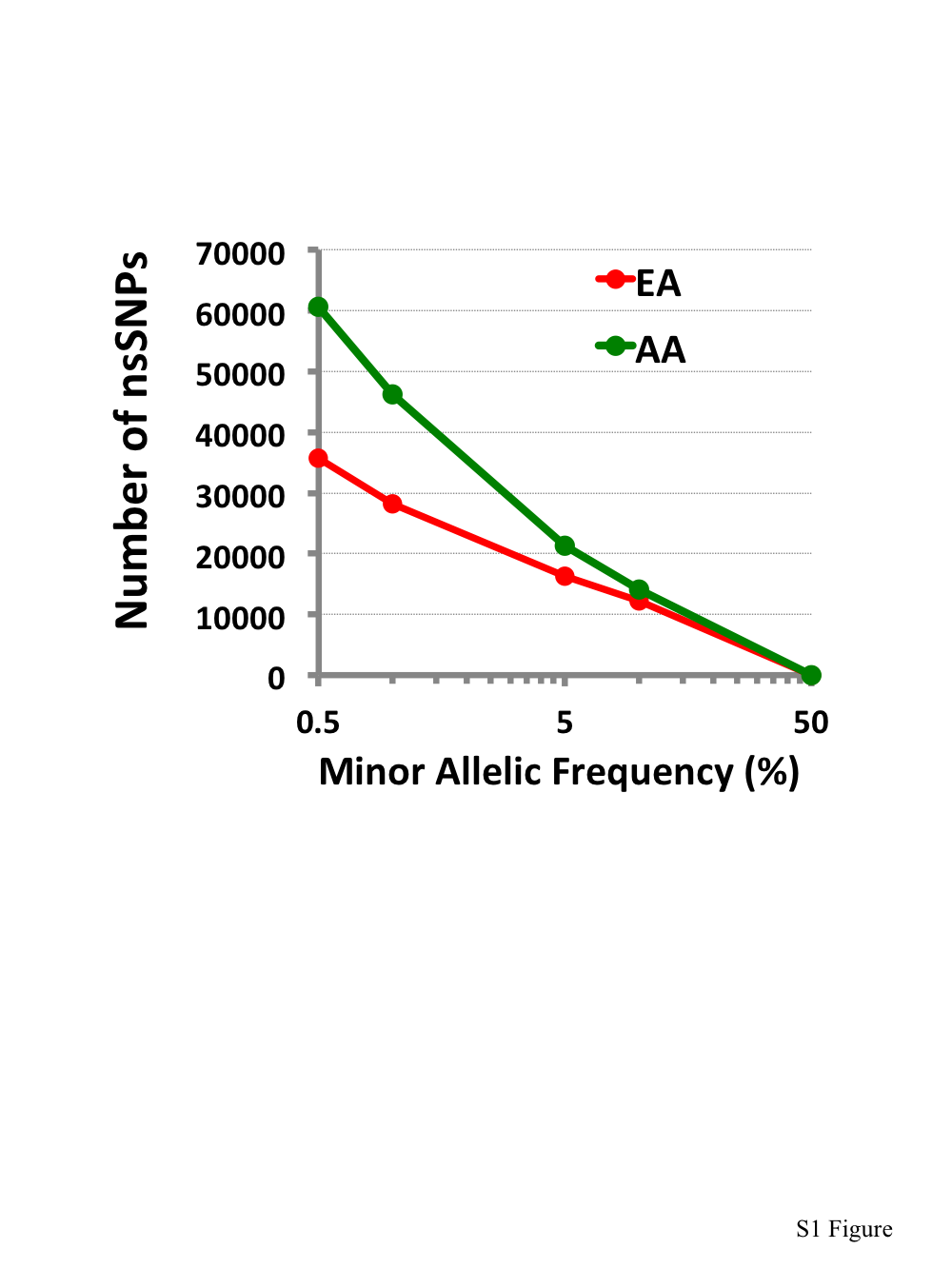

Supplement: S1 Fig — Missense SNP variants (nsSNP) were identified and counted in the NHLBI Exome Sequencing Project (ESP) database (Exome Variant Server, NHLBI GO Exome Sequencing Project, evs.gs.washington.edu/EVS/) [accessed August 1, 2013]. The Exome Variant Server contained 748,407 nsSNPs in the European–American (red) and/or African-American population (green). Counts of minor alleles (nsSNP #) at, or above, indicated frequencies (Minor Allele Frequency (%)) are plotted. (TIFF) [file pone.0160653.s001.tiff]

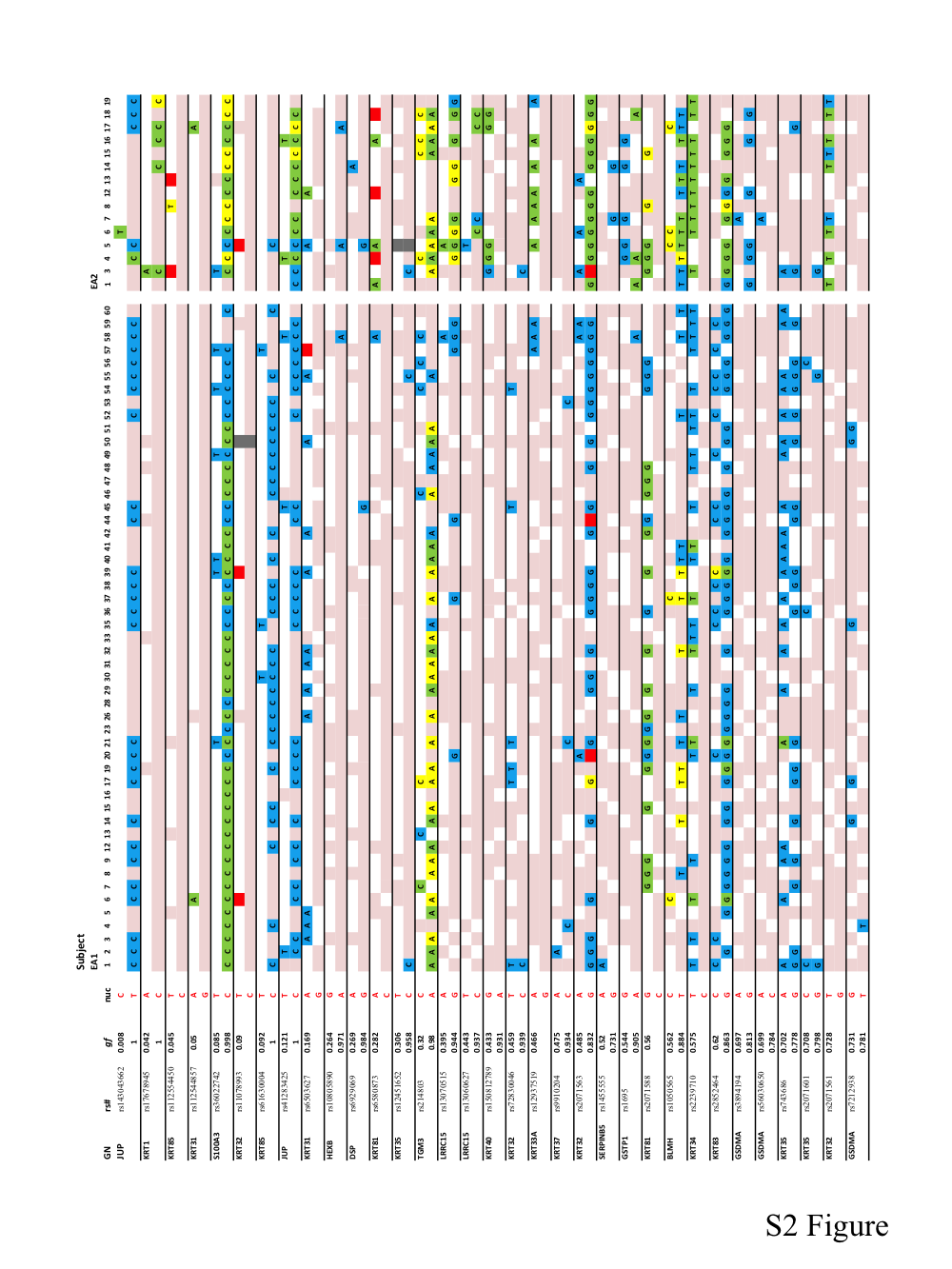

Supplement: S2 Fig — Genetically variant peptides (GVPs) that contained single amino-acid polymorphisms (SAPs) were identified in both European-American cohorts (EA1 and EA2) and directly evaluated for the ability to impute non-synonymous SNP loci in corresponding subjects’ DNA (Gene Name = GN, SNP accession number = rs#). Imputed nsSNP alleles (allele nucleotide = nuc) were directly compared to the genotype resulting from direct Sanger sequencing (S1 Methods). Correctly imputed nsSNP alleles (TP, true positives) are indicated by a colored square containing the respective nucleotide. Genetically variant peptides identified using X!Tandem and a customized database are indicated by yellow. Peptides identified using the GPM manager are indicated by blue, with redundant identifications indicated by green. False-positive identification (FP) is indicated by red squares. Alleles that were identified using Sanger sequencing, but did not contain a resulting GVP in the matching proteomic dataset (FN, false negative) are indicated by pink. Alleles absent in both subjects DNA and in resulting proteomic datasets (TN, true negatives) are indicated by white squares[49]. Failed Sanger sequencing determination of nsSNP allelic status is indicated by grey. Genetically variant peptides that could not be localized to a single genomic locus, could not be used for imputation and are not shown. Genetically variant peptides are sorted based on increasing proportion of the minor allele in the European Population (1000 Genome Project, phase 1). (TIFF) [file pone.0160653.s002.tiff]

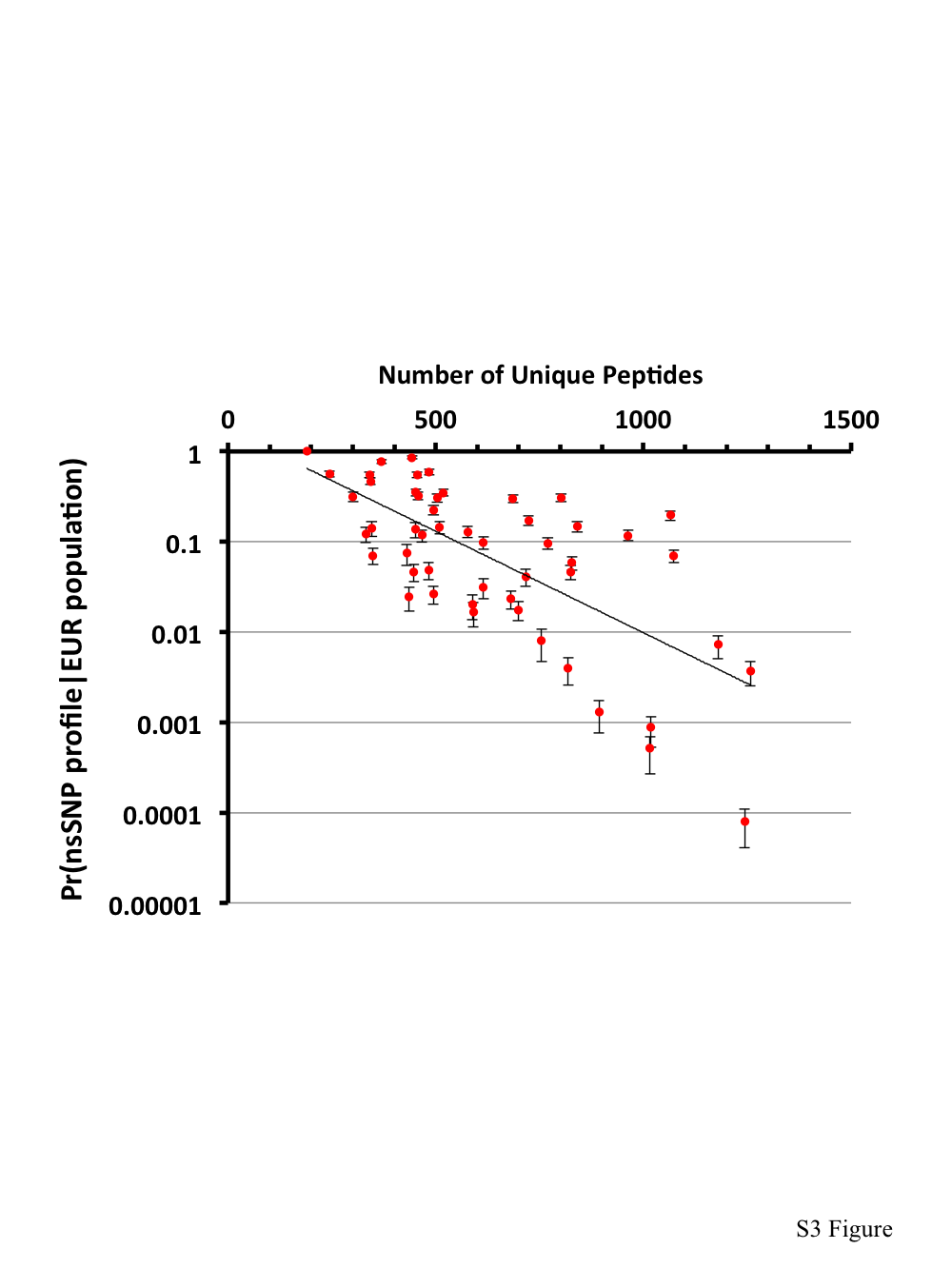

Supplement: S3 Fig — The power of discrimination, or proportion of overall imputed nsSNP profiles in the European population (Pr(imputed nsSNP profile|EUR population)), was calculated for each European-American subject (EA1, S1 Methods), and plotted against the corresponding number of unique peptides identified in the proteomic dataset (red circles). Confidence intervals (90%) were calculated using parametric bootstrapping (S1 Methods). To guide the eye, a line indicating exponential regression is also plotted (y = 1.73e−0.005x, r = 0.6811, P < 0.0001). (TIFF) [file pone.0160653.s003.tiff]

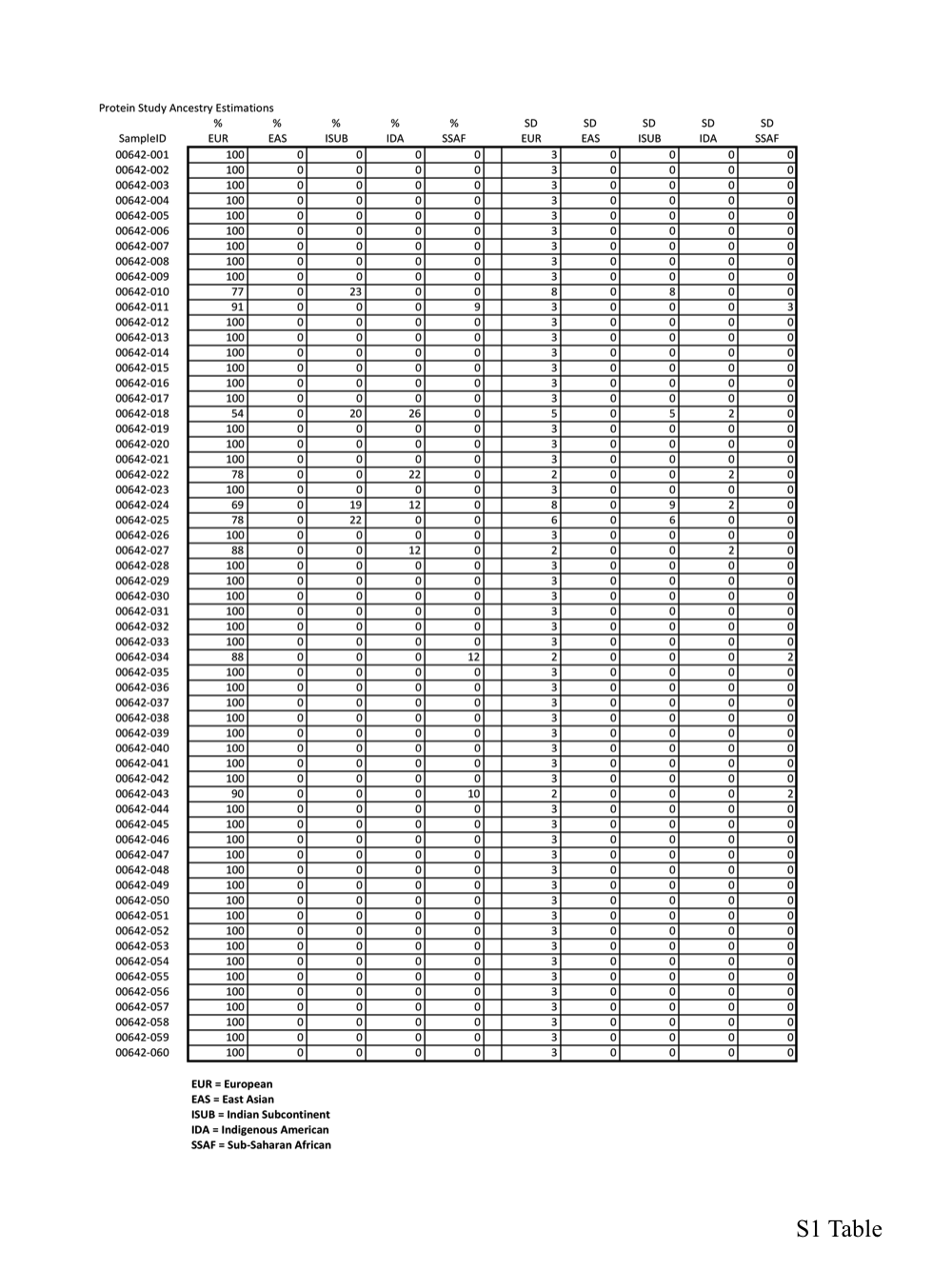

Supplement: S1 Table — Before hair samples in the European–American Cohort (EA1) were processed, DNA from each subject was evaluated for biogeographic background using the Investigative LEAD™ Ancestry DNA Test (Sorenson Forensics LLC, Salt Lake City, UT) that genotypes data for 190 SNPs that are ‘Ancestry Informative Markers’[38]. All subjects self-identified as European (EUR); however, some individuals were determined to have an admixture of other ancestral backgrounds; and were excluded from further treatment and analysis (subjects 00642–10, 11, 18, 22, 24, 25, 27, 34, and 43). Percent ancestry contributions (%) and standard deviations (SD) are listed for each subject. (TIFF) [file pone.0160653.s012.tiff]

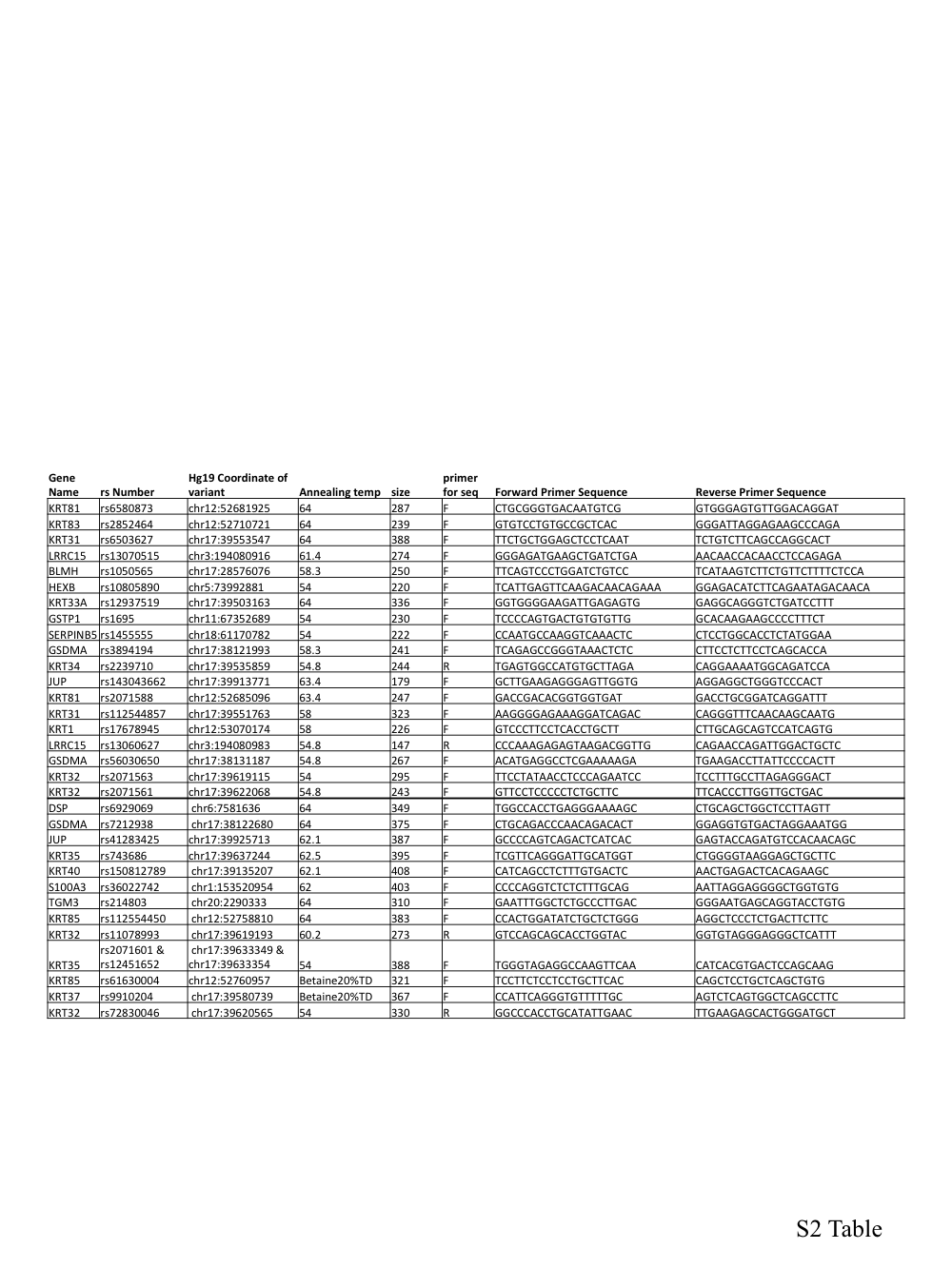

Supplement: S2 Table — PCR primers were designed, to flank the variant, using the Primer 3 program (Whitehead Institute for Biomedical Research). PCR reactions were carried out using the AccuPrime™ Taq DNA Polymerase System (Invitrogen™) following the manufacturer’s specifications. PCR product was then treated with ExoSAP-IT® (Affymetrix) and subjected to Sanger Dideoxy Sequence analysis on an Applied Biosystems 3730xl 96-capillary DNA Analyzer by the DNA Sequencing Core Facility, University of Utah Health Science Cores. (TIFF) [file pone.0160653.s013.tiff]

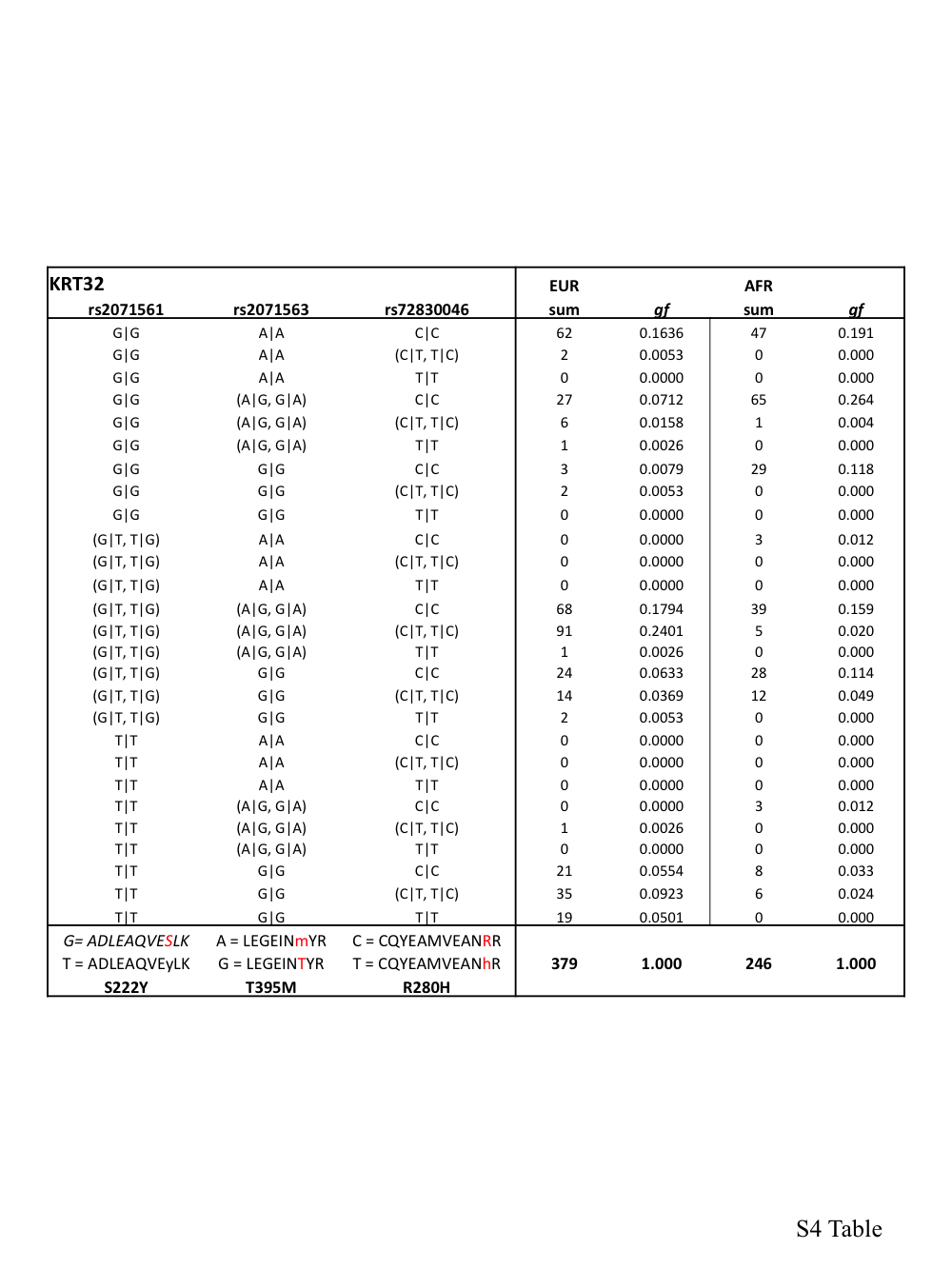

Supplement: S4 Table — Individual genotypes for nsSNP loci combinations (rs2071561, rs2071563, rs72830046) from the 1000 Genome Project (www.1000genomes.org) were collated (sum) and the genotype frequency of each combination (gf) calculated for the European (EUR) and African (AFR) populations. Peptides that do not have a single point of origin in the genome (eg. ADLEAQVESLK) are indicated by italics. Corresponding single amino acid polymorphisms are indicated in red. (TIFF) [file pone.0160653.s015.tiff]

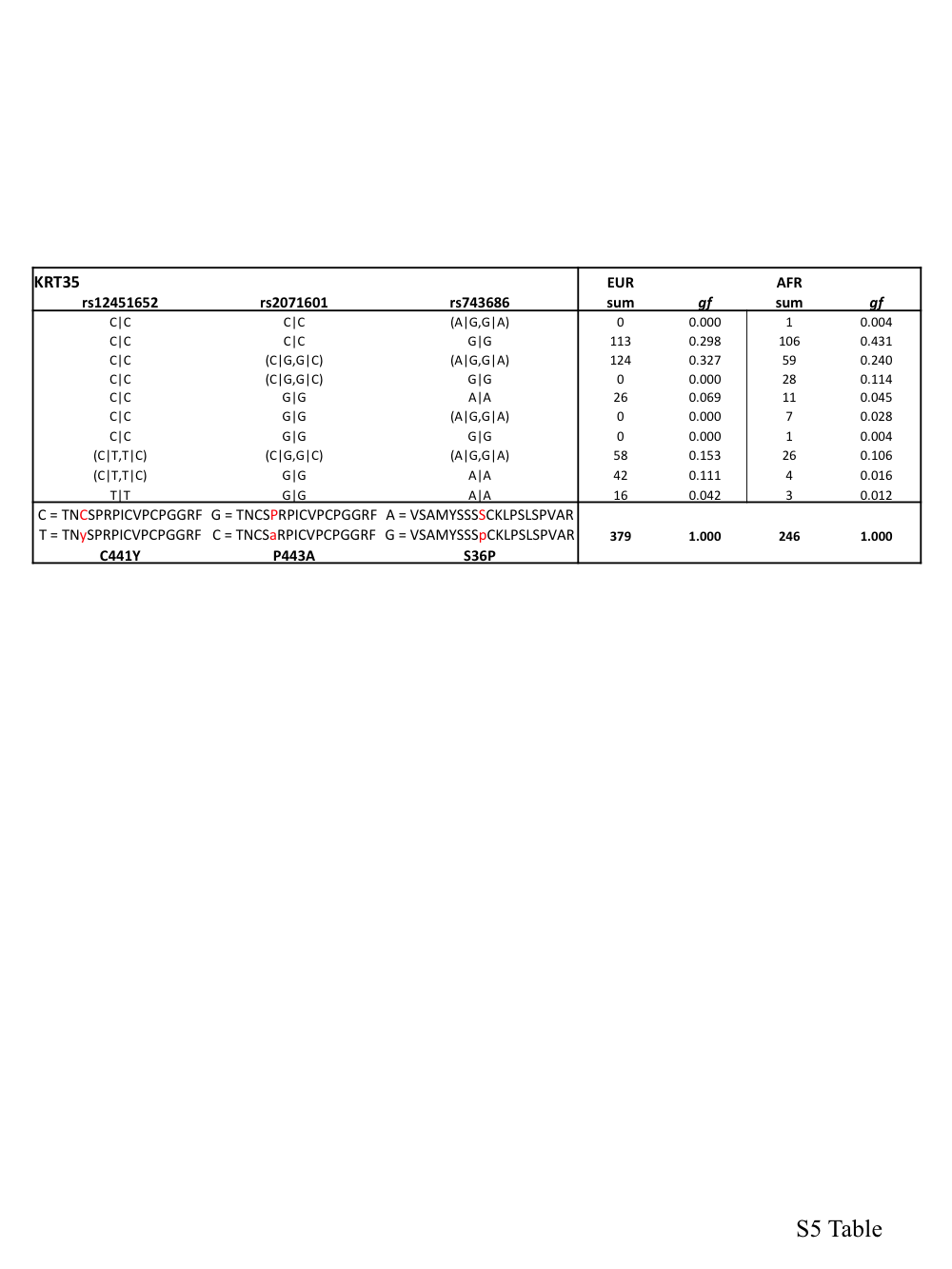

Supplement: S5 Table — Individual genotypes for nsSNP loci (rs12451652, rs2071601, and rs743686) from the 1000 Genome Project (www.1000genomes.org) were collated (sum) and the genotype frequency (gf) of each combination was calculated for both the European (EUR) and African (AFR) populations. Corresponding single amino acid polymorphisms are indicated in red. (TIFF) [file pone.0160653.s016.tiff]

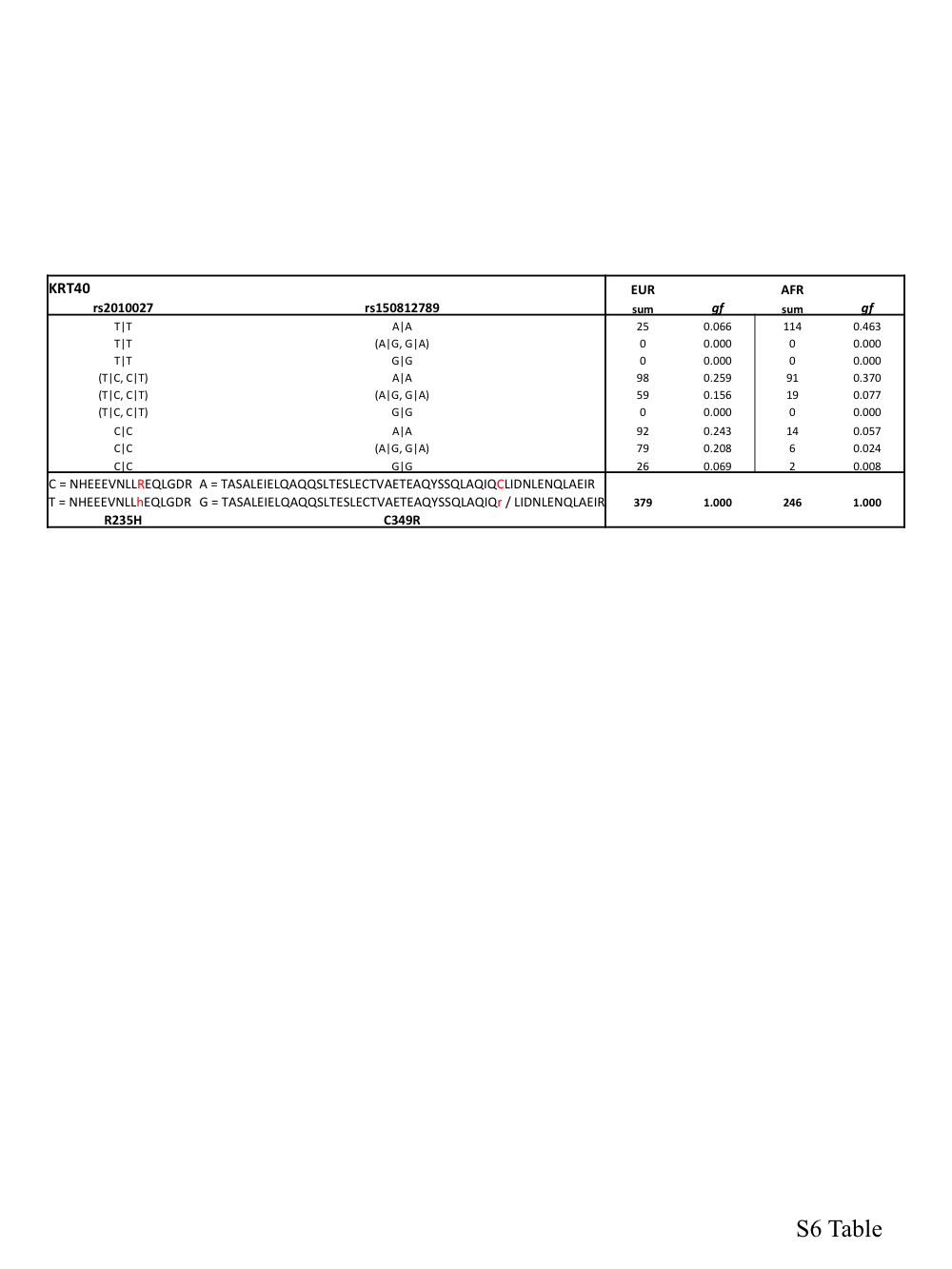

Supplement: S6 Table — Individual genotypes for nsSNP loci (rs2010027, rs150812789) from the 1000 Genome Project (www.1000genomes.org) were collated (sum) and the genotype frequency (gf) of each combination calculated for both the European (EUR) and African (AFR) population. Corresponding single amino acid polymorphisms are indicated. If two peptides are used to infer the presence of a SNP allele then both sequences are included in red. (TIFF) [file pone.0160653.s017.tiff]

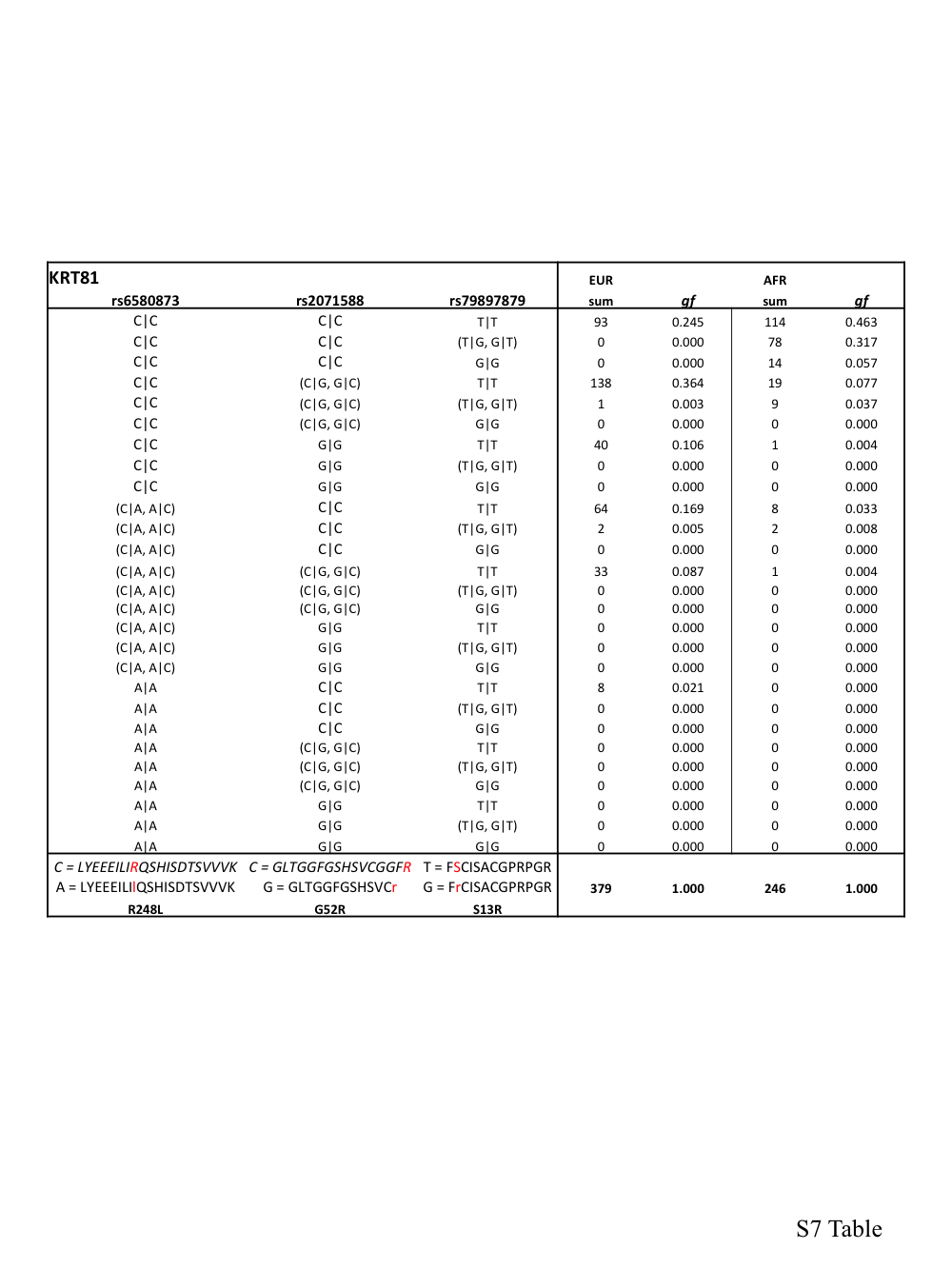

Supplement: S7 Table — Individual genotypes for nsSNP loci (rs6580873, rs2071588, and rs79897879) from the 1000 Genome Project (www.1000genomes.org) were collated (sum) and the genotype frequency (gf) of each combination calculated for both the European (EUR) and African (AFR) population. Peptides that do not have a single point of origin in the genome are indicated by italics. Corresponding single amino acid polymorphisms are indicated in red. (TIFF) [file pone.0160653.s018.tiff]

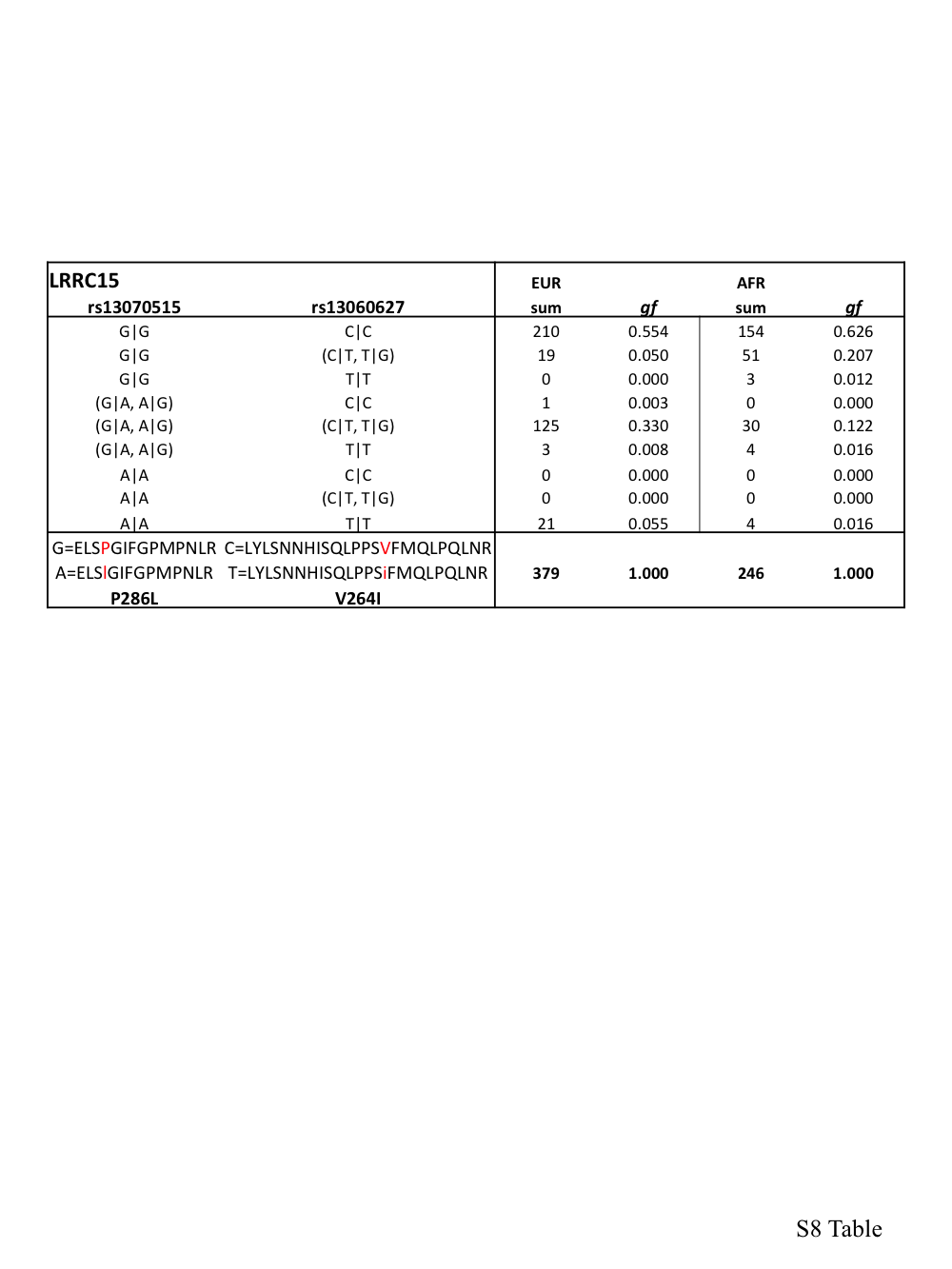

Supplement: S8 Table — Individual genotypes for nsSNP loci (rs13070515, and rs13060627) from the 1000 Genome Project (www.1000genomes.org) were collated (sum) and the genotype frequency (gf) of each combination calculated for both the European (EUR) and African (AFR) populations. Corresponding single amino acid polymorphisms are indicated in red. (TIFF) [file pone.0160653.s019.tiff]

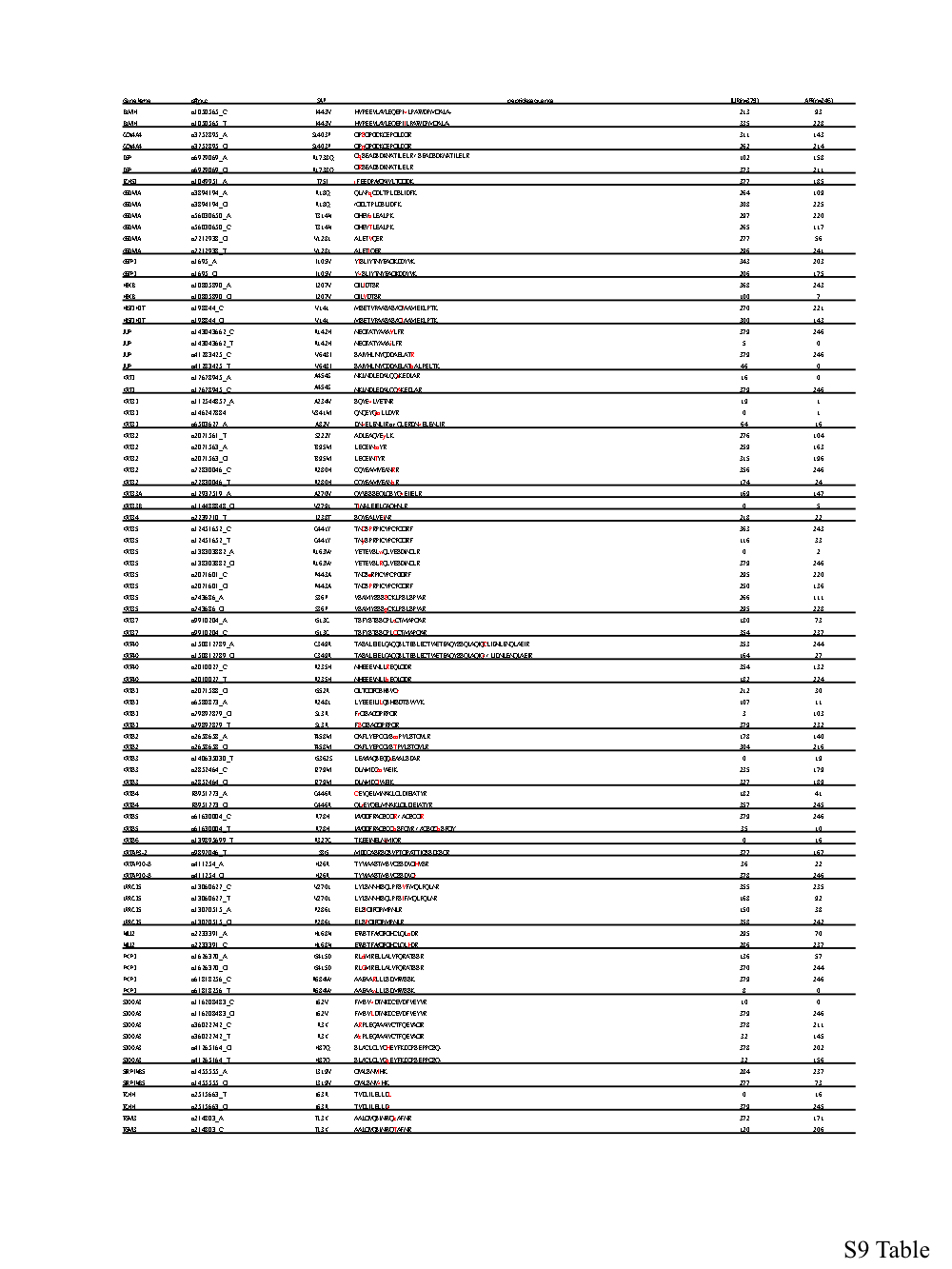

Supplement: S9 Table — Peptides bearing single amino acid polymorphisms (SAPs) in the hair proteome are listed in order of Gene Name. The genotype count of each underlying SNP allele in the European (EUR) and African (AFR) population is indicated (1000 Genome Project, phase 1). The SAP is indicated in the peptide sequence in red, with the non-reference allele indicated in lower case (peptide sequence). Minor alleles appear above major alleles. Peptide sequences that were not unique, and could be attributed to more than one position on the genome, were not included. The corresponding non-synonymous SNP locus accession number (rs#) and imputed nsSNP allele nucleotide (nuc) are indicated. (TIFF) [file pone.0160653.s020.tiff]

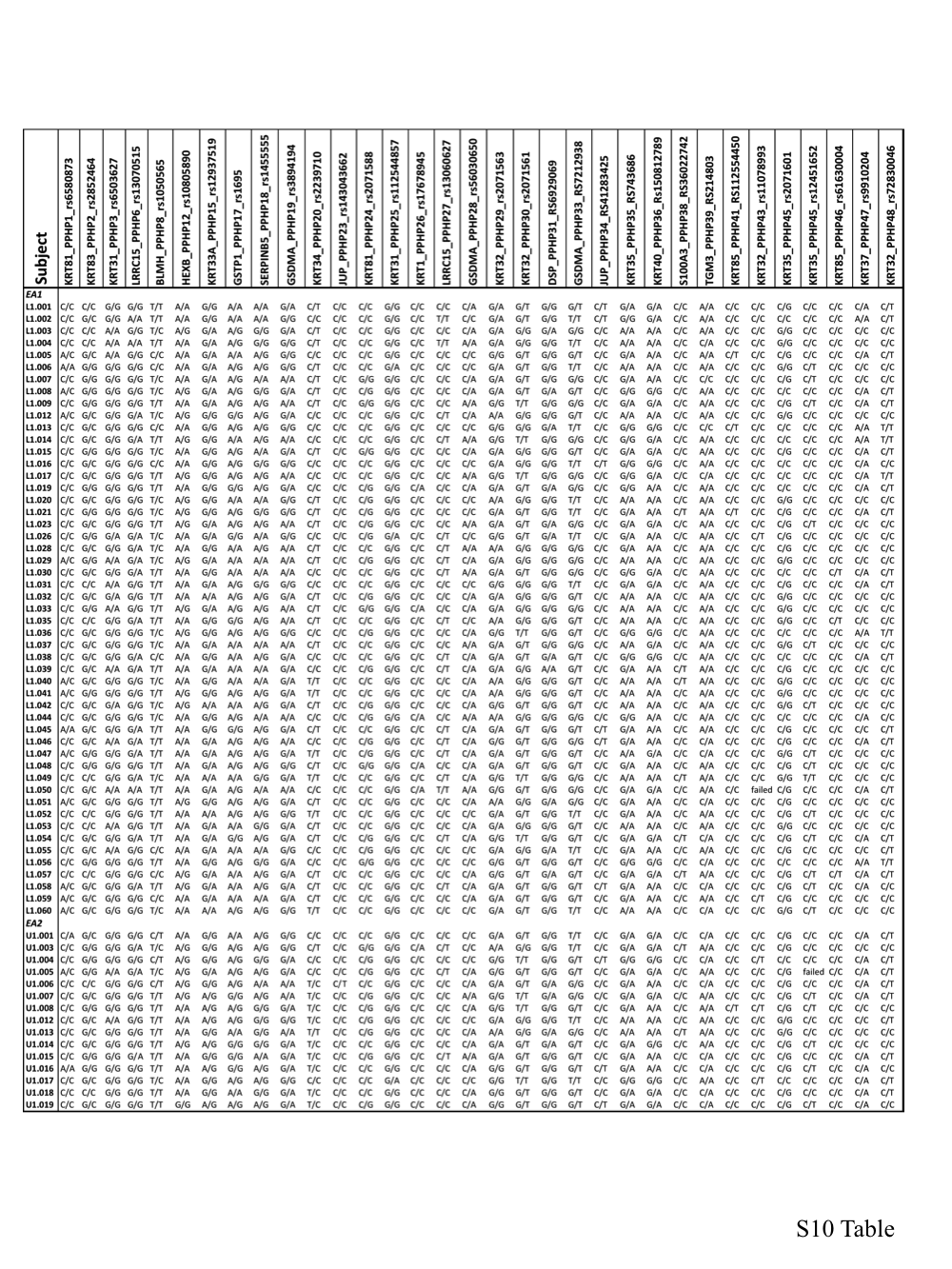

Supplement: S10 Table — Validation of predicted DNA polymorphisms was executed using PCR primers designed to flank the variant (S2 Table, Primer 3 program, Whitehead Institute for Biomedical Research). (TIFF) [file pone.0160653.s021.tiff]

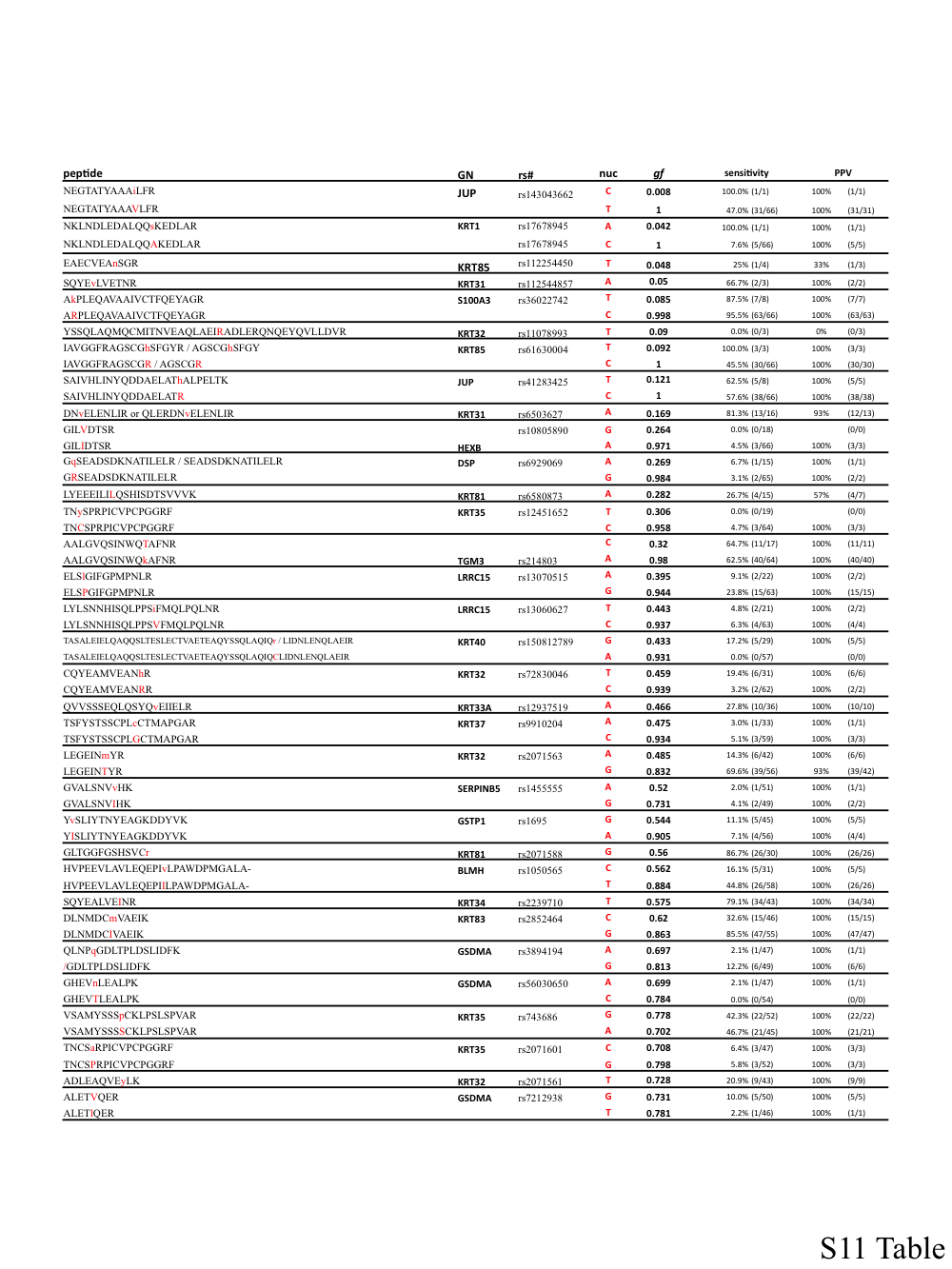

Supplement: S11 Table — Peptides identified in subject datasets that contained single amino-acid polymorphisms (SAPs) (Gene Name = GN, SNP locus = rs#) were directly evaluated for the ability to impute nsSNP loci in corresponding subjects’ DNA using Sanger sequencing (Fig 1, S2 Fig, S4 Fig, and S5 Fig). The amino-acid sequence of the SAP-containing peptide is shown (peptide), with the SAP indicated in red and non-reference allele indicated as lower case. Corresponding nucleotide alleles (nuc) are listed in red with the minor allele appearing above the major allele. Genetically variant peptides are listed in order of increasing genotype frequency (gf) of the minor allele. SAP-containing peptides that occur in more than one gene product, and therefore are not unique, were excluded from the analysis. The percent sensitivity, measured as the proportion of nsSNP-loci that are correctly detected and imputed (true positive/(true positive + false negative)) is listed along with individual counts in parentheses. The ability of each genetically variant peptide to accurately impute the corresponding SNP allele, or positive predictive value (PPV; true positive/(true positive + false positive)), is calculated as a percentage. Individual counts are also shown in parentheses[49]. SAP-containing peptides are sorted based on increasing proportion of the minor allele in the European Population (1000 Genome Project, phase 1). (TIFF) [file pone.0160653.s022.tiff]
