# Supplementary material for: Demonstration of Protein-Based Human Identification Using the Hair Shaft Proteome
Source: PLoS One. 2016 Sep 7;11(9):e0160653. doi: 10.1371/journal.pone.0160653 (PMC5014411; doi:10.1371/journal.pone.0160653)
Supplement: S1 File — Mass spectrometry datafiles in either MzML or Mascot Generic Format (mgf) were submitted to the Global Proteome Machine (www.thegpm.org) for peptide spectra matching using the X!Tandem algorithm (X! Tandem Piledriver (2015.04.01.1)). Default search parameters were used including use of the GRCh38 (ENSEMBL) male reference protein database, complete carbamidomethylation of cysteine (C+57), and potential modification of asparigine (N) and glutamine (Q) residues by deamidation (N+1, Q+1) and methionine (M+16) by oxidation. Non-default parameters that were used include the use of the point mutation (sAPS) function and inactivation of the anonymous function. Processed data files in XML format are anonymously accessible using the Global Proteome Machine accession numbers (GPM#) provided. (PDF) [file pone.0160653.s004.pdf]

Spreadsheet S6 Paralogy Analysis of SAP-containing Peptides in the Hair Proteome

	GN	Frequency EUR	AFR	nuc	rs#	Chr	SAP	peptide	Comment on tblastn analysis
	**BLMH**	0.562	0.378	T	**rs1050565**	**17**	I443V	HVPEEVLAVLEQEPIvLPAWDPMGALA-	Only one significant hit in the human genome reference assembly: to BLMH on chromosome 17. The indicated polymorphism is present in the transcript database, and is therefore a reliable SAP. No evidence of false polymorphism due to paralogy.
		0.884	0.927	A	**rs1050565**	**17**	I443V	HVPEEVLAVLEQEPIILPAWDPMGALA-	Only one significant hit in the human genome reference assembly: to BLMH on chromosome 17. The indicated polymorphism is present in the transcript database, and is therefore a reliable SAP. No evidence of false polymorphism due to paralogy.
	**COL4A4**	0.691	0.87	G	**rs3752895**	**2**	S1403P	GPpGPGCKGEPGLDGR	One match to COL4A4 transcript variant X5, no significant similarlty in genome search (possibly intron disruption)
		0.821	0.581	A	**rs3752895**	**2**	S1403P	GPSGPGCKGEPGLDGR	One match to COL4A4 transcript variant X5, no significant similarlty in genome search (possibly intron disruption)
	**DSP**	0.269	0.642	A	**rs6929069**	**6**	R1738Q	GqSEADSDKNATILELR	Only one significant hit in the human genome reference assembly: to DSP on chromosome 6. The indicated polymorphism is not present in the transcript database but the SAP is likely to be reliable. No evidence of false polymorphism due to paralogy.
		0.984	0.858	G	**rs6929069**	**6**	R1738Q	GRSEADSDKNATILELR	Only one significant hit in the human genome reference assembly: to DSP on chromosome 6. The indicated polymorphism is not present in the transcript database but the SAP is likely to be reliable. No evidence of false polymorphism due to paralogy.
	**GSDMA**	0.491	0.642	C	**rs3894194**	**17**	R18Q	GDLTPLDSLIDFK	Only one significant hit in the human genome reference assembly: to GSDMA on chromosome 17. Also detected in mRNAs but with no variants detected. No evidence of false polymorphism due to paralogy.
		0.812	0.915	T	**rs3894194**	**17**	R18Q	QLNPqGDLTPLDSLIDFK	Only one significant hit in the human genome reference assembly: to GSDMA on chromosome 17. Also detected in mRNAs but with no variants detected. No evidence of false polymorphism due to paralogy.
	**GSDMA**	0.699	0.476	A	**rs56030650**	**17**	T314N	GHEVnLEALPK	No signficant similarity detected with a tblastn search; sequence too short.
		0.784	0.894	C	**rs56030650**	**17**	T314N	GHEVTLEALPK	No signficant similarity detected with a tblastn search; sequence too short.
	**GSDMA**	0.731	0.227	G	**rs7212938**	**17**	V128L	ALETVQER	No signficant similarity detected with a tblastn search; sequence too short.
		0.781	0.979	T	**rs7212938**	**17**	V128L	ALETlQER	No signficant similarity detected with a tblastn search; sequence too short.
	**GSTP1**	0.544	0.712	G	**rs1695**	**11**	I105V	YvSLIYTNYEAGKDDYVK	One match to GSTP1 mRNAs, including the indicated polymorphism, no significant similarlty in genome search (possibly intron disruption)
		0.905	0.826	A	**rs1695**	**11**	I105V	YISLIYTNYEAGKDDYVK	One match to GSTP1 mRNAs, including the indicated polymorphism, no significant similarlty in genome search (possibly intron disruption)
	**HEX B**	0.264	0.028	G	**rs10805890**	**5**	I207V	GILVDTSR	No signficant similarity detected with a tblastn search; sequence too short.
		0.971	1	A	**rs10805890**	**5**	I207V	GILIDTSR	No signficant similarity detected with a tblastn search; sequence too short.
	**HIST1H1T**	0.712	0.899	C	**rs198844**	**6**	V14L	MSETVPAASASAGVAAMEKLPTK	Histone H1t, other signficant matches had several amino acid diffrences. No evidence of false polymoprhism due to paralogy.
		0.792	0.582	G	**rs198844**	**6**	V14L	MSETVPAASASAGlAAMEKLPTK	Histone H1t, other signficant matches had several amino acid diffrences. No evidence of false polymoprhism due to paralogy.
	**JUP**	0.121	0.02	T	**rs41283425**	**17**	R142H	SAIVHLINYQDDAELAThALPELTK	Two matches, to JUP on chromosome 17 and CTNNB1 on chromosome 3. There are several fixed variants that confirm the peptide undoubtedly belongs to JUP. Therefore there is no evidence of false polymorphism due to paralogy.
		1	1	C	**rs41283425**	**17**	R142H	SAIVHLINYQDDAELATR	Two matches, to JUP on chromosome 17 and CTNNB1 on chromosome 3. There are several fixed variants that confirm the peptide undoubtedly belongs to JUP. Therefore there is no evidence of false polymorphism due to paralogy.
	**JUP**	0.013	0	T	**rs143043662**	**17**	V648I	NEGTATYAAAiLFR	One match to JUP in mRNAS. No signficiant similarity in genome search, possibly due to intron disruption. No evidence of false polymorphism due to paralogy.
		1	1	C	**rs143043662**	**17**	V648I	NEGTATYAAAVLFR	One match to JUP in mRNAS. No signficiant similarity in genome search, possibly due to intron disruption. No evidence of false polymorphism due to paralogy.
	**KRT1**	0.042	0	A	**rs17678945**	**12**	A454S	NKLNDLEDALQQsKEDLAR	Two matches to tandem copies of KRT1 and KRT2. KRT2, however, contains a variant amino acid residue within this peptide that confirms the peptide belongs to KRT1. Therefore, there is no evidence of a false polymorphism due to paralogy. All mRNA sequences contain the ancestral variant.
	**KRT31**	0.169	0.065	A	**rs6503627**	**17**	A82V	DNvELENLIR	No signficant similarity detected with a tblastn search; sequence too short.
	**KRT31**	0.05	0.004	A	**rs112544857**	**17**	A234V	SQYEvLVETNR	No signficant similarity detected with a tblastn search; sequence too short.
	**KRT31**	0	0.004	T	**rs146247884**	**17**	V341M	QNQEYQmLLDVR	No signficant similarity detected with a tblastn search.
	**KRT32**	0.748	0.493	T	**rs2071561**	**17**	S222Y	ADLEAQVEyLK	No signficant similarity detected with a tblastn search; sequence too short.
	**KRT32**	0.682	0.663	A	**rs2071563**	**17**	T395M	LEGEINmYR	No signficant similarity detected with a tblastn search; sequence too short.
		0.832	0.796	G	**rs2071563**	**17**	T395M	LEGEINTYR	No signficant similarity detected with a tblastn search; sequence too short.
	**KRT32**	0.459	0.098	T	**rs72830046**	**17**	R280H	CQYEAMVEANhR	One match to KRT32 with no detection of polymorphism in mRNAs. No evidence of false polymoprphism due to paralogy.
		0.939	1	C	**rs72830046**	**17**	R280H	CQYEAMVEANRR	One match to KRT32 with no detection of polymorphism in mRNAs. No evidence of false polymoprphism due to paralogy.
	**KRT33A**	0.446	0.597	A	**rs12937519**	**17**	A270V	QVVSSSEQLQSYQvEIIELR	Multiple matches in genome search, all withing the KRT gene family on chromosome 17, including pseudogenes, with no distinguishing variants elsewhere in the sequence. The mRNA search, however, revealed two mRNAs with the derived A270V variant, both from KRT33A, so the polymorphism is probably in this gene.
*	**KRT33B**	0	0.02	G	**rs114488848**	**17**	V279L	TlNALEIELQAQHNLR	Multiple matches in genome and mRNA search to KRT gene family on chromosome 17, including pseudogenes. None of the mRNAs contained the I238T variant, so the gene to which this variant belongs cannot be conclusively identified by this method. There is a possibility of a false polymophism due to paralogy.
	**KRT34**	0.575	0.089	A	**rs2239710**	**17**	I238T	SQYEALVEINR	No signficant similarity detected with a tblastn search; sequence too short.
	**KRT35**	0.702	0.451	A	**rs743686**	**17**	S36P	VSAMYSSSSCKLPSLSPVAR	One match to KRT35 in genomic assembly and in mRNA sequences. S36P variant not detected in any sequence. No evidence of false polymorphism due to paralogous sequences.
		0.778	0.927	G	**rs743686**	**17**	S36P	VSAMYSSSpCKLPSLSPVAR	One match to KRT35 in genomic assembly and in mRNA sequences. S36P variant not detected in any sequence. No evidence of false polymorphism due to paralogous sequences.
	**KRT35**	0.708	0.623	C	**rs2071601**	**17**	P443A	TNCSaRPICVPCPGGRF	One match to KRT35 in genomic assembly and in mRNA sequences. P443A variant detected in mRNA sequences. No evidence of false polymorphism due to paralogous sequences.
		0.798	0.859	G	**rs2071601**	**17**	P443A	TNCSPRPICVPCPGGRF	One match to KRT35 in genomic assembly and in mRNA sequences. P443A variant detected in mRNA sequences. No evidence of false polymorphism due to paralogous sequences.
	**KRT35**	0.306	0.134	T	**rs12451652**	**17**	C441Y	TNySPRPICVPCPGGRF	One match to KRT35 in genomic assembly and in mRNA sequences. C441 variant not detected in mRNA sequences. No evidence of false polymorphism due to paralogous sequences. There is a common variant at N440T present in several of the mRNA sequences.
		0.958	0.988	C	**rs12451652**	**17**	C441Y	TNCSPRPICVPCPGGRF	One match to KRT35 in genomic assembly and in mRNA sequences. C441 variant not detected in mRNA sequences. No evidence of false polymorphism due to paralogous sequences. There is a common variant at N440T present in several of the mRNA sequences.
	**KRT35**	0	0.008		**rs138303882**	**17**	R163W	YETEVSLwQLVESDINGLR	Multiple matches in genome search, all withing the KRT gene family on chromosome 17, including pseudogenes, but with at least one distinguishing variant. Best match is KRT35. No evidence of R163W variant in mRNA. No evidence of false polymorphism due to paralogy.
		**1**	**1**		**rs138303882**	**17**	R163W	YETEVSLRQLVESDINGLR	Multiple matches in genome search, all withing the KRT gene family on chromosome 17, including pseudogenes, but with at least one distinguishing variant. Best match is KRT35. No evidence of R163W variant in mRNA. No evidence of false polymorphism due to paralogy.
	**KRT37**	0.475	0.297	A	**rs9910204**	**17**	G13C	TSFYSTSSCPLcCTMAPGAR	One match to KRT37 in both genomic and mRNA databases. No evidence of G13C variant in mRNAs. No evidence of false polymorphism due to paralogy.
		0.934	0.963	C	**rs9910204**	**17**	G13C	TSFYSTSSCPLGCTMAPGAR	One match to KRT37 in both genomic and mRNA databases. No evidence of G13C variant in mRNAs. No evidence of false polymorphism due to paralogy.
	**KRT39**	0	0.02	T	**rs142154718**	**17**	S86N	FSLDDCnWYGEGINSNEK	One match to KRT39 in both genomic and mRNA databases. No evidence of S86N variant in mRNAs. No evidence of false polymorphism due to paralogy.
		1	1	C	**rs142154718**	**17**	S86N	FSLDDCSWYGEGINSNEK	One match to KRT39 in both genomic and mRNA databases. No evidence of S86N variant in mRNAs. No evidence of false polymorphism due to paralogy.
	**KRT40**	0.433	0.11	G	**rs150812789**	**17**	C349R	TASALEIELQAQQSLTESLECTVAETEAQYSSQLAQIQrLIDNLENQLAEIR	One match to KRT40 in both genomic and mRNA databases. C349R variant detected in mRNAs. No evidence of false polymorphism due to paralogy.
		0.931	0.992	A	**rs150812789**	**17**	C349R	TASALEIELQAQQSLTESLECTVAETEAQYSSQLAQIQCLIDNLENQLAEIR	One match to KRT40 in both genomic and mRNA databases. C349R variant detected in mRNAs. No evidence of false polymorphism due to paralogy.
	**KRT40**	0.647	0.925	C	**rs2010027**	**17**	R235H	NHEEEVNLLREQLGDR	One match to KRT40 in both genomic and mRNA databases, albeit with two variants in the genomic database. The protein database, however, shows a complete 100% match. No evidence of false polymorphism due to paralogy.
		0.816	0.474	T	**rs2010027**	**17**	R235H	NHEEEVNLLhEQLGDR	One match to KRT40 in both genomic and mRNA databases, albeit with two variants in the genomic database. The protein database, however, shows a complete 100% match. No evidence of false polymorphism due to paralogy.
	**KRT81**	0.282	0.045	A	**rs6580873**	**12**	R248L	LYEEEILILQSHISDTSVVVK	One match to KRT81 in both genomic and mRNA databases, albeit with several variants in the genomic database. The protein and mRNA databases, however, show 100% identity across the entire sequence. No evidence of false polymorphism due to paralogy.
		0.56	0.122	C	**rs2071588**	**12**	G52R	GLTGGFGSHSVCr	100% match to mRNA incorrectly identified as KRT86; according to my own analysis,it's a better match to KRT81, including the G52R variant in the mRNA database (emb|Y19206.1). No evidence of false polymorphism due to paralogy.
	**KRT81**	0.008	0.419	G	**rs79897879**	**12**	S13R	FrCISACGPRPGR	One match to KRT81 in both genomic and mRNA databases. All mRNAs have the ancestral sequence. No evidence of false polymorphism due to false paralogy.
		1	0.943	T	**rs79897879**	**12**	S13R	FSCISACGPRPGR	One match to KRT81 in both genomic and mRNA databases. All mRNAs have the ancestral sequence. No evidence of false polymorphism due to false paralogy.
	**KRT82**	0.734	0.569	G	**rs2658658**	**12**	T458M	GAFLYEPCGVSTPVLSTGVLR	One match to KRT82 in both genomic and mRNA databases. All mRNAs have the ancestral sequence. No evidence of false polymorphism due to paralogy.
		0.797	0.878	A	**rs2658658**	**12**	T458M	GAFLYEPCGVSmPVLSTGVLR	One match to KRT82 in both genomic and mRNA databases. All mRNAs have the ancestral sequence. No evidence of false polymorphism due to paralogy.
	**KRT83**	0.863	0.768	C	**rs2852464**	**12**	I279M	DLNMDCIVAEIK	No signficant similarity detected with a tblastn search.
		0.62	0.728	G	**rs2852464**	**12**	I279M	DLNMDCmVAEIK	No signficant similarity detected with a tblastn search.
*	**KRT83**	0	0.077	T	**rs140635030**	**12**	G362S	LEAAVAQSEQQsEAALSDAR	Multiple matches in genome and mRNA search to KRT gene family on chromosome 12, including pseudogenes. None of the mRNAs contained the G362S variant, so the gene to which this variant belongs cannot be conclusively identified by this method. There is a possibility of a false polymorphism due to paralogy.
	**KRT84**	0.48	0.167	A	**rs951773**	**12**	C446R	CEYQELMNAKLGLDIEIATYR	One match to KRT84. No evidence of false polymorphism due to paralogy.
		0.942	0.996	G	**rs951773**	**12**	C446R	rEYQELMNAKLGLDIEIATYR	One match to KRT84. No evidence of false polymorphism due to paralogy.
	**KRT85**	0.092	0.041	A	**rs61630004**	**12**	R78H	IAVGGFRAGSCGhSFGYR	One match to KRT85, R78H polymorphism not detected. No evidence of false polymorphism due to paralogy.
		1	1	G	**rs61630004**	**12**	R78H	IAVGGFRAGSCGR	One match to KRT85, R78H polymorphism not detected. No evidence of false polymorphism due to paralogy.
*	**KRT86**	0	0.065	T	**rs139895699**	**12**	R327C	TKEEINELNcMIQR	Best match is KRT81 and some KRT pseudogenes. Uncertain. Possible false polymorphism.
	**KRTAP10-8**	0.095	0.089	A	**rs411254**	**21**	H26R	TYVIAASTMSVCSSDVGHVSR	One match to KRTAP10-8. H26R variant is present in the mRNA database. No evidence of false polymorphism due to paralogy.
		0.997	1	G	**rs411254**	**21**	H26R	TYVIAASTMSVCSSDVGr	One match to KRTAP10-8. H26R variant is present in the mRNA database. No evidence of false polymorphism due to paralogy.
	**LRRC15**	0.395	0.154	A	**rs13070515**	**3**	P286L	ELSlGIFGPMPNLR	One match to LRRC15. P286l polymorphism is in the mRNA database. No evidence of false polymorphism.
		0.944	0.984	G	**rs13070515**	**3**	P286L	ELSPGIFGPMPNLR	One match to LRRC15. P286l polymorphism is in the mRNA database. No evidence of false polymorphism.
	**LRRC15**	0.443	0.374	T	**rs13060627**	**3**	V270L	LYLSNNHISQLPPSlFMQLPQLNR	One match to LRRC15. V270L polymorphism not detected in the mRNA database. No evidence of false polymorphism.
		0.937	0.955	C	**rs13060627**	**3**	V270L	LYLSNNHISQLPPSVFMQLPQLNR	One match to LRRC15. V270L polymorphism not detected in the mRNA database. No evidence of false polymorphism.
	**NEU2**	0.755	0.973	C	**rs2233391**	**2**	H168N	EWSTFAVGPGHCLQLHDR	One match to NEU2. H168N polymorphism is present in the mRNA database. No evidence of false polymorphism.
		0.778	0.285	A	**rs2233391**	**2**	H168N	EWSTFAVGPGHCLQLnDR	One match to NEU2. H168N polymorphism is present in the mRNA database. No evidence of false polymorphism.
	**PKP1**	0.021	0	T	**rs61818256**	**1**	R684W	AAEAAwLLLSDMWSSK	One match to PKP1. R684W polymorphism is not detected in the mRNA database. No evidence of false polymorphism.
		1	1	C	**rs61818256**	**1**	R684W	AAEAARLLLSDMWSSK	One match to PKP1. R684W polymorphism is not detected in the mRNA database. No evidence of false polymorphism.
	**S100A3**	0.085	0.589	T	**rs36022742**	**1**	R3K	AkPLEQAVAAIVCTFQEYAGR	One match to S100A3. R3K polymorphism is not detected in the mRNA database. No evidence of false polymorphism.
		0.998	0.858	C	**rs36022742**	**1**	R3K	ARPLEQAVAAIVCTFQEYAGR	One match to S100A3. R3K polymorphism is not detected in the mRNA database. No evidence of false polymorphism.
	**SERPINB5**	0.52	0.74	C	**rs1455555**	**18**	I319V	GVALSNVvHK	No signficant similarity detected with a tblastn search.
		0.731	0.297	T	**rs1455555**	**18**	I319V	GVALSNVIHK	No signficant similarity detected with a tblastn search.
	**TGM3**	0.317	0.696	G	**rs214803**	**20**	T13K	AALGVQSINWQTAFNR	One match to TGM3.T13K polymorphism is present in the mRNA database. No evidence of false polymorphism.
		0.982	0.838	T	**rs214803**	**20**	T13K	AALGVQSINWQkAFNR	One match to TGM3.T13K polymorphism is present in the mRNA database. No evidence of false polymorphism.
