# Supplementary material for: Demonstration of Protein-Based Human Identification Using the Hair Shaft Proteome
Source: PLoS One. 2016 Sep 7;11(9):e0160653. doi: 10.1371/journal.pone.0160653 (PMC5014411; doi:10.1371/journal.pone.0160653)
Supplement: S2 File — Peptides that occur in more than one gene product cannot be used for imputation. Every peptide therefore was analyzed by submission to the PROWL website for protein information (http://prowl.rockefeller.edu/prowl/proteininfo.html) and searched against the IPI human database. Only peptides with a match to a single gene product, or no matches, were accepted as unique. Additional scrutiny, specifically the elimination of the possibility of false polymorphism due to paralogy, was conducted by submitting each sequence to a tblastn search (http://blast.ncbi.nlm.nih.gov/Blast.cgi) and analyzing the resulting sequence alignments. In the event that a tblastn search did not conclusively eliminate the possibility of false paralogy (as is the case with rs114488848, rs140635030, rs139895699) then each wild type peptide sequence was submitted to the PROWL database, number of gene products containing the sequence identified and the presence, or absence, of each polymorphism examined using the ESP exomic database (http://evs.gs.washington.edu/EVS/). In each case where we could not conclusively eliminate false polymorphism, there was only one gene product containing the polymorphism. However, at this stage we cannot formally exclude the possibility that some polymorphisms may also exist in pseudogenes. (PDF) [file pone.0160653.s005.pdf]

rank	UNIPROT #	protein name
1	KRT86_HUMAN	Keratin, type II cuticular Hb6
2	KRT85_HUMAN	Keratin, type II cuticular Hb5
3	K1H1_HUMAN	Keratin, type I cuticular Ha1
4	KT33B_HUMAN	Keratin, type I cuticular Ha3-II
5	KT33A_HUMAN	Keratin, type I cuticular Ha3-I
6	KRT83_HUMAN	Keratin, type II cuticular Hb3
7	KRT34_HUMAN	Keratin, type I cuticular Ha4
8	TRY1_BOVIN	Pancreatic Trypsin
9	KRT81_HUMAN	Keratin, type II cuticular Hb1
10	KRT82_HUMAN	Keratin, type II cuticular Hb2
11	KRA31_HUMAN	Keratin-associated protein 3-1
12	KR111_HUMAN	Keratin-associated protein 11-1
13	KRA32_HUMAN	Keratin-associated protein 3-2
14	DESP_HUMAN	Desmoplakin
15	ICAM2_HUMAN	Intercellular adhesion molecule 2
16	VSIG8_HUMAN	V-set and immunoglobulin domain-containing protein 8
17	K1H2_HUMAN	Keratin, type I cuticular Ha2
18	S10A3_HUMAN	Protein S100-A3
19	KRT35_HUMAN	Keratin, type I cuticular Ha5
20	DSG4_HUMAN	Desmoglein-4
21	PKP1_HUMAN	Plakophilin-1
22	SBP1_HUMAN	Selenium-binding protein 1
23	KRA43_HUMAN	Keratin-associated protein 4-3
24	KRA33_HUMAN	Keratin-associated protein 3-3
25	K1C39_HUMAN	Keratin, type I cytoskeletal 39
26	CALL3_HUMAN	Calmodulin-like protein 3
27	KRA92_HUMAN	Keratin-associated protein 9-2
28	TGM3_HUMAN	Protein-glutamine gamma-glutamyltransferase E
29	KR132_HUMAN	Keratin-associated protein 13-2
30	TRI29_HUMAN	Tripartite motif-containing protein 29
31	KR261_HUMAN	Keratin-associated protein 26-1
32	1433S_HUMAN	14-3-3 protein sigma
33	LEG3_HUMAN	Galectin-3
34	KRT38_HUMAN	Keratin, type I cuticular Ha8
35	ATPA_HUMAN	ATP synthase subunit alpha, mitochondrial
36	G3P_HUMAN	Glyceraldehyde-3-phosphate dehydrogenase
37	CH60_HUMAN	60 kDa heat shock protein, mitochondria
38	KRA44_HUMAN	Keratin-associated protein 4-4
39	PLAK_HUMAN	Junction plakoglobin
40	LEG7_HUMAN	Galectin-7
41	KRA98_HUMAN	Keratin-associated protein 9-8

**42**	DUS14_HUMAN	Dual specificity protein phosphatase 14
**43**	KRA93_HUMAN	Keratin-associated protein 9-3
**44**	TBA1B_HUMAN	Tubulin alpha-1B chain
**45**	ATPB_HUMAN	ATP synthase subunit beta, mitochondrial
**46**	LEG1_HUMAN	Galectin-1
**47**	MIF_HUMAN	Macrophage migration inhibitory factor
**48**	GDPD3_HUMAN	Glycerophosphodiester phosphodiesterase domain-containing protein 3
**49**	KRA99_HUMAN	Keratin-associated protein 9-9
**50**	ADT2_HUMAN	ADP/ATP translocase 2
**51**	PLP2_HUMAN	Proteolipid protein 2
**52**	KPYM_HUMAN	Pyruvate kinase PKM
**53**	H4_HUMAN	Histone H4
**54**	ACTG_HUMAN	Actin, cytoplasmic 2
**55**	KRA91_HUMAN	Keratin-associated protein 9-1
**56**	KRA97_HUMAN	Keratin-associated protein 9-7
**57**	ACTB_HUMAN	Actin, cytoplasmic 1
**58**	TPIS_HUMAN	Triosephosphate isomerase
**59**	PRDX6_HUMAN	Peroxiredoxin-6
**60**	EF2_HUMAN	Elongation factor 2
**61**	HSPB1_HUMAN	Heat shock protein beta-1
**62**	PMEL_HUMAN	Melanocyte protein PMEL
**63**	COF1_HUMAN	Cofilin-1
**64**	FABP5_HUMAN	Fatty acid-binding protein, epidermal
**65**	AMPL_HUMAN	Cytosol aminopeptidase
**66**	H2A2A_HUMAN	Histone H2A type 2-A
**67**	PPIA_HUMAN	Peptidyl-prolyl cis-trans isomerase A
**68**	KRT36_HUMAN	Keratin, type I cuticular Ha6
**69**	ANXA2_HUMAN	Annexin A2
**70**	HSP72_HUMAN	Heat shock-related 70 kDa protein 2
**71**	KR131_HUMAN	Keratin-associated protein 13-1
**72**	K2C1_HUMAN	Keratin, type II cytoskeletal 1
**73**	SCFD1_HUMAN	Sec1 family domain-containing protein 1
**74**	HCD2_HUMAN	3-hydroxyacyl-CoA dehydrogenase type-2
**75**	H2B2F_HUMAN	Histone H2B type 2-F
**76**	TBA4A_HUMAN	Tubulin alpha-4A chain
**77**	MDHM_HUMAN	Malate dehydrogenase, mitochondrial
**78**	KRT84_HUMAN	Keratin, type II cuticular Hb4
**79**	UK114_HUMAN	Ribonuclease UK114
**80**	TBB2A_HUMAN	Tubulin beta-2A chain
**81**	PKP3_HUMAN	Plakophilin-3
**82**	KRA21_HUMAN	Keratin-associated protein 2-1
**83**	GSTP1_HUMAN	Glutathione S-transferase P

**84**	ENOA_HUMAN	Alpha-enolase
**85**	RLA1_HUMAN	60S acidic ribosomal protein P1
**86**	DSC3_HUMAN	Desmocollin-3
**87**	CTNB1_HUMAN	Catenin beta-1
**88**	EF1A1_HUMAN	Elongation factor 1-alpha 1
**89**	KRA96_HUMAN	Keratin-associated protein 9-6
**90**	KRA15_HUMAN	Keratin-associated protein 1-5
**91**	K2C7_HUMAN	Keratin, type II cytoskeletal 7
**92**	PLCD1_HUMAN	1-phosphatidylinositol 4,5-bisphosphate phosphodiesterase delta-1
**93**	FA26D_HUMAN	Protein FAM26D
**94**	TERA_HUMAN	Transitional endoplasmic reticulum ATPase
**95**	RS27A_HUMAN	Ubiquitin-40S ribosomal protein S27a
**96**	1433E_HUMAN	14-3-3 protein epsilon
**97**	TBB4B_HUMAN	Tubulin beta-4B chain
**98**	DHB4_HUMAN	Peroxisomal multifunctional enzyme type 2
**99**	K2C80_HUMAN	Keratin, type II cytoskeletal 80
**100**	VINC_HUMAN	Vinculin
**101**	NEUR2_HUMAN	Sialidase-2
**102**	1433Z_HUMAN	14-3-3 protein zeta/delta
**103**	PADI3_HUMAN	Protein-arginine deiminase type-3
**104**	TGM1_HUMAN	Protein-glutamine gamma-glutamyltransferase K
**105**	ECHD1_HUMAN	Ethylmalonyl-CoA decarboxylase
**106**	BLMH_HUMAN	Bleomycin hydrolase
**107**	6PGL_HUMAN	6-phosphogluconolactonase
**108**	GPNMB_HUMAN	Transmembrane glycoprotein NMB
**109**	H33_HUMAN	Histone H3.3
**110**	S10AE_HUMAN	Protein S100-A14
**111**	PHB_HUMAN	Prohibitin
**112**	SSBP_HUMAN	Single-stranded DNA-binding protein, mitochondrial
**113**	GRP78_HUMAN	78 kDa glucose-regulated protein
**114**	KR241_HUMAN	Keratin-associated protein 24-1
**115**	RSSA_HUMAN	0S ribosomal protein SA
**116**	KRT36_HUMAN	Keratin, type I cuticular Ha6
**117**	VDAC2_HUMAN	Voltage-dependent anion-selective channel protein 2
**118**	K2C5_HUMAN	Keratin, type II cytoskeletal 5
**119**	C9JSQ1_HUMAN	Creatine kinase U-type, mitochondrial
**120**	F16P1_HUMAN	Fructose-1,6-bisphosphatase 1
**121**	THIK_HUMAN	3-ketoacyl-CoA thiolase, peroxisomal
**122**	ADT3_HUMAN	ADP/ATP translocase 3
**123**	1433G_HUMAN	14-3-3 protein gamma
**124**	KR195_HUMAN	Keratin-associated protein 19-5
**125**	HPHL1_HUMAN	Hephaestin-like protein 1

**126**	SYG_HUMAN	Glycine--tRNA ligase
**127**	ALDH2_HUMAN	Aldehyde dehydrogenase, mitochondrial
**128**	TBB5_HUMAN	Tubulin beta chain
**129**	LYG2_HUMAN	Lysozyme g-like protein 2
**130**	LDHB_HUMAN	L-lactate dehydrogenase B chain
**131**	PEPL_HUMAN	Periplakin
**132**	RAB7A_HUMAN	Ras-related protein Rab-7a
**133**	HEXB_HUMAN	Beta-hexosaminidase subunit beta
**134**	H32_HUMAN	Histone H3.2
**135**	CSRP1_HUMAN	Cysteine and glycine-rich protein 1
**136**	KRA81_HUMAN	Keratin-associated protein 8-1
**137**	K2C75_HUMAN	Keratin, type II cytoskeletal 75
**138**	KRA11_HUMAN	Keratin-associated protein 1-1
**139**	CRYAB_HUMAN	Alpha-crystallin B chain
**140**	KRA94_HUMAN	Keratin-associated protein 9-4
**141**	SPB5_HUMAN	Serpin B5
**142**	K1C10_HUMAN	Keratin, type I cytoskeletal 10
**143**	COMT_HUMAN	Catechol O-methyltransferase
**144**	KR161_HUMAN	Keratin-associated protein 16-1
**145**	GDIB_HUMAN	Rab GDP dissociation inhibitor beta
**146**	LRC15_HUMAN	Leucine-rich repeat-containing protein 15
**147**	C9J9S3_HUMAN	Serine/threonine-protein phosphatase
**148**	PGK1_HUMAN	Phosphoglycerate kinase 1
**149**	CLIC3_HUMAN	Chloride intracellular channel protein 3
**150**	H2B1J_HUMAN	Histone H2B type 1-J
**151**	TCTP_HUMAN	Translationally-controlled tumor protein
**152**	PRDX2_HUMAN	Peroxiredoxin-2
**153**	F5H608_HUMAN	ATP synthase subunit d, mitochondrial
**154**	PDIA6_HUMAN	Protein disulfide-isomerase A6
**155**	APT_HUMAN	Adenine phosphoribosyltransferase
**156**	H15_HUMAN	Histone H1.5
**157**	CISY_HUMAN	Citrate synthase, mitochondrial
**158**	H2AZ_HUMAN	Histone H2A.Z
**159**	FAS_HUMAN	Fatty acid synthase
**160**	H2AY_HUMAN	Core histone macro-H2A.1
**161**	HSP7C_HUMAN	Heat shock cognate 71 kDa protein
**162**	HYES_HUMAN	Bifunctional epoxide hydrolase 2
**163**	1433F_HUMAN	14-3-3 protein eta
**164**	HS71L_HUMAN	Heat shock 70 kDa protein 1-like
**165**	KR10A_HUMAN	Keratin-associated protein 10-10
**166**	CTNA1_HUMAN	Catenin alpha-1
**167**	EF1G_HUMAN	Elongation factor 1-gamma

**168**	S10AG_HUMAN	Protein S100-A16
**169**	K2C6A_HUMAN	Keratin, type II cytoskeletal 6A
**170**	H2B1N_HUMAN	Histone H2B type 1-N
**171**	SODC_HUMAN	Superoxide dismutase [Cu-Zn]
**172**	H2A3_HUMAN	Histone H2A type 3
**173**	K1C40_HUMAN	Keratin, type I cytoskeletal 40
**174**	ENDD1_HUMAN	Endonuclease domain-containing 1 protein
**175**	H12_HUMAN	Histone H1.2
**176**	ECHA_HUMAN	Trifunctional enzyme subunit alpha, mitochondrial
**177**	ACTS_HUMAN	Actin, alpha skeletal muscle
**178**	LMNA_HUMAN	Prelamin-A/C
**179**	KRA61_HUMAN	Keratin-associated protein 6-1
**180**	KRT37_HUMAN	Keratin, type I cuticular Ha7
**181**	ANXA1_HUMAN	Annexin A1
**182**	GFAP_HUMAN	Glial fibrillary acidic protein
**183**	COX2_HUMAN	Cytochrome c oxidase subunit 2
**184**	CATD_HUMAN	Cathepsin D
**185**	FA83H_HUMAN	Protein FAM83H
**186**	K1C19_HUMAN	Keratin, type I cytoskeletal 19
**187**	RLA2_HUMAN	60S acidic ribosomal protein P2
**188**	K22E_HUMAN	Keratin, type II cytoskeletal 2 epidermal
**189**	TRXR1_HUMAN	Thioredoxin reductase 1, cytoplasmic
**190**	CH10_HUMAN	10 kDa heat shock protein, mitochondrial
**191**	FIS1_HUMAN	Mitochondrial fission 1 protein
**192**	HNRH1_HUMAN	Heterogeneous nuclear ribonucleoprotein H
**193**	PRDX1_HUMAN	Peroxiredoxin-1
**194**	LAMP1_HUMAN	Lysosome-associated membrane glycoprotein 1
**195**	VATA_HUMAN	V-type proton ATPase catalytic subunit A
**196**	TAGL2_HUMAN	Transgelin-2
**197**	UBP5_HUMAN	Ubiquitin carboxyl-terminal hydrolase 5
**198**	GSDMA_HUMAN	Gasdermin-A

primary acc #	Ensembl Accession	count	abundance	GN
O43790	ENSP00000443169	54	6.617	KRT86
P78386	ENSP00000257901	54	6.584	KRT85
Q15323	ENSP00000251645	54	6.493	KRT31
Q14525	ENSP00000251646	54	6.309	KRT33B
O76009	ENSP00000007735	54	6.265	KRT33A
P78385	ENSP00000293670	53	6.204	KRT83
O76011	ENSP00000377570	54	6.059	KRT34
P00760	sp|TRY1_BOVIN|	54	5.876	
Q14533	ENSP00000369349	49	5.799	KRT81
Q9NSB4	ENSP00000257974	54	5.758	KRT82
Q9BYR8	ENSP00000375430	53	5.698	KRTAP3-1
Q8IUC1	ENSP00000330720	53	5.591	KRTAP11-1
Q9BYR7	ENSP00000375429	51	5.404	KRTAP3-2
P15924	ENSP00000369129	52	5.376	DSP
P13598	ENSP00000415283	50	5.274	ICAM2
Q5VU13	ENSP00000357080	54	5.241	VSIG8
Q14532	ENSP00000225899	51	5.201	KRT32
P33764	ENSP00000357702	52	5.173	S100A3
Q92764	ENSP00000377558	48	5.057	KRT35
Q86SJ6	ENSP00000311859	52	4.967	DSG4
Q13835	ENSP00000356293	51	4.940	PKP1
Q13228	ENSP00000357861	51	4.893	SELENBP1
Q9BYR4	ENSP00000375151	52	4.825	KRTAP4-3
Q9BYR6	ENSP00000375428	46	4.787	KRTAP3-3
Q6A163	ENSP00000347823	51	4.772	KRT39
P27482	ENSP00000315299	53	4.736	CALML3
Q9BYQ4	ENSP00000366950	45	4.732	KRTAP9-2
Q08188	ENSP00000370867	50	4.672	TGM3
Q52LG2	ENSP00000382777	51	4.610	KRTAP13-2
Q14134	ENSP00000343129	50	4.495	TRIM29
Q6PEX3	ENSP00000353742	52	4.471	KRTAP26-1
P31947	ENSP00000340989	49	4.470	SFN
P17931	ENSP00000254301	47	4.414	LGALS3
O76015	ENSP00000246646	46	4.390	KRT38
P25705	ENSP00000381736	46	4.365	ATP5A1
P04406	ENSP00000229239	46	4.204	GAPDH
P10809	ENSP00000373620	48	4.188	HSPD1
Q9BYR3	ENSP00000375076	45	4.186	KRTAP4-4
P14923	ENSP00000377507	43	4.126	JUP
P47929	ENSP00000367891	43	4.058	LGALS7
Q9BYQ0	ENSP00000254072	38	4.022	KRTAP9-8

O95147	ENSP00000377912	43	3.934	DUSP14
Q9BYQ3	ENSP00000392189	36	3.830	KRTAP9-3
P68363	ENSP00000336799	43	3.788	TUBA1B
P06576	ENSP00000262030	42	3.713	ATP5B
P09382	ENSP00000215909	44	3.650	LGALS1
P14174	ENSP00000215754	42	3.626	MIF
Q7L5L3	ENSP00000384363	43	3.597	GDPD3
Q9BYP9	ENSP00000415438	33	3.516	KRTAP9-9
P05141	ENSP00000360671	42	3.496	SLC25A5
Q04941	ENSP00000365505	41	3.450	PLP2
P14618	ENSP00000455736	40	3.377	PKM
P62805	ENSP00000376669	36	3.359	HIST1H4A
P63261	ENSP00000331514	34	3.282	ACTG1
A8MXZ3	ENSP00000381488	32	3.239	KRTAP9-1
A8MTY7	ENSP00000375149	31	3.228	KRTAP9-7
P60709	ENSP00000349960	33	3.212	ACTB
P60174	ENSP00000229270	36	3.186	TPI1
P30041	ENSP00000342026	38	3.176	PRDX6
P13639	ENSP00000307940	36	3.165	EEF2
P04792	ENSP00000248553	37	3.136	HSPB1
P40967	ENSP00000402758	37	3.011	PMEL
P23528	ENSP00000432660	36	2.936	CFL1
Q01469	ENSP00000297258	34	2.861	FABP5
P28838	ENSP00000226299	32	2.843	LAP3
Q6FI13	ENSP00000358158	31	2.827	HIST2H2AA3
P62937	ENSP00000419425	31	2.800	PPIA
O76013	ENSP00000377555	31	2.765	KRT36
P07355	ENSP00000379342	30	2.749	ANXA2
P54652	ENSP00000378199	30	2.708	HSPA2
Q8IUC0	ENSP00000347635	29	2.672	KRTAP13-1
P04264	ENSP00000252244	31	2.647	KRT1
Q8WVM8	ENSP00000390783	32	2.647	SCFD1
Q99714	ENSP00000168216	31	2.602	HSD17B10
Q5QNW6	ENSP00000358164	28	2.553	HIST2H2BF
P68366	ENSP00000248437	29	2.518	TUBA4A
P40926	ENSP00000327070	29	2.486	MDH2
Q9NSB2	ENSP00000257951	25	2.486	KRT84
P52758	ENSP00000254878	29	2.451	HRSP12
Q13885	ENSP00000369703	25	2.342	TUBB2A
Q9Y446	ENSP00000331678	26	2.289	PKP3
Q9BYU5	ENSP00000375238	26	2.286	KRTAP2-1
P09211	ENSP00000381607	27	2.285	GSTP1

P06733	ENSP00000234590	26	2.278	ENO1
P05386	ENSP00000346037	27	2.277	RPLP1
Q14574	ENSP00000353608	26	2.194	DSC3
P35222	ENSP00000385604	24	2.185	CTNNB
P68104	ENSP00000339063	26	2.174	EEF1A1
A8MVA2	ENSP00000375150	22	2.159	KRTAP9-6
Q9BYS1	ENSP00000355302	21	2.129	KRTAP1-5
P08729	ENSP00000444547	21	2.059	KRT7
P51178	ENSP00000430344	24	2.051	PLCD1
Q5JW98	ENSP00000385836	24	2.047	FAM26D
P55072	ENSP00000351777	23	1.962	VCP
P62979	ENSP00000383981	22	1.896	RPS27A
P62258	ENSP00000264335	21	1.864	YWHAE
P68371	ENSP00000341289	20	1.858	TUBB4B
P51659	ENSP00000256216	22	1.817	HSD17B4
Q6KB66	ENSP00000369361	22	1.769	KRT80
P18206	ENSP00000361841	22	1.764	VCL
Q9Y3R4	ENSP00000233840	21	1.715	NEU2
P63104	ENSP00000379287	19	1.667	YWHAZ
Q9ULW8	ENSP00000364609	20	1.659	PADI3
P22735	ENSP00000206765	20	1.658	TGM1
Q9NTX5	ENSP00000401751	20	1.633	ECHDC1
Q13867	ENSP00000261714	19	1.627	BLMH
O95336	ENSP00000252603	20	1.606	PGLS
Q14956	ENSP00000258733	18	1.597	GPNMB
P84243	ENSP00000355781	18	1.559	H3F3A
Q9HCY8	ENSP00000420296	19	1.551	S100A14
P35232	ENSP00000300408	19	1.548	PHB
Q04837	ENSP00000265304	20	1.546	SSBP1
P11021	ENSP00000324173	17	1.545	HSPA5
Q3LI83	ENSP00000339238	17	1.491	KRTAP24-1
P08865	ENSP00000346067	18	1.489	RPSA
O76013	ENSP00000329165	16	1.486	KRT36
P45880	ENSP00000298468	18	1.482	VDAC2
P13647	ENSP00000252242	16	1.478	KRT5
C9JSQ1	ENSP00000413999	17	1.429	CKMT1B
P09467	ENSP00000364475	17	1.409	FBP1
P09110	ENSP00000333664	17	1.401	ACAA1
P12236	ENSP00000370808	18	1.387	SLC25A6
P61981	ENSP00000306330	17	1.372	YWHAG
Q3LI72	ENSP00000334985	16	1.350	KRTAP19-5
Q6MZM0	ENSP00000313699	16	1.334	HEPHL1

P41250	ENSP00000373918	16	1.329	GARS
P05091	ENSP00000403349	16	1.324	ALDH2
P07437	ENSP00000339001	14	1.319	TUBB
Q86SG7	ENSP00000386939	15	1.288	LYG2
P07195	ENSP00000379386	16	1.260	LDHB
O60437	ENSP00000340510	15	1.233	PPL
P51149	ENSP00000265062	15	1.213	RAB7A
P07686	ENSP00000426285	15	1.194	HEXB
Q71DI3	ENSP00000333277	14	1.191	HIST2H3A
P21291	ENSP00000356275	15	1.189	CSRP1
Q8IUC2	ENSP00000332805	15	1.182	KRTAP8-1
O95678	ENSP00000252245	12	1.121	KRT75
Q07627	ENSP00000305975	11	1.093	KRTAP1-1
P02511	ENSP00000436051	13	1.086	CRYAB
Q9BYQ2	ENSP00000334922	10	1.084	KRTAP9-4
P36952	ENSP00000372221	13	1.072	SERPINB5
P13645	ENSP00000269576	12	1.066	KRT10
P21964	ENSP00000354511	13	1.060	COMT
A8MUX0	ENSP00000375147	13	1.053	KRTAP16-1
P50395	ENSP00000369538	13	1.043	GDI2
Q8TF66	ENSP00000306276	12	1.039	LRRC15
C9J9S3	ENSP00000398839	13	1.000	PPP1CB
P00558	ENSP00000362413	12	0.982	PGK1
O95833	ENSP00000419378	12	0.972	CLIC3
P06899	ENSP00000342886	10	0.971	HIST1H2BJ
P13693	ENSP00000431872	12	0.946	TPT1
P32119	ENSP00000301522	11	0.935	PRDX2
F5H608	ENSP00000437996	11	0.928	ATP5H
Q15084	ENSP00000272227	11	0.928	PDIA6
P07741	ENSP00000367615	12	0.925	APRT
P16401	ENSP00000330074	11	0.923	HIST1H1B
O75390	ENSP00000446779	11	0.897	CS
P0C0S5	ENSP00000296417	10	0.890	H2AFZ
P49327	ENSP00000304592	11	0.876	FASN
O75367	ENSP00000423563	10	0.871	H2AFY
P11142	ENSP00000227378	9	0.869	HSPA8
P34913	ENSP00000430269	11	0.859	EPHX2
Q04917	ENSP00000248975	10	0.852	YWHAH
P34931	ENSP00000364805	9	0.843	HSPA1L
P60014	ENSP00000369438	10	0.841	KRTAP10-10
P35221	ENSP00000304669	10	0.839	CTNNA1
P26641	ENSP00000331901	10	0.834	EEF1G

Q96FQ6	ENSP00000357693	10	0.826	S100A16
P02538	ENSP00000369317	9	0.820	KRT6A
Q99877	ENSP00000446031	8	0.811	HIST1H2BN
P00441	ENSP00000270142	10	0.811	SOD1
Q7L7L0	ENSP00000355656	9	0.804	HIST3H2A
Q6A162	ENSP00000381500	8	0.804	KRT40
O94919	ENSP00000278505	10	0.780	ENDOD1
P16403	ENSP00000339566	9	0.757	HIST1H1C
P40939	ENSP00000370023	9	0.731	HADHA
P68133	ENSP00000355645	8	0.689	ACTA1
P02545	ENSP00000357283	8	0.675	LMNA
Q3LI64	ENSP00000332690	9	0.673	KRTAP6-1
O76014	ENSP00000225550	7	0.653	KRT37
P04083	ENSP00000257497	8	0.646	ANXA1
P14136	ENSP00000253408	7	0.645	GFAP
P00403	ENSP00000354876	8	0.636	MT-CO2
P07339	ENSP00000415840	8	0.625	CTSD
Q6ZRV2	ENSP00000373565	8	0.619	FAM83H
P08727	ENSP00000355124	7	0.608	KRT19
P05387	ENSP00000322419	7	0.603	RPLP2
P35908	ENSP00000310861	7	0.593	KRT2
Q16881	ENSP00000412045	7	0.592	TXNRD1
P61604	ENSP00000233893	7	0.587	HSPE1
Q9Y3D6	ENSP00000223136	7	0.571	FIS1
P31943	ENSP00000377082	7	0.568	HNRNPH1
Q06830	ENSP00000361152	7	0.567	PRDX1
P11279	ENSP00000333298	7	0.564	LAMP1
P38606	ENSP00000273398	7	0.560	ATP6V1A
P37802	ENSP00000357077	7	0.542	TAGLN2
P45974	ENSP00000229268	7	0.534	USP5
Q96QA5	ENSP00000301659	7	0.522	GSDMA
