# Supplementary material for: Demonstration of Protein-Based Human Identification Using the Hair Shaft Proteome
Source: PLoS One. 2016 Sep 7;11(9):e0160653. doi: 10.1371/journal.pone.0160653 (PMC5014411; doi:10.1371/journal.pone.0160653)
Supplement: S3 File — Datasets from a subset of European–American Subjects (EA1, L1.001 to L1.060) were processed for absolute abundance values using the X!Tandem algorithm (www.thegpm.org) and sorted according to the absolute abundance values in the proteome. Overall abundance values were generated by the following formula: abundance values were averaged and multiplied by the quotient of number of datasets with the detected gene product by the total number of datasets (abundance; n = 54). Each gene product (Ensembl Accession) and proportion of individuals with the detected gene product (count) are described. Primary protein accession numbers (primary acc#), and the mnemonic identifier of a UniProtKB entry was entered (UNIPROT#), along with protein name (protein name) and gene name (GN). Duplicate entries were pooled. Gene products that were detected in less than 7 individuals were not analyzed. (PDF) [file pone.0160653.s006.pdf]

UniProt ID	Overall abundance	observation	Accession #	GN	Ensembl Accession #	fn
K1H1_HUMAN	8.141	22	Q15323	KRT31	ENSP00000251645	s
KT33B_HUMAN	8.094	22	Q14525	KRT33B	ENSP00000251646	s
KRT86_HUMAN	8.018	22	O43790	KRT86	ENSP00000443169	s
KT33A_HUMAN	7.995	22	O76009	KRT33A	ENSP00000007735	s
KRT83_HUMAN	7.895	22	P78385	KRT83	ENSP00000293670	s
KRT34_HUMAN	7.846	22	O76011	KRT34	ENSP00000377570	s
KRT85_HUMAN	7.815	22	P78386	KRT85	ENSP00000257901	s
KRT35_HUMAN	7.393	22	Q92764	KRT35	ENSP00000246639	s
KRT81_HUMAN	7.368	22	Q14533	KRT81	ENSP00000369349	s
K1H2_HUMAN	7.266	22	Q14532	KRT32	ENSP00000225899	s
KRT82_HUMAN	7.195	22	Q9NSB4	KRT82	ENSP00000257974	s
KRT36_HUMAN	6.051	22	O76013	KRT36	ENSP00000377555	s
KRT84_HUMAN	5.595	22	Q9NSB2	KRT84	ENSP00000257951	s
DSG4_HUMAN	5.560	20	Q86SJ6	DSG4	ENSP00000311859	s
DESP_HUMAN	5.514	19	P15924	DSP	ENSP00000369129	s
K1C39_HUMAN	5.320	22	Q6A163	KRT39	ENSP00000347823	s
SBP1_HUMAN	5.265	19	Q13228	SELENBP1	ENSP00000357861	pm
PKP1_HUMAN	5.148	19	Q13835	PKP1	ENSP00000356293	s
VSIG8_HUMAN	5.042	19	Q5VU13	VSIG8	ENSP00000357080	pm
PLAK_HUMAN	5.037	20	P14923	JUP	ENSP00000377507	s
TGM3_HUMAN	4.897	19	Q08188	TGM3	ENSP00000370867	m
LEG7_HUMAN	4.622	18	P47929	LGALS7	ENSP00000367891	ex
CALL3_HUMAN	4.434	16	P27482	CALML3	ENSP00000315299	pr
1433S_HUMAN	4.275	17	P31947	SFN	ENSP00000340989	pr
K2C1_HUMAN	4.264	17	P04264	KRT1	ENSP00000252244	s
LEG3_HUMAN	4.075	16	P17931	LGALS3	ENSP00000254301	ex
ATPA_HUMAN	4.017	16	P25705	ATP5A1	ENSP00000381736	mt
KRT38_HUMAN	3.980	19	O76015	KRT38	ENSP00000246646	s
ANXA2_HUMAN	3.926	16	P07355	ANXA2	ENSP00000379342	ml
ATPB_HUMAN	3.905	16	P06576	ATP5B	ENSP00000262030	mt
G3P_HUMAN	3.818	16	P04406	GAPDH	ENSP00000229239	m
KR241_HUMAN	3.746	18	Q3LI83	KRTAP24-1	ENSP00000339238	s
PPIA_HUMAN	3.639	17	P62937	PPIA	ENSP00000405975	pr
Q8WU19_HUMAN	3.619	18	Q8WU19	TUBA1B	ENSP00000336799	s
HPHL1_HUMAN	3.602	15	Q6MZM0	HEPHL1	ENSP00000313699	pm
DUS14_HUMAN	3.565	16	O95147	DUSP14	ENSP00000377912	pr
LRC15_HUMAN	3.560	16	Q8TF66	LRRC15	ENSP00000306276	pm
ENOA_HUMAN	3.547	16	P06733	ENO1	ENSP00000234590	m
1433E_HUMAN	3.530	16	P62258	YWHAE	ENSP00000264335	pr
K1C10_HUMAN	3.509	18	P13645	KRT10	ENSP00000269576	s
TBA4A_HUMAN	3.476	16	P68366	TUBA4A	ENSP00000248437	s
H2AY_HUMAN	3.438	18	O75367	H2AFY	ENSP00000423563	n
PLCD1_HUMAN	3.415	16	P51178	PLCD1	ENSP00000430344	m
AMPL_HUMAN	3.385	16	P28838	LAP3	ENSP00000226299	m
GSTP1_HUMAN	3.383	17	P09211	GSTP1	ENSP00000381607	m
K2C7_HUMAN	3.357	20	P08729	KRT7	ENSP00000329243	s

HSP7C_HUMAN	3.321	16 P11142	HSPA8	ENSP00000437125	pr
PKP3_HUMAN	3.318	17 Q9Y446	PKP3	ENSP00000331678	s
BLMH_HUMAN	3.302	16 Q13867	BLMH	ENSP00000261714	m
GRP78_HUMAN	3.265	16 P11021	HSPA5	ENSP00000324173	pr
MDHM_HUMAN	3.228	16 P40926	MDH2	ENSP00000327070	mt
FA26D_HUMAN	3.218	16 Q5JW98	FAM26D	ENSP00000357585	pm
TBB2A_HUMAN	3.175	16 Q13885	TUBB2A	ENSP00000369703	s
ALDH2_HUMAN	3.130	16 P05091	ALDH2	ENSP00000261733	mt
CTNB1_HUMAN	3.067	16 P35222	CTNNB1	ENSP00000385604	pm
S10A3_HUMAN	3.052	16 P33764	S100A3	ENSP00000357702	u
EF1A1_HUMAN	3.052	16 P68104	EEF1A1	ENSP00000339063	pr
VDAC2_HUMAN	3.044	15 P45880	VDAC2	ENSP00000298468	pm
GPNMB_HUMAN	3.043	16 Q14956	GPNMB	ENSP00000258733	pm
SPB5_HUMAN	3.005	15 P36952	SERPINB5	ENSP00000372221	ex
DHB4_HUMAN	2.944	16 P51659	HSD17B4	ENSP00000256216	m
FABP4_HUMAN	2.931	14 P15090	FABP4	ENSP00000256104	m
CH60_HUMAN	2.931	16 P10809	HSPD1	ENSP00000373620	pr
RS27A_HUMAN	2.900	16 P62979	RPS27A	ENSP00000383981	pr
PRDX6_HUMAN	2.869	16 P30041	PRDX6	ENSP00000342026	m
1433Z_HUMAN	2.838	15 P63104	YWHAZ	ENSP00000379287	pr
H2A2A_HUMAN	2.806	14 Q6FI13	HIST2H2AA3	ENSP00000358158	n
LMNA_HUMAN	2.797	15 P02545	LMNA	ENSP00000357284	n
TPIS_HUMAN	2.766	15 P60174	TPI1	ENSP00000229270	m
HCD2_HUMAN	2.748	15 Q99714	HSD17B10	ENSP00000168216	mt
K1C40_HUMAN	2.737	15 Q6A162	KRT40	ENSP00000381500	s
K2C6A_HUMAN	2.730	16 P02538	KRT6A	ENSP00000369317	s
KPYM_HUMAN	2.707	16 P14618	PKM	ENSP00000455736	m
GDPD3_HUMAN	2.646	14 Q7L5L3	GDPD3	ENSP00000384363	mb
UK114_HUMAN	2.638	15 P52758	HRSP12	ENSP00000254878	pr
EF2_HUMAN	2.636	15 P13639	EEF2	ENSP00000307940	pr
K2C75_MOUSE	2.620	15 Q8BGZ7	KRT75	ENSP00000252245	s
ACTB_HUMAN	2.586	16 P60709	ACTB	ENSP00000349960	s
H2B1J_HUMAN	2.529	13 P06899	HIST1H2BJ	ENSP00000342886	n
H15_HUMAN	2.514	14 P16401	HIST1H1B	ENSP00000330074	n
KRT37_HUMAN	2.431	15 O76014	KRT37	ENSP00000225550	s
RLA2_HUMAN	2.420	13 P05387	RPLP2	ENSP00000322419	pr
RSSA_HUMAN	2.342	14 P08865	RPSA	ENSP00000346067	pr
K2C80_HUMAN	2.301	14 Q6KB66	KRT80	ENSP00000369361	s
LAMP1_HUMAN	2.299	14 P11279	LAMP1	ENSP00000333298	l
H2AZ_HUMAN	2.292	14 P0C0S5	H2AFZ	ENSP00000296417	n
1433B_HUMAN	2.279	12 P31946	YWHAB	ENSP00000300161	pr
HSPB1_HUMAN	2.255	14 P04792	HSPB1	ENSP00000248553	pr
VDAC1_HUMAN	2.253	14 P21796	VDAC1	ENSP00000265333	mt
ROA1_HUMAN	2.247	15 P09651	HNRNPA1	ENSP00000448617	pr
K22E_HUMAN	2.234	12 P35908	KRT2	ENSP00000310861	s
LDHB_HUMAN	2.201	12 P07195	LDHB	ENSP00000379386	m
LMNB1_HUMAN	2.144	14 P20700	LMNB1	ENSP00000261366	pm

TERA_HUMAN	2.108	14	P55072	VCP	ENSP00000351777	m
TGM1_HUMAN	2.108	12	P22735	TGM1	ENSP00000206765	m
COF1_HUMAN	2.088	11	P23528	CFL1	ENSP00000432660	s
GGH_HUMAN	2.077	13	Q92820	GGH	ENSP00000260118	m
H2B1B_HUMAN	2.076	12	P33778	HIST1H2BB	ENSP00000350580	n
H2AX_HUMAN	2.070	13	P16104	H2AFX	ENSP00000364310	n
ACADV_HUMAN	2.058	14	P49748	ACADVL	ENSP00000349297	mt
GFAP_HUMAN	2.058	13	P14136	GFAP	ENSP00000253408	s
CALL5_HUMAN	2.035	13	Q9NZT1	CALML5	ENSP00000369689	pr
TBB4B_HUMAN	2.025	13	P68371	TUBB4B	ENSP00000341289	s
H2A1_HUMAN	2.019	13	P0C0S8	HIST1H2AK	ENSP00000330307	n
AIM1_HUMAN	2.005	14	Q9Y4K1	AIM1	ENSP00000358062	u
K2C8_HUMAN	2.002	14	P05787	KRT8	ENSP00000447566	s
HNRH1_HUMAN	1.987	14	P31943	HNRNPH1	ENSP00000377082	pr
LYG2_HUMAN	1.978	12	Q86SG7	LYG2	ENSP00000386939	ex
TRXR1_HUMAN	1.931	11	Q16881	TXNRD1	ENSP00000434516	m
ECHA_HUMAN	1.922	13	P40939	HADHA	ENSP00000370023	mt
GDIB_HUMAN	1.916	11	P50395	GDI2	ENSP00000369538	mb
H32_HUMAN	1.914	12	Q71DI3	HIST2H3D	ENSP00000333277	n
H14_MOUSE	1.913	14	P43274	HIST1H1E	ENSP00000307705	n
CADH1_HUMAN	1.897	13	P12830	CDH1	ENSP00000261769	pm
PLD3_HUMAN	1.884	13	Q8IV08	PLD3	ENSP00000386293	m
KRA31_HUMAN	1.864	10	Q9BYR8	KRTAP3-1	ENSP00000375430	s
C9JSQ1_HUMAN	1.863	12	C9JSQ1	CKMT1B	ENSP00000413999	mt
ANXA1_HUMAN	1.863	12	P04083	ANXA1	ENSP00000257497	pm
DSC3_HUMAN	1.860	12	Q14574	DSC3	ENSP00000353608	s
KRA32_HUMAN	1.860	10	Q9BYR7	KRTAP3-2	ENSP00000375429	s
PGK1_HUMAN	1.808	12	P00558	PGK1	ENSP00000362413	m
MIF_HUMAN	1.793	12	P14174	MIF	ENSP00000215754	u
CH10_HUMAN	1.791	11	P61604	HSPE1	ENSP00000233893	pr
S10AE_HUMAN	1.783	12	Q9HCY8	S100A14	ENSP00000420296	pr
CISY_HUMAN	1.773	12	O75390	CS	ENSP00000342056	mt
PHB_HUMAN	1.771	12	P35232	PHB	ENSP00000300408	mt
HS71L_HUMAN	1.769	12	P34931	HSPA1L	ENSP00000364805	pr
H2B1C_HUMAN	1.766	12	P62807	HIST1H2BE	ENSP00000348924	n
KR195_HUMAN	1.766	9	Q3LI72	KRTAP19-5	ENSP00000334985	s
LDHA_HUMAN	1.734	11	P00338	LDHA	ENSP00000395337	m
H12_HUMAN	1.722	12	P16403	HIST1H1C	ENSP00000339566	n
PADI3_HUMAN	1.715	11	Q9ULW8	PADI3	ENSP00000364609	pr
1433T_HUMAN	1.688	10	P27348	YWHAQ	ENSP00000238081	pr
NEUR2_HUMAN	1.686	11	Q9Y3R4	NEU2	ENSP00000233840	ex
ADT2_HUMAN	1.668	10	P05141	SLC25A5	ENSP00000360671	mt
ACTG_HUMAN	1.651	10	P63261	ACTG1	ENSP00000331514	s
HEXB_HUMAN	1.648	10	P07686	HEXB	ENSP00000261416	m
HS90A_HUMAN	1.644	11	P07900	HSP90AA1	ENSP00000216281	pr
KRA33_HUMAN	1.643	9	Q9BYR6	KRTAP3-3	ENSP00000375428	s
H2B1N_HUMAN	1.632	9	Q99877	HIST1H2BN	ENSP00000380177	n

F16P1_HUMAN	1.625	10	P09467	FBP1	ENSP00000364475	m
EF1G_HUMAN	1.582	9	P26641	EEF1G	ENSP00000331901	pr
ACTBL_HUMAN	1.581	12	Q562R1	ACTBL2	ENSP00000416706	s
H2A3_HUMAN	1.560	12	Q7L7L0	HIST3H2A	ENSP00000355656	n
K2C5_HUMAN	1.532	12	P13647	KRT5	ENSP00000252242	s
KRA92_HUMAN	1.520	10	Q9BYQ4	KRTAP9-2	ENSP00000366950	s
PABP1_HUMAN	1.518	11	P11940	PABPC1	ENSP00000313007	pr
Q5TEC6_HUMAN	1.505	11	Q5TEC6	HIST2H3PS2	ENSP00000376675	n
THIK_HUMAN	1.501	12	P09110	ACAA1	ENSP00000333664	m
RL_HUMAN	1.470	10	P05388	RPLP0	ENSP00000376299	pr
ENASE_HUMAN	1.466	12	Q8NFI3	ENGASE	ENSP00000438577	
H2A1H_HUMAN	1.460	11	Q96KK5	HIST1H2AH	ENSP00000366679	n
PROF1_HUMAN	1.454	12	P07737	PFN1	ENSP00000225655	s
B5MDD6_HUMAN	1.417	8	B5MDD6	KRTAP9-9	ENSP00000377576	s
K1C9_HUMAN	1.416	11	P35527	KRT9	ENSP00000246662	s
TBB5_HUMAN	1.414	13	P07437	TUBB	ENSP00000339001	s
H2A1D_HUMAN	1.400	11	P20671	HIST1H2AD	ENSP00000341094	n
CLH1_HUMAN	1.392	9	Q00610	CLTC	ENSP00000269122	pm
KRA98_HUMAN	1.387	8	Q9BYQ0	KRTAP9-8	ENSP00000254072	s
AATM_HUMAN	1.387	11	P00505	GOT2	ENSP00000245206	mt
RS3_HUMAN	1.387	11	P23396	RPS3	ENSP00000434643	pr
KRA93_HUMAN	1.373	8	Q9BYQ3	KRTAP9-3	ENSP00000392189	s
KRA43_HUMAN	1.368	10	Q9BYR4	KRTAP4-3	ENSP00000375151	s
LMNB2_HUMAN	1.357	10	Q03252	LMNB2	ENSP00000327054	n
H2B2F_HUMAN	1.355	9	Q5QNW6	HIST2H2BF	ENSP00000358164	n
TBA1A_HUMAN	1.352	11	Q71U36	TUBA1A	ENSP00000301071	s
CRYAB_HUMAN	1.351	10	P02511	CRYAB	ENSP00000436051	pr
HSP76_HUMAN	1.325	11	P17066	HSPA6	ENSP00000310219	pr
ADT3_HUMAN	1.323	9	P12236	SLC25A6	ENSP00000370808	mt
SPB13_HUMAN	1.303	10	Q9UIV8	SERPINB13	ENSP00000341584	pr
FAHD1_HUMAN	1.301	10	Q6P587	FAHD1	ENSP00000372114	mt
KRA21_HUMAN	1.301	8	Q9BYU5	KRTAP2-1	ENSP00000375238	s
PEBP1_HUMAN	1.293	12	P30086	PEBP1	ENSP00000261313	c
KR111_HUMAN	1.287	9	Q8IUC1	KRTAP11-1	ENSP00000330720	s
PHB2_HUMAN	1.287	10	Q99623	PHB2	ENSP00000441875	mb
THIO_HUMAN	1.284	12	P10599	TXN	ENSP00000363641	m
H7C469_HUMAN	1.283	10	H7C469	RP11-295K3.1	ENSP00000415840	u
GSDMA_HUMAN	1.282	9	Q96QA5	GSDMA	ENSP00000301659	pm
EF1B_HUMAN	1.266	10	P24534	EEF1B2	ENSP00000236957	pr
FAS_HUMAN	1.238	8	P49327	FASN	ENSP00000304592	m
PLEC_HUMAN}	1.234	11	Q15149	PLEC	ENSP00000344848	s
SFPQ_HUMAN	1.232	10	P23246	SFPQ	ENSP00000349748	pr
H2A2C_HUMAN	1.232	9	Q16777	HIST2H2AC	ENSP00000332194	n
TAGL2_HUMAN	1.223	11	P37802	TAGLN2	ENSP00000357077	s
K2C79_HUMAN	1.222	10	Q5XKE5	KRT79	ENSP00000328358	s
K1C14_HUMAN	1.211	10	P02533	KRT14	ENSP00000167586	s
H10_HUMAN	1.200	9	P07305	H1F0	ENSP00000344504	n

TRI29_HUMAN	1.194	10	Q14134	TRIM29	ENSP00000343129	s
ARF1_HUMAN	1.185	8	P84077	ARF1	ENSP00000272102	mb
PEPL_HUMAN	1.170	10	O60437	PPL	ENSP00000340510	s
ECHD1_HUMAN	1.157	8	Q9NTX5	ECHDC1	ENSP00000401751	m
PPAP_HUMAN	1.152	9	P15309	ACPP	ENSP00000337471	m
PRDX2_HUMAN	1.146	8	P32119	PRDX2	ENSP00000301522	m
RTN4_HUMAN	1.143	10	Q9NQC3	RTN4	ENSP00000337838	mb
FABP5_HUMAN	1.138	11	Q01469	FABP5	ENSP00000297258	m
ARL8B_HUMAN	1.125	11	Q9NVJ2	ARL8B	ENSP00000256496	mb
FIS1_HUMAN	1.121	12	Q9Y3D6	FIS1	ENSP00000223136	mt
S10AG_HUMAN	1.117	9	Q96FQ6	S100A16	ENSP00000357693	n
TKT_HUMAN	1.110	8	P29401	TKT	ENSP00000417773	m
1433G_HUMAN	1.102	8	P61981	YWHAG	ENSP00000306330	pr
H2A1B_HUMAN	1.098	9	P04908	HIST1H2AB	ENSP00000259791	n
CALM_HUMAN	1.095	9	P62158	CALM2	ENSP00000272298	pr
RLA1_HUMAN	1.083	9	P05386	RPLP1	ENSP00000346037	pr
UBB_HUMAN	1.078	8	P0CG47	UBB	ENSP00000304697	pr
ALDOA_HUMAN	1.066	8	P04075	ALDOA	ENSP00000336927	m
H2A1C_HUMAN	1.064	9	Q93077	HIST1H2AC	ENSP00000367022	n
LEG1_HUMAN	1.062	9	P09382	LGALS1	ENSP00000215909	ex
CTNA1_HUMAN	1.061	7	P35221	CTNNA1	ENSP00000304669	pm
HSP71_HUMAN	1.060	9	P08107	HSPA1A	ENSP00000364802	pr
ECHB_HUMAN	1.054	8	P55084	HADHB	ENSP00000325136	mt
H2B1L_HUMAN	1.039	8	Q99880	HIST1H2BL	ENSP00000366618	n
PPCE_HUMAN	1.037	9	P48147	PREP	ENSP00000358106	pr
KR131_HUMAN	1.036	8	Q8IUC0	KRTAP13-1	ENSP00000347635	s
GRP75_HUMAN	1.024	10	P38646	HSPA9	ENSP00000297185	pr
HXK1_HUMAN	1.019	10	P19367	HK1	ENSP00000352398	m
PARK7_HUMAN	1.016	8	Q99497	PARK7	ENSP00000340278	m
PDIA1_HUMAN	1.013	8	P07237	P4HB	ENSP00000327801	pr
CATA_HUMAN	1.003	9	P04040	CAT	ENSP00000241052	m
E9PRY8_HUMAN	0.998	9	E9PRY8	EEF1D	ENSP00000434070	pr
IF4A1_HUMAN	0.993	7	P60842	EIF4A1	ENSP00000293831	pr
G6PI_HUMAN	0.982	7	P06744	GPI	ENSP00000348877	m
K1C19_HUMAN	0.980	8	P08727	KRT19	ENSP00000355124	s
RAB7A_HUMAN	0.974	9	P51149	RAB7A	ENSP00000265062	mb
KRA47_HUMAN	0.974	6	Q9BYR0	KRTAP4-7	ENSP00000375236	s
CAN12_HUMAN	0.971	10	Q6ZSI9	CAPN12	ENSP00000331636	pr
LAMP2_HUMAN	0.965	8	P13473	LAMP2	ENSP00000408411	l
S10AB_HUMAN	0.962	10	P31949	S100A11	ENSP00000271638	n
RS2_HUMAN	0.959	7	P15880	RPS2	ENSP00000341885	pr
C1QBP_HUMAN	0.958	9	Q07021	C1QBP	ENSP00000225698	ex
APOD_HUMAN	0.957	9	P05090	APOD	ENSP00000345179	ex
C9JJT5_HUMAN	0.949	9	C9JJT5	ATP5J2-PTCD1	ENSP00000416954	u
K1C16_HUMAN	0.937	8	P08779	KRT16	ENSP00000301653	s
SERA_HUMAN	0.936	8	O43175	PHGDH	ENSP00000358417	m
COMT_HUMAN	0.934	9	P21964	COMT	ENSP00000354511	m

KRA44_HUMAN	0.916	6	Q9BYR3	KRTAP4-4	ENSP00000375076	s
RAN_HUMAN	0.912	8	P62826	RAN	ENSP00000446215	mb
KRA97_HUMAN	0.911	6	A8MTY7	KRTAP9-7	ENSP00000375149	s
PLP2_HUMAN	0.889	9	Q04941	PLP2	ENSP00000365505	pm
HS90B_HUMAN	0.888	9	P08238	HSP90AB1	ENSP00000360709	pr
AHNK_HUMAN	0.888	9	Q09666	AHNAK	ENSP00000367263	u
K2C1B_HUMAN	0.879	8	Q7Z794	KRT77	ENSP00000342710	s
MBOA5_HUMAN	0.872	8	Q6P1A2	LPCAT3	ENSP00000261407	m
H2B3B_HUMAN	0.866	7	Q8N257	HIST3H2BB	ENSP00000375736	n
EFHD1_HUMAN	0.865	7	Q9BUP0	EFHD1	ENSP00000264059	mt
H4_HUMAN	0.852	7	P62805			n
IMB1_HUMAN	0.852	8	Q14974	KPNB1	ENSP00000290158	n
KRA46_HUMAN	0.812	5	Q9BYQ5	KRTAP4-6	ENSP00000328270	s
KR411_HUMAN	0.810	5	Q9BYQ6	KRTAP4-11	ENSP00000375232	s
KR412_HUMAN	0.810	5	Q9BQ66	KRTAP4-12	ENSP00000377582	s
KRA49_HUMAN	0.806	5	Q9BYQ8	KRTAP4-9	ENSP00000375234	s
NPC1_HUMAN	0.805	8	O15118	NPC1	ENSP00000269228	l
F5H608_HUMAN	0.804	6	F5H608	ATP5H	ENSP00000437996	mt
KRA45_HUMAN	0.804	5	Q9BYR2	KRTAP4-5	ENSP00000340546	s
CUX2_HUMAN	0.797	8	O14529	CUX2	ENSP00000261726	n
VDAC3_HUMAN	0.796	7	Q9Y277	VDAC3	ENSP00000428845	mt
KRA81_HUMAN	0.793	6	Q8IUC2	KRTAP8-1	ENSP00000332805	s
SYG_HUMAN	0.791	5	P41250			pr
H33_HUMAN	0.780	6	P84243	H3F3A	ENSP00000355781	n
CLIC1_HUMAN	0.780	7	O00299	CLIC1	ENSP00000364940	pm
IDH3A_HUMAN	0.776	8	P50213	IDH3A	ENSP00000299518	mt
PDIA6_HUMAN	0.771	5	Q15084			pr
RDHE2_HUMAN	0.762	7	Q8N3Y7	SDR16C5	ENSP00000307607	m
KRA42_HUMAN	0.760	5	Q9BYR5	KRTAP4-2	ENSP00000366955	s
RAB1B_HUMAN	0.747	7	Q9H0U4	RAB1B	ENSP00000310226	mb
VIME_HUMAN	0.724	5	P08670	VIM	ENSP00000224237	s
CATD_HUMAN	0.722	8	P07339	CTSD	ENSP00000236671	ml
RAB10_HUMAN	0.719	7	P61026	RAB10	ENSP00000264710	mb
PPIB_HUMAN	0.717	7	P23284	PPIB	ENSP00000300026	pr
ACTN1_HUMAN	0.710	6	P12814	ACTN1	ENSP00000193403	s
K1C13_HUMAN	0.710	7	P13646	KRT13	ENSP00000246635	s
CYTB_HUMAN	0.703	5	P04080	CSTB	ENSP00000291568	pr
SODC_HUMAN	0.700	7	P00441	SOD1	ENSP00000270142	m
GRAP1_HUMAN	0.697	5	Q4V328	GRIPAP1	ENSP00000365608	mb
ENPL_HUMAN	0.695	7	P14625	HSP90B1	ENSP00000299767	pr
LYPA1_HUMAN	0.695	7	O75608	LYPLA1	ENSP00000320043	m
SSBP_HUMAN	0.682	7	Q04837	SSBP1	ENSP00000265304	mt
KR132_HUMAN	0.674	6	Q52LG2			s
K1C24_HUMAN	0.674	7	Q2M2I5	KRT24	ENSP00000264651	s
ROA3_HUMAN	0.670	6	P51991	HNRNPA3	ENSP00000376309	n
QCR2_HUMAN	0.666	6	P22695	UQCRC2	ENSP00000268379	mt
COX2_HUMAN	0.666	8	P00403	MT-CO2	ENSP00000354876	mt

	0.665	7	P62701	RPS4X	ENSP00000362744	pr
CPT1A_HUMAN	0.664	7	P50416	CPT1A	ENSP00000439084	mt
IF4A2_HUMAN	0.659	7	Q14240	EIF4A2	ENSP00000326381	pr
RL11_HUMAN	0.657	7	P62913	RPL11	ENSP00000363676	pr
ECHM_HUMAN	0.655	6	P30084	ECHS1	ENSP00000357535	mt
ACTS_HUMAN	0.650	6	P68133	ACTA1	ENSP00000355645	s
SCFD1_HUMAN	0.648	7	Q8WVM8	SCFD1	ENSP00000390783	mb
DEST_HUMAN	0.640	7	P60981	DSTN	ENSP00000246069	s
MPCP_HUMAN	0.638	5	Q00325	SLC25A3	ENSP00000383898	mt
HNRPC_HUMAN	0.637	6	P07910	HNRNPC	ENSP00000338095	pr
PGAM1_HUMAN	0.632	4	P18669	PGAM1	ENSP00000359991	m
H2AW_HUMAN	0.631	6	Q9P0M6	H2AFY2	ENSP00000362352	n
KR161_HUMAN	0.627	5	A8MUX0	KRTAP16-1	ENSP00000375147	s
FUMH_HUMAN	0.623	6	P07954	FH	ENSP00000355518	mt
NONO_HUMAN	0.621	7	Q15233	NONO	ENSP00000276079	n
KR10B_HUMAN	0.621	5	P60412	KRTAP10-11	ENSP00000334197	s
BACH_HUMAN	0.614	7	O00154	ACOT7	ENSP00000367086	m
ACBP_HUMAN	0.613	7	P07108	DBI	ENSP00000348116	mb
RL13A_HUMAN	0.610	6	P40429	RPL13A	ENSP00000375730	pr
QCR1_HUMAN	0.596	5	P31930	UQCRC1	ENSP00000203407	pr
ROA2_HUMAN	0.594	6	P22626	HNRNPA2B1	ENSP00000346694	pr
RINI_HUMAN	0.588	6	P13489	RNH1	ENSP00000433999	pr
CXAR_HUMAN	0.582	6	P78310	CXADR	ENSP00000284878	pm
RL6_HUMAN	0.581	6	Q02878	RPL6	ENSP00000202773	pr
Q32Q12_HUMAN	0.580	4	Q32Q12	NME1-NME2	ENSP00000451932	m
THIM_HUMAN	0.578	4	P42765	ACAA2	ENSP00000285093	mt
H2A2B_HUMAN	0.575	4			Q8IUE6	n
H13_HUMAN	0.571	5	P16402	HIST1H1D	ENSP00000244534	n
PERI_HUMAN	0.569	4	P41219	PRPH	ENSP00000257860	s
DNSL2_HUMAN	0.569	5	Q92874	DNASE1L2	ENSP00000454562	n
ERH_HUMAN	0.568	6	P84090	ERH	ENSP00000451080	u
VATB2_HUMAN	0.562	5	P21281	ATP6V1B2	ENSP00000276390	mb
G6PD_HUMAN	0.557	7	P11413	G6PD	ENSP00000377192	m
Q5T8U2_HUMAN	0.556	6	Q5T8U2	RPL7A	ENSP00000361071	pr
VATA_HUMAN	0.552	6	P38606	ATP6V1A	ENSP00000273398	pm
ATPO_HUMAN	0.551	5	P48047	ATP5O	ENSP00000290299	mt
RL12_HUMAN	0.547	5	P30050			pr
KC1D_HUMAN	0.547	5	P48730	CSNK1D	ENSP00000324464	pr
RS3A_HUMAN	0.546	6	P61247	RPS3A	ENSP00000346050	pr
6PGL_HUMAN	0.534	5	O95336	PGLS	ENSP00000252603	m
DNJB6_HUMAN	0.530	5				s
RB11A_HUMAN	0.530	4	P62491	RAB11A	ENSP00000261890	mb
RS24_HUMAN	0.527	5	P62847	RPS24	ENSP00000361435	pr
PGAM2_HUMAN	0.523	5	P15259			m
D6RHH4_HUMAN	0.522	5	D6RHH4	GNB2L1	ENSP00000426101	mb
EPCR_HUMAN	0.521	4	Q9UNN8	PROCR	ENSP00000363601	pm
ENOG_HUMAN	0.520	4				m

EFTU_HUMAN	0.519	6	P49411	TUFM	ENSP00000322439	pr
RAB14_HUMAN	0.515	5	P61106	RAB14	ENSP00000362946	mb
RTN3_HUMAN	0.512	4	O95197	RTN3	ENSP00000367050	pr
DLDH_HUMAN	0.509	5	P09622	DLD	ENSP00000205402	mt
SRSF3_HUMAN	0.504	5	P84103	SRSF3	ENSP00000362820	pr
KIF5A_HUMAN	0.494	6	Q12840	KIF5A	ENSP00000408979	s
GBG12_HUMAN	0.491	5	Q9UBI6	GNG12	ENSP00000360021	pm
GLCM_HUMAN	0.487	4	P04062	GBA	ENSP00000357357	m
TBB4A_HUMAN	0.485	6	P04350	TUBB4A	ENSP00000264071	s
IF2GL_HUMAN	0.484	6	Q2VIR3	EIF2S3L	ENSP00000323063	pr
EPT1_HUMAN	0.483	5	Q9C0D9	EPT1	ENSP00000260585	m
PITH1_HUMAN	0.480	6	Q9GZP4	PITHD1	ENSP00000246151	u
ECH1_HUMAN	0.479	6	Q13011	ECH1	ENSP00000221418	m
POTEF_HUMAN	0.478	5	A5A3E0	POTEF	ENSP00000386786	s
CLIC3_HUMAN	0.471	5	O95833	CLIC3	ENSP00000419378	mb
ODO2_HUMAN	0.469	5	P36957	DLST	ENSP00000335304	u
A8MUD9_HUMAN	0.469	4	A8MUD9	RPL7	ENSP00000379731	pr
CACP_HUMAN	0.463	5	P43155	CRAT	ENSP00000315013	m
MDHC_HUMAN	0.462	5	P40925	MDH1	ENSP00000233114	m
C9J406_HUMAN	0.461	5	C9J406	IMMT	ENSP00000254636	mt
GGCT_HUMAN	0.460	5	O75223	GGCT	ENSP00000275428	m
NDKA_HUMAN	0.459	5	P15531	NME1	ENSP00000376892	m
CDC42_HUMAN	0.452	5	P60953	CDC42	ENSP00000341072	s
H7C0C1_HUMAN	0.451	7	H7C0C1	AP000304.12	ENSP00000394107	u
S10AA_HUMAN	0.447	5	P60903	S100A10	ENSP00000357801	pm
GAN_HUMAN	0.444	5	Q9H2C0	GAN	ENSP00000248272	s
H11_HUMAN	0.435	5	Q02539	HIST1H1A	ENSP00000244573	n
RS26_HUMAN	0.433	4	P62854	RPS26	ENSP00000348849	pr
HNRPD_HUMAN	0.429	6	Q14103	HNRNPD	ENSP00000313199	pr
RL18_HUMAN	0.428	4	Q07020	RPL18	ENSP00000447001	pr
PDIA3_HUMAN	0.424	4	P30101	PDIA3	ENSP00000300289	pr
C9JRH2_HUMAN	0.420	6	C9JRH2	RCC1	ENSP00000402740	n
TMEDA_HUMAN	0.419	4	P49755	TMED10	ENSP00000303145	pm
POTEI_HUMAN	0.417	4	P0CG38	POTEI	ENSP00000392718	s
K2C73_HUMAN	0.415	5	Q86Y46	KRT73	ENSP00000307014	s
BAF_HUMAN	0.407	6	O75531	BANF1	ENSP00000310275	n
K22O_HUMAN	0.406	4	Q01546	KRT76	ENSP00000330101	s
NACA_HUMAN	0.401	4	Q13765	NACA	ENSP00000403817	pr
MYCBP_HUMAN	0.401	5	Q99417	MYCBP	ENSP00000380702	n
H3BTZ8_HUMAN	0.399	4	H3BTZ8	CIAPIN1	ENSP00000457400	m
ATPD_HUMAN	0.399	5	P30049	ATP5D	ENSP00000215375	mt
CNBP1_HUMAN	0.395	4	Q9NSA3	CTNNBIP1	ENSP00000366474	pm
KR261_HUMAN	0.389	4	Q6PEX3			s
H31T_HUMAN	0.388	5	Q16695	HIST3H3	ENSP00000355657	n
GDIR1_HUMAN	0.384	4	P52565	ARHGDIA	ENSP00000269321	mb
ARF5_HUMAN	0.369	4	P84085	ARF5	ENSP00000000233	mb
PO210_HUMAN	0.367	5	Q8TEM1	NUP210	ENSP00000254508	n

ILVBL_HUMAN	0.367	4	A1L0T0			m
LX15B_HUMAN	0.364	4	O15296	ALOX15B	ENSP00000369530	m
FA83H_HUMAN	0.363	4	Q6ZRV2			s
RS16_HUMAN	0.360	4	P62249	RPS16	ENSP00000251453	pr
ASSY_HUMAN	0.359	4	P00966	ASS1	ENSP00000253004	m
PLB1_HUMAN	0.355	4	Q6P1J6			mb
RL23_HUMAN	0.348	5	P62829	RPL23	ENSP00000420311	pr
K1C15_HUMAN	0.347	4	P19012	KRT15	ENSP00000254043	s
APT_HUMAN	0.341	4	P07741	APRT	ENSP00000367615	m
ATPG_HUMAN	0.339	4	P36542	ATP5C1	ENSP00000349142	mt
TACD2_HUMAN	0.332	4	P09758	TACSTD2	ENSP00000360269	pm
RL22_HUMAN	0.331	4	P35268	RPL22	ENSP00000346088	pr
MTCH2_HUMAN	0.330	4	Q9Y6C9	MTCH2	ENSP00000303222	mt
6PGD_HUMAN	0.324	4	P52209	PGD	ENSP00000270776	m
K2C4_HUMAN	0.321	4	P19013			s
F6XWZ1_HUMAN	0.318	4	F6XWZ1	LY6G6D	ENSP00000364984	u
ACOX3_HUMAN	0.315	4	O15254	ACOX3	ENSP00000413994	m
CO1A1_HUMAN	0.306	4	P02452	COL1A1	ENSP00000225964	ex
RS6_HUMAN	0.299	4	P62753			pr
K2C72_HUMAN	0.296	4	Q14CN4	KRT72	ENSP00000441160	s
CTND1_HUMAN	0.283	4	O60716	CTNND1	ENSP00000436543	pm
AAAT_HUMAN	0.278	4	Q15758	SLC1A5	ENSP00000303623	pm
EZRI_HUMAN	0.268	4	P15311	EZR	ENSP00000338934	s
RS8_HUMAN	0.263	4	P62241	RPS8	ENSP00000379888	pr
D6RAJ2_HUMAN	0.253	4	D6RAJ2	SLC27A6	ENSP00000421759	m
