# Supplementary material for: Demonstration of Protein-Based Human Identification Using the Hair Shaft Proteome
Source: PLoS One. 2016 Sep 7;11(9):e0160653. doi: 10.1371/journal.pone.0160653 (PMC5014411; doi:10.1371/journal.pone.0160653)
Supplement: S5 File — Datasets resulting from application of tryptic digests were analyzed using both the Trans Proteomic Pipeline and GPM manager, as outlined in the Supplemental Methods (S1 Methods). Proteomic datasets from a cohort of European–Americans (EA1, n = 51), were analyzed and peptides that contained characterized single amino acid polymorphisms were identified, collated, and summed for each individual. Peptide sequences are included with amino acid polymorphisms indicated in lower case (pept). Single nucleotide polymorphisms that account for the change in amino acid structure are represented in the table by gene name (GN), and dbSNP identifier and allele (rs#_nuc). Multiple alleles occurring within the gene boundary, either through heterozygosity or multiple SNPs are also indicated. The number of observations of alleles, or combination of alleles within a gene boundary, are recoded for both the European (EUR; n = 379) and African (AFR; n = 246) populations (1000 Genomes Project; 1000genomes.org). If a SAP-containing peptide was identified in any of the proteomic datasets associated with an individual, this was indicated by a "1" in the matrix. False positives, identified by genotyping have been removed. A maximum of 1 observation of allele, or combination of alleles, occurs per gene. (PDF) [file pone.0160653.s008.pdf]

GN	rs#_nuc	pept	Allele count EUR (n = 379)	Allele count AFR (n = 246)	Subject EA2 1	3	4	5	6	7	8	12	13	14	15	16	17	18	19
BLMH	rs1050565_C	HVPEEVLAVLEQEPIvLPAWDPMGALA-	213	93															
BLMH	rs1050565_T	HVPEEVLAVLEQEPIILPAWDPMGALA-	335	228	1	1	1			1		1	1	1		1		1	
BLMH	rs1050565_C_T	HVPEEVLAVLEQEPIvLPAWDPMGALA-/HVPEEVLAVLEQEPIILPAWDPMGALA-	169	75				1	1								1		
DSP	rs6929069_A	GqSEADSDKNATILELR	102	158										1					
DSP	rs6929069_G	GR/SEADSDKNATILELR	373	211				1											
ECHS1	rs1049951_A	iFEEDPAVGAIVLTGGDK	377	185						1				1		1	1	1	
GSDMA	rs3894194_A	QLNPqGDLTPLDSLIDFK	264	109															
GSDMA	rs3894194_G	/GDLTPLDSLIDFK	308	225	1		1	1				1				1		1	
GSDMA	rs56030650_A	GHEVnLEALPK	297	220															
GSDMA	rs56030650_C	GHEVTLEALPK	265	117															
GSDMA	rs7212938_G	ALETVQER	277	56															
GSDMA	rs7212938_T	ALETIQER	296	241															
GSDMA	rs3894194_A/rs56030650_A	QLNPqGDLTPLDSLIDFK/GHEVnLEALPK	242	102						1									
GSTP1	rs1695_G	YvSLIYTNYEAGKDDYVK	206	175				1		1				1		1			
GSTP1	rs1695_A	YISLIYTNYEAGKDDYVK	343	203	1													1	
GSTP1	rs1695_A_G	YISLIYTNYEAGKDDYVK /YvSLIYTNYEAGKDDYVK	170	132			1												
HEXB	rs10805890_G	GILVDTSR	100	7															
HEXB	rs10805890_A	GILIDTSR	368	243				1									1		
JUP	rs143043662_T	NEGTATYAAAiLFR	5	0															
JUP	rs143043662_C	NEGTATYAAAVLFR	379	246															1
JUP	rs41283425_T	SAIVHLINYQDDAELAThALPELTK	46	0															
JUP	rs41283425_C	SAIVHLINYQDDAELATR	379	246	1	1				1		1	1	1	1				
JUP	rs143043662_T/rs41283425_T_C	NEGTATYAAAVLFR / SAIVHLINYQDDAELAThALPELTK / SAIVHLINYQDDAELATR	46	0			1												
JUP	rs143043662_C / rs41283425_C	NEGTATYAAAVLFR / SAIVHLINYQDDAELATR	379	246				1									1	1	
JUP	rs143043662_T/ rs41283425_C	NEGTATYAAAiLFR / SAIVHLINYQDDAELATR	5	0					1										
JUP	rs41283425_T_C	SAIVHLINYQDDAELAThALPELTK / SAIVHLINYQDDAELATR	5	0												1			
KRT1	rs17678945_A	NKLNDLEDALQQsKEDLAR	16	0															
KRT1	rs17678945_C	NKLNDLEDALQQAKEDLAR	379	246										1		1	1		1
KRT1	rs17678945_A_C	NKLNDLEDALQQsKEDLAR / NKLNDLEDALQQAKEDLAR				1													
KRT31	rs112544857_A	SQYEvLVETNR	19	1													1		
KRT31	rs6503627_A	DNvELENLIR or QLERDNvELENLIR	64	16				1				1							
KRT32	rs72830046_T	CQYEAMVEANhR	174	24															
KRT32	rs72830046_C	CQYEAMVEANRR	356	246															
KRT32	rs2071563_A	LEGEINmYR	259	163									1						
KRT32	rs2071563_G	LEGEINTYR	315	196				1			1	1					1		
KRT32	rs2071561_T	ADLEAQVEyLK	276	104															
KRT32	rs2071563_G / rs2071561_T	LEGEINTYR / ADLEAQVEyLK	276	101	1		1			1				1	1	1		1	1
KRT32	rs72830046_C / rs2071563_A	CQYEAMVEANRR / LEGEINmYR	155	163		1													
KRT32	rs2071563_A_G/rs2071561_T	LEGEINTYR / ADLEAQVEyLK							1										
KRT33A	rs12937519_A	QVVSSSEQLQSYQvEIIELR	169	147				1		1	1	1		1		1			1
KRT34	rs2239710_T	SQYEALVEINR	218	22		1			1	1	1	1	1	1	1	1		1	1
KRT35	rs743686_A	VSAMYSSSSCKLPSLSPVAR	266	111															
KRT35	rs743686_G	VSAMYSSSpCKLPSLSPVAR	295	228													1		
KRT35	rs12451652_T	TNySPRPICVPCPGGRF	116	33															
KRT35	rs12451652_C	TNCSPRPICVPCPGGRF	363	243															
KRT35	rs2071601_C	TNCSaRPICVPCPGGRF	295	220															
KRT35	rs2071601_G	TNCSPRPICVPCPGGRF	250	136															
KRT35	rs743686_A_G / rs12451652_C / rs2071601_G	VSAMYSSSSCKLPSLSPVAR / VSAMYSSSpCKLPSLSPVAR/TNCSPRPICVPCPGGRF	182	92		1													
KRT37	rs9910204_A	TSFYSTSSCPLcCTMAPGAR	180	73															
KRT37	rs9910204_C	TSFYSTSSCPLGCTMAPGAR	354	237															
KRT40	rs150812789_G	TASALEIELQAQQSLTESLECTVAETEAQYSSQLAQIQr / LIDNLENQLAEIR	164	27		1	1	1									1	1	
KRT40	rs150812789_A	TASALEIELQAQQSLTESLECTVAETEAQYSSQLAQIQCLIDNLENQLAEIR	353	244															
KRT81	rs6580873_A	LYEEEILILQSHISDTSVVVK	107	11	1											1			
KRT81	rs2071588_G	GLTGGFGSHSVCr	212	30		1	1				1				1				
KRT81	rs6580873_A/rs2071588_G	LYEEEILILQSHISDTSVVVK / GLTGGFGSHSVCr	33	1				1											
KRT83	rs2852464_C	DLNMDCmVAEIK	235	179															
KRT83	rs2852464_G	DLNMDCIVAEIK	327	189	1	1	1	1		1	1	1	1		1	1	1		
KRT85	rs61630004_T	IAVGGFRAGSCGhSFGYR / AGSCGhSFGY	35	10															
KRT85	rs61630004_C	IAVGGFRAGSCGR / AGSCGR	379	246				1											
LRRC15	rs13070515_A	ELSIGIFGPMPNLR	150	38															
LRRC15	rs13070515_G	ELSPGIFGPMPNLR	358	242			1						1	1		1			1
LRRC15	rs13060627_T	LYLSNNHISQLPPSIFMQLPQLNR	168	92															
LRRC15	rs13060627_C	LYLSNNHISQLPPSVFMQLPQLNR	355	235													1		
LRRC15	rs13070515_A_G / rs13060627_T	ELSIGIFGPMPNLR / ELSPGIFGPMPNLR / LYLSNNHISQLPPSiFMQLPQLNR	128	34				1											
LRRC15	rs13070515_G/rs13060627_C	ELSPGIFGPMPNLR / LYLSNNHISQLPPSVFMQLPQLNR	355	235					1	1								1	
S100A3	rs36022742_T	AkPLEQAVAAIVCTFQEYAGR	32	145															
S100A3	rs36022742_C	ARPLEQAVAAIVCTFQEYAGR	378	211			1	1	1	1	1	1	1	1	1	1	1	1	1
S100A3	rs36022742_T_C	AkPLEQAVAAIVCTFQEYAGR / ARPLEQAVAAIVCTFQEYAGR	31	110		1													
SERPINB5	rs1455555_A	GVALSNVIHK	284	237															
SERPINB5	rs1455555_G	GVALSNVvHK	277	73						1				1					
TGM3	rs214803_C	AALGVQSINWQTAFNR	120	206															
TGM3	rs214803_A	AALGVQSINWQkAFNR	372	171		1		1	1	1							1		
TGM3	rs214803_C_A	AALGVQSINWQTAFNR / AALGVQSINWQkAFNR	114	131			1								1	1		1	
