# Supplementary material for: Demonstration of Protein-Based Human Identification Using the Hair Shaft Proteome
Source: PLoS One. 2016 Sep 7;11(9):e0160653. doi: 10.1371/journal.pone.0160653 (PMC5014411; doi:10.1371/journal.pone.0160653)
Supplement: S6 File — Datasets resulting from application of tryptic digests were analyzed using both the Trans Proteomic Pipeline and GPM manager, as outlined in the Supplemental Methods (S1 Methods). A cohort of European–Americans (EA2, n = 15) were analyzed and peptides that contained characterized single amino acid polymorphisms were identified, collated, and summed for each individual. Peptide sequences are included with amino acid polymorphisms indicated in lower case (pept). Single nucleotide polymorphisms that account for the change in amino acid structure are represented in the table by gene name (GN), and dbSNP identifier and allele (rs#_nuc). Multiple alleles occurring within the gene boundary, either through heterozygosity or multiple SNPs are also indicated. The number of observations of alleles, or combination of alleles within a gene boundary, are recoded for both the European (EUR; n = 379) and African (AFR; n = 246) populations (1000 Genomes Project; 1000genomes.org). If a SAP-containing peptide was identified in any of the proteomic datasets associated with an individual, this was indicated by a "1" in the matrix. False positives, identified by genotyping have been removed. A maximum of 1 observation of allele, or combination of alleles, occurs per gene. (PDF) [file pone.0160653.s009.pdf]

GN	EUR (n=379)	AFR (n=246)	rs#_nuc	pept	AA A1 comb	AA A2 comb	AA A3 comb	AA A4 comb	AA A5 comb	Kenyan 1 D1	Kenyan 2 D2	Kenyan 3 D3	Kenyan 4 D4	Kenyan 5 D5B
JUP	46	5	RS41283425	SAIVHLINYQDDAELAThALPELTK										
JUP	379	246	RS41283425	SAIVHLINYQDDAELATR	1	1	1		1			1		
KRT31	64	16	rs6503627	DNvELENLIR									1	
KRT31	19	1	rs112544857	SQYEvLVETNR										
KRT31	0	1	rs146247884	QNQEYQmLLDVR								1		
KRT32	276	104	rs2071561_T	ADLEAQVEyLK										
KRT32	315	196	rs2071563_G	LEGEINTYR				1			1			
KRT32	184	133	rs2071563_A	LEGEINmYR										
KRT32	120	83	rs2071563_G_A	LEGEINTYR/LEGEINmYR										
KRT32	356	246	rs72830046_C	CQYEAMVEANRR										
KRT32	174	24	rs72830046_T	CQYEAMVEANhR										
KRT32	151	24	rs72830046_C_T	CQYEAMVEANRR/CQYEAMVEANhR										
KRT32	275	101	rs2071561_T/rs2071563_G	ADLEAQVEyLK/LEGEINTYR										
KRT32	161	47	rs2071561_T/rs2071563_G_A	ADLEAQVEyLK/LEGEINTYR/LEGEINmYR								1		
KRT32	161	50	rs2071561_T/rs2071563_A	ADLEAQVEyLK/LEGEINmYR		1								
KRT32	100	6	rs2071563_G_A/	LEGEINTYR/LEGEINmYR/CQYEAMVEANhR	1									
KRT32	147	11	rs2071561_T/rs2071563_G/rs72830046_T	ADLEAQVEyLK/LEGEINTYR/CQYEAMVEANhR			1							
KRT32	292	196	rs2071563_G/rs72830046_C	LEGEINTYR/CQYEAMVEANRR					1	1				
KRT32	172	24	rs2071563_G/rs72830046_T	LEGEINTYR/CQYEAMVEANhR										
KRT33A	169	147	rs12937519_A	QVVSSSEQLQSYQvEIIELR			1		1	1	1	1	1	1
KRT33B	0	5	rs114488848_G	TINALEIELQAQHNLR							1			
KRT34	218	22	rs2239710_T	SQYEALVEINR			1					1		
KRT35	295	228	RS743686_G	VSAMYSSSpCKLPSLSPVAR		1	1	1		1				
KRT35	266	111	RS743686_A	VSAMYSSSSCKLPSLSPVAR								1		
KRT35	182	93	RS743686_G_A	VSAMYSSSpCKLPSLSPVAR/VSAMYSSSSCKLPSLSPVAR										
KRT35	379	246	rs138303882_G	YETEVSLRQLVESDINGLR								1		
KRT35	0	2	rs138303882_A	YETEVSLwQLVESDINGLR										
KRT35	0	1	RS743686_A/rs138303882_G_A	VSAMYSSSSCKLPSLSPVAR/YETEVSLRQLVESDINGLR /YETEVSLwQLVESDINGLR					1					
KRT35	0	2	RS743686_A/rs138303882_G	VSAMYSSSpCKLPSLSPVAR/YETEVSLwQLVESDINGLR							1			
KRT37	354	237	rs9910204_C	TSFYSTSSCPLGCTMAPGAR										1
KRT37	180	73	rs9910204_A	TSFYSTSSCPLcCTMAPGAR										
KRT40	353	244	Rs150812789_A	TASALEIELQAQQSLTESLECTVAETEAQYSSQLAQIQCLIDNLENQLAEIR										
KRT40	164	27	Rs150812789_G	TASALEIELQAQQSLTESLECTVAETEAQYSSQLAQIQr or /LIDNLENQLAEIR										
KRT40	245	228	rs2010027_C	NHEEEVNLLREQLGDR										
KRT40	309	117	rs2010027_T	NHEEEVNLLhEQLGDR				1						
KRT40	164	27	Rs150812789_G/rs2010027_C	/LIDNLENQLAEIR; NHEEEVNLLREQLGDR								1		
KRT81	107	11	rs6580873_A	LYEEEILIlQSHISDTSVVVK										
KRT81	212	30	rs2071588_G	GLTGGFGSHSVCr								1		
KRT81	379	232	rs79897879_T	FSCISACGPRPGR									1	1
KRT81	3	103	rs79897879_G	FrCISACGPRPGR										
KRT81	1	9	rs2071588_G/rs79897879_G	GLTGGFGSHSVCr; FrCISACGPRPGR			1							
KRT82	278	140	rs2658658_G	GAFLYEPCGVSTPVLSTGVLR			1							
KRT82	302	216	rs2658658_A	GAFLYEPCGVSmPVLSTGVLR				1						
KRT83	327	189	rs2852464_G	DLNMDCIVAEIK or	1			1			1			
KRT83	235	179	rs2852464_C	DLNMDCmVAEIK										
KRT83	183	122	rs2852464_G_A	DLNMDCIVAEIK/DLNMDCmVAEIK			1		1	1		1		1
KRT83	0	19	rs140635030_T	LEAAVAQSEQQsEAALSDAR										
KRT83	0	10	rs2852464_C_G/rs140635030_T	DLNMDCIVAEIK/DLNMDCmVAEIK/LEAAVAQSEQQsEAALSDAR		1								
KRT84	357	245	RS951773_G	QLrEYQELMNAKLGLDIEIATYR	1	1	1	1	1	1		1		
KRT84	182	41	RS951773_A	CEYQELMNAKLGLDIEIATYR										
KRT86	0	16	rs139895699_T	TKEEINELNcMIQR		1								
KRTAP3-2	377	167	rs9897046_T	MDCCASRSCSVPTGPATTICSSDKSCR			1	1	1		1	1	1	
LRRC15	358	242	rs13070515_G	ELSPGIFGPMPNLR				1						
LRRC15	150	38	rs13070515_A	ELSlGIFGPMPNLR										
LRRC15	129	34	rs13070515_G_A	ELSPGIFGPMPNLR / ELSlGIFGPMPNLR										
LRRC15	355	235	rs13060627_C	LYLSNNHISQLPPSVFMQLPQLNR		1								1
LRRC15	168	92	rs13060627_A	LYLSNNHISQLPPSlFMQLPQLNR										
S100A3	378	211	RS36022742_C	ARPLEQAVAAIVCTFQEYAGR		1	1	1		1				
S100A3	32	145	RS36022742_T	AkPLEQAVAAIVCTFQEYAGR					1			1		1
S100A3	31	110	RS36022742_C_T	ARPLEQAVAAIVCTFQEYAGR/AkPLEQAVAAIVCTFQEYAGR									1	
S100A3	379	246	rs116208483_G	FMSVLDTNKDCEVDFVEYVR	1	1	1	1	1	1		1	1	1
S100A3	27	0	rs116208483_C	FMSVvDTNKDCEVDFVEYVR										
TCHH	0	16	rs2515663	TVDLILELLDL										
TCHH	379	245		TVDLILELLDr							1			
