# Supplementary material for: Demonstration of Protein-Based Human Identification Using the Hair Shaft Proteome
Source: PLoS One. 2016 Sep 7;11(9):e0160653. doi: 10.1371/journal.pone.0160653 (PMC5014411; doi:10.1371/journal.pone.0160653)
Supplement: S7 File — Datasets resulting from application of tryptic digests were analyzed using both the Trans Proteomic Pipeline and GPM manager, as outlined in the Supplemental Methods (S1 Methods). A cohort of 5 African-American subjects, and 5 Kenyan subjects (S5)[39], were analyzed and peptides that contained characterized single amino acid polymorphisms were identified, collated, and summed for each individual. Peptide sequences are included with amino acid polymorphisms indicated in lower case (pept). Single nucleotide polymorphisms that account for the change in amino acid structure are represented in the table by gene name (GN), and dbSNP identifier and allele (rs#_nuc). Multiple alleles occurring within the gene boundary, either through heterozygosity or multiple SNPs are also indicated. The number of observations of alleles, or combination of alleles within a gene boundary, are recoded for both the European (EUR; n = 379) and African (AFR; n = 246) populations (1000 Genomes Project; 1000genomes.org). If a SAP-containing peptide was identified in any of the proteomic datasets associated with an individual, this was indicated by a "1" in the matrix. False positives, identified by genotyping have been removed. A maximum of 1 observation of allele, or combination of alleles, occurs per gene. (PDF) [file pone.0160653.s010.pdf]

Publically available proteomic datasets

MS	Treatment	Subject	GPM accession #
Thermo Hybr	II	C0001	GPM32100009022
Thermo Hybr	II	**C0001**	GPM32100009016
Thermo Hybr	II	**C0003**	GPM32100009096
Thermo Hybr	II	**C0004**	GPM32100010108
Thermo Hybr	II	**C0005**	GPM32100008962
Thermo Hybr	II	**C0005**	GPM32100008970
Thermo Hybr	II	**C0006**	GPM32100009512
Thermo Hybr	II	**C0007**	GPM32100010750
Thermo Hybr	II	**C0008**	GPM32100009533
Thermo Hybr	II	**C0009**	GPM32100009569
Thermo Hybr	II	**C0013**	GPM32100009570
Thermo Hybr	II	**C0014**	GPM32100009571
Thermo Hybr	II	**C0015**	GPM32100009572
Thermo Hybr	II	**C0016**	GPM32100009575
Thermo Hybr	II	**C0016**	GPM32100009580
Thermo Hybr	II	**C0017**	GPM32100009014
Thermo Hybr	II	**C0018**	GPM32100010850
Thermo Hybr	II	**C0019**	GPM32100009152
Bruker maXis	IIIA	**C0019**	GPM32100032293
Bruker maXis	IIIA	**C0018**	GPM32100032294
Bruker maXis	IIIA	**C0017**	GPM32100032295
Bruker maXis	IIIA	**C0016**	GPM32100032296
Bruker maXis	IIIA	**C0014**	GPM32100032298
Bruker maXis	IIIA	**C0015**	GPM32100032297
Bruker maXis	IIIB	**C0018**	GPM32100011243
Bruker maXis	IIIB	**C0013**	GPM32100011264
Bruker maXis	IIIB	**C0015**	GPM32100032299
Bruker maXis	IIIB	**C0003**	GPM32100032300
Bruker maXis	IIIB	**C0006**	GPM32100032311
Bruker maXis	IIIB	**C0012**	GPM32100032312
Bruker maXis	IIIB	**C0017**	GPM32100032316
Bruker maXis	IIIB	**C0019**	GPM32100032313
Bruker maXis	IIIB	**C0001**	GPM32100032317
Bruker maXis	IIIB	**C0014**	GPM32100032318
Bruker maXis	IIIA	**C0016**	GPM32100032319
Bruker maXis	II	**C0014**	GPM32100032323
Bruker maXis	II	**C0007**	GPM32100010859

Bruker maXis II	**C0008**	GPM32100010971
Bruker maXis II	**C0007**	GPM32100011141
Bruker maXis II	**C0004**	GPM32100032324
Bruker Maxis II	**C0005**	GPM32100011285
Bruker Maxis II	**C0005**	GPM32100011294
Bruker Maxis II	**C0005**	GPM32100011356
Agilent qToF I	L1.001.1C	GPM32100011293
Agilent qToF I	L1.002.1C	GPM32100011454
Agilent qToF I	L1.003.1C	GPM32100011452
Agilent qToF I	L1.004.1C	GPM32100011494
Agilent qToF I	L1.005.1C	GPM32100011511
Agilent qToF I	C0003	GPM32100011519
Agilent qToF I	L1.006.1C	GPM32100011512
Agilent qToF I	L1.007.1C	GPM32100011520
Agilent qToF I	L1.008.1C	GPM32100011694
Agilent qToF I	L1.009.1C	GPM32100011609
Agilent qToF I	L1.012.1C	GPM32100015905
Agilent qToF I	C0003	GPM32100015967
Agilent qToF I	L1.013.1C	GPM32100016095
Agilent qToF I	L1.014.1C	GPM32100016121
Agilent qToF I	L1.015.1C	GPM32100016639
Agilent qToF I	L1.016.1C	GPM32100016637
Agilent qToF I	C0005.1	GPM32100016642
Agilent qToF I	C0017.1	GPM32100016696
Agilent qToF I	L1.017.1C	GPM32100016779
Agilent qToF I	L1.019.1C	GPM32100016876
Agilent qToF I	L1.021.1C	GPM32100016939
Agilent qToF I	L1.026.1C	GPM32100016941
Agilent qToF I	C0003	GPM32100016949
Agilent qToF I	L1.029.1C	GPM32100016942
Agilent qToF I	L1.030.1C	GPM32100017523
Agilent qToF I	L1.031.1C	GPM32100017613
Agilent qToF I	L1.032.1C	GPM32100017585
Agilent qToF I	L1.033.1C	GPM32100017609
Agilent qToF I	L1.037.1C	GPM32100016952
Agilent qToF I	L1.039.1C	GPM32100016953
Agilent qToF I	L1.040.1C	GPM32100027585
Agilent qToF I	L1.041.1C	GPM32100027631
Agilent qToF I	L1.042.1C	GPM32100027657
Agilent qToF I	L1.046.1C	GPM32100027678

Agilent qToF I	L1.048.1C	GPM32100027679
Agilent qToF I	L1.047.1C	GPM32100027683
Agilent qToF I	L1.050.1C	GPM32100031254
Agilent qToF I	L1.051.1C	GPM32100031313
Agilent qToF I	C0003	GPM32100031986
Agilent qToF I	L1.020.1C	GPM32100031306
Agilent qToF I	L1.023.1C	GPM32100031318
Agilent qToF I	L1.028.1C	GPM32100031319
Agilent qToF I	L1.035.1C	GPM32100031330
Agilent qToF I	L1.036.1C	GPM32100031321
Agilent qToF I	C0003	GPM32100031329
Agilent qToF I	L1.038.1C	GPM32100031323
Agilent qToF I	L1.044.1C	GPM32100031334
Agilent qToF I	L1.045.1C	GPM32100031349
Agilent qToF I	L1.049.1C	GPM32100031577
Agilent qToF I	L1.052.1C	GPM32100031574
Agilent qToF I	L1.053.1C	GPM32100031768
Agilent qToF I	L1.054.1C	GPM32100031833
Agilent qToF I	L1.057.1C	GPM32100031830
Agilent qToF I	L1.058.1C	GPM32100031840
Agilent qToF I	L1.059.1C	GPM32100031838
Agilent qToF I	L1.060.1C	GPM32100031941
Agilent qToF I	L1.001.2C	GPM32100031957
Agilent qToF I	L1.056.2C	GPM32100031959
Agilent qToF I	L1.055.2C	GPM32100031958
Agilent qToF I	C0003	GPM32100031975
