# Supplementary material for: Demonstration of Protein-Based Human Identification Using the Hair Shaft Proteome
Source: PLoS One. 2016 Sep 7;11(9):e0160653. doi: 10.1371/journal.pone.0160653 (PMC5014411; doi:10.1371/journal.pone.0160653)
Supplement: S1 Methods — Detailed protocols for data acquisition on a Thermo Hybrid FT/LTQ, a Bruker maXis Impact qToF, and Agilent 1290/Agilent 6530 Accurate-Mass Q-ToF are outlined. A description of the discovery process of genetically variant peptides is included, particularly the creation and characterization of a custom reference protein variant database (RefSeq_Protein_Variant_Database.txt; https://zenodo.org/record/58223; DOI: 10.5281/zenodo.58223). (DOCX) [file pone.0160653.s011.docx]

**Supporting Information**

**S1 Methods**

**Physical and Biochemical Treatment of Hair Shafts**

Cranial hair from the European-American cohort 1 (EA1), used in Figures 1 and 2, were treated using Treatment I. Hair from the European-American cohort 2 (EA2) used in Figures 1 to 4 was treated as described below in Treatments II and III. African-American and Kenyan samples were processed according to the literature[1]. Archaeological samples in Figures 4 and 5 were treated as outlined in Treatments II and IIIC.

Treatment I*.* Cranial hair shafts (10.5 ± 0.5 mg) were placed in a milling vial containing 2.8 mm ceramic beads (Omni-International Inc.); 200 µL of buffer containing 1.5 M urea, 0.125 M DTT, 0.1 M ammonium bicarbonate, and 0.01% (w/v) Protease-Max (Promega Inc.) was added and milled for 3 min at 4.5 m/s. The mixture was rotated gently overnight, 50 µL of 1M iodoacetamide added, and milled again for 3 min at 4.5 m/s. After 30 min of gentle rotation in half light, 200 µl of a mixture containing 0.08 M DTT, 0.04 M ammonium bicarbonate, 0.0125% (w/v) Protease-Max, and 0.5 µg/µL Trypsin-TPCK (Worthington Enzymes Inc.) was added and gently rotated overnight at RT (16 to 24 h). Vials were centrifuged at > 10,000 g for 15 min. An aliquot of supernatant (400 µL) was added to a 1.5 ml “LoBind” Eppendorf microcentrifuge tube, centrifuged at > 10,000 g for 15 min, and 200 µL of supernatant was added to an Agilent sample vial with inactivated glass insert.

Treatment II. At least 10 mg of hair was washed twice for 2 h in 50 ml of 10% methanol, followed by at least a 2 h wash in 50 ml water (Ω>18 mOhm, Millipore Inc.) on a rotating vertical turntable. Washed hair was dried and ground on a glass plate using the exterior of a glass Erlenmeyer flask. Collected powder was treated with 50 mM ammonium bicarbonate containing 1% (w/v) Protease-Max and 30 mM DTT. The ground hair was centrifuged and the insoluble fraction treated with 20 µg TPCK – treated trypsin (Worthington Enzymes Inc.) in 50 mM ammonium bicarbonate containing 1% (w/v) Protease-Max and 30 mM DTT, at 37˚C overnight. The supernatant was diluted 150-fold in Buffer A and applied to mass spectrometry, either on a Thermo hybrid FT/LTQ (University of Utah, Proteomics Core Facility) or a Bruker qToF (maXis Impact, Montana State University).

Treatment III*.* Hair (10 mg) was washed in 10 ml of 20% methanol for 2 h, and 2 h to overnight in water (Ω>18 mOhm, Millipore Inc.). Hair was ground in 200 µL of 50 mM ammonium bicarbonate using ceramic bead vials (diameter = 2.8 mm, Omni-International, cat# 19-628) at a speed of 7.5 m/s, for 3 sec 10 times with a dwell time of 30 sec DTT (10 µL of 1M) was added, followed 3 h later by 40 µL of 0.5M iodoacetamide, which was incubated in the dark at room temperature. After 2 h, 80 µL of 0.2% Protease-Max (w/v) in 50 mM ammonium bicarbonate containing 32 µg trypsin (TPCK-treated sequencing grade, Worthington Biochemical Corp.) was added and incubated by shaking overnight at room temperature. The incubations were diluted with 500 µL water, centrifuged, and a 200 µL aliquot of supernatant applied to a Bruker qToF (Treatment III A).

The remaining preparation (about 600 µL) was vortexed, and a 100 µL slurry was treated by vortexing with 400 µL of acetone and leaving at -20˚C for 15 min. The preparation was vortexed, centrifuged for 4 min at 15,000 g, and the organic phase removed. The aqueous phase was then dried under nitrogen gas, and 20 µL of 20 mM ammonium bicarbonate containing 100 mM DTT, 6M urea, and 0.01% Protease-Max added. After overnight incubation, 5 µl of 0.8M iodoacetamide was added, mixed, and incubated for 60 min at 25˚C, followed by addition of 50 µL of 50 mM ammonium bicarbonate containing 5 µg Trypsin-TPCK. After overnight incubation the sample was centrifuged at 15,000 g for 5 min and the supernatant applied to the Bruker qToF (Treatment III B).

Alternatively, 1 mg of acetone treated hair was resuspended in 100 µl of 100 mM ammonium bicarbonate containing, 0.05% (w/v) sodium deoxycholate and 100 mM dithiothreitol. After 2 h at room temperature this was diluted with 100 µL of 200 mM iodoacetamide, incubated in the dark for 30 min, before adding 20 µL of 2 µg trypsin-TPCK in 0.1% formic acid. After an overnight incubation at room temperature the incubation was acidified with formic acid until the pH was less than 5 and the resulting precipitate was removed by centrifugation at 15,000 g for 5 min. The resulting supernatant was applied to a Bruker qToF (Treatment III C).

**Data Acquisition**

Figures 1 and 2 were generated from data collected on an Agilent 6530 instrument (Protocol I). Data for African-American and Kenyan samples in Figure 2 were obtained as described in Laatsch et al.[2]. Processed hair shaft protein proteomic datasets in Figure 3 were acquired using either an LTQ/FT hybrid or Bruker maXis qToF. Data acquired for Figures 4 and 5 were obtained using an LTQ/FT hybrid and Bruker qToF; the archaeological samples were only applied to the Bruker qToF using the protocol described above (*Treatment III B and C*).

Mass Spectrometry: Thermo Hybrid FT/LTQ (University of Utah, Proteomics Core Facility)

Tryptic preparations of hair shaft proteins were analyzed by LC/MS/MS analysis using an ESI Ion-Trap/ FTMS hybrid mass spectrometer (LTQ-FT, ThermoElectron). An aliquot of 0.5–20% of each sample was injected onto a nano-LC column (75 mm id x 10 cm, Atlantis dC18 RP, 3 mm particle size, Waters Corporation, Milford, MA.) using a nano-LC system (NanoLC 2D, Eksigent Technologies) with a gradient of 5 to 96% solvent B in 78 minutes (solvent B: 100% acetonitrile with 0.1% formic acid; solvent A: 100% water with 0.1% formic acid) at 350 nL/min. Primary mass spectra were acquired in the FTMS portion of the instrument and MS/MS sequence information was collected in the linear ion trap using CID. Primary mass spectra were acquired with typically better than 3 ppm mass accuracy; CID frag- mentation spectra were acquired with less than 0.3 Da mass error.

Mass Spectrometry: Bruker maXis Impact qToF (Montana State University Mass Spectrometry, Proteomics & Metabolomics Facility)

LC-MS/MS analysis was performed using a Bruker maXis Ultra High Resolution TOF (Bruker Corp.) mass spectrometer fitted to an UltiMate^®^ 3000 Nano LC (Thermo Scientific Inc.). Samples were trapped and desalted on the Zorbax 300SB-C18 Agilent HPLC-Chip enrichment column (40 nl volume) in 5% acetonitrile 0.1% formic acid delivered by an auxillary CapLC pump at 4 μl/min. The peptides were then eluted and loaded onto the analytical capillary column (43 mm x 75 μm ID, also packed with 5 μm Zorbax 300SB-C18 particles) connected in-line to the mass spectrometer with a flow of 600 nl/min. Peptides experienced a 5 to 90% acetonitrile gradient over 120 min. Data-dependent acquisition of collision-induced dissociation tandem mass spectrometry (MS/MS) was utilized with the following parameters: m/z range of 200 to 2,200 at 24,300 m/z-s. Raw data files were then exported for data analysis.

Mass Spectrometry: Agilent 1290/Agilent 6530 Accurate-Mass Q-TOF (Forensics Science Center, Lawrence Livermore National Laboratory)

Data acquisition was achieved using an Agilent 1290 liquid chromatography system in line with an Agilent 6530 Accurate-Mass Q-TOF, Dual Agilent Jet Stream ESI Ion Source. The LC separation was achieved using gradient elution on an AdvanceBio Peptide Map C18 column and guard column (Agilent, Santa Clara, CA). Peptides were resolved using a 120 min gradient of 5% to 50% acetonitrile in 0.1% formic acid at a flow rate of 200 µL per min and 50 °C. The column was washed in 95% acetonitrile in 0.1% formic acid for 5 minutes. Post-separation column clean-up and re-equilibration occurred through 4 x 1 minute gradients of 5 to 95% and 95% to 5% acetonitrile in 0.1% formic acid, followed by 15 minutes in 5% acetonitrile in 0.1% formic acid. The injection needle was repeatedly washed with a 1:1 mix of water and isopropanol between runs.

Automatic MSMS parameters were chosen that maximized the number of unique peptides identified in a standard quality control hair sample. Data acquisition occurred with a mass range of 100 to 3000 m/z, a scan rate of 6Hz for the primary MS scan, 1.5 Hz for the secondary MS/MS scan, with a maximum of 5 MS/MS scans being initiated between primary scans. Collision energy was set as a linear function with a slope of 3 and offset of 2 for all charge states. Precursor masses were automatically selected based on total ion current with a target of 25,000 counts per spectrum, with a threshold of 1000 counts, with an abundance based scan speed activated. Precursors were selected based on charge state followed by abundance, with order of preference being z = 2, 3, 1, >3. The purity stringency was 75% with a cut-off of 30%. Active exclusion was activated after 6 spectra, and released after 30 s. The source parameters included a gas temperature of 320°C at 8 L/min and 27 psig. The sheath gas temperature was 380°C at 12 L/min. The source voltages were; 3750 Vcap, 500 V nozzle voltage, and 150V, 65 V, and 750 V for the skimmer, fragmentor and octopole (RF) respectively. Reference masses (m/z = 121.05090 and 922.00980) were run simultaneously in alternate scans.

Processed hair samples were run in batches. Before each batch, the mass spectrometer was tuned and calibrated according to the manufacturer’s instructions. To avoid cross-contamination, two blanks were run at the start of the batch and one was run before each sample. The blanks were run with a faster gradient (ramp 5% to 50% in 20 min rather than 120 min) than the sample runs, but the rest of the elution program (including the column clean-up steps) was identical to the sample runs. Blank runs were used to confirm that this clean-up protocol was sufficient to remove residual peptides from the separation column. The quality control sample was run at the start of the batch and after every five samples. Occasionally (at least once per batch of samples) a blank was run in place of a sample to confirm that no sample carry-over was affecting the data.

**Identification of nsSNP-Containing Peptides**

Proteomic datasets from the Bruker maXis Impact qToF and the Agilent 6530 Accurate-Mass Q-TOF, were converted to the mass spectrometry generic format (mgf) and analyzed using two different approaches: by submission to the GPM manager software ([www.thegpm.org](http://www.thegpm.org), release SLEDGEHAMMER (2013.09.01)), or the Petunia Graphic User Interface (TANDEM CYCLONE TPP, download = 2011.12.01.1 - LabKey, Insilicos, ISB). For the Petunia GUI a custom protein reference database was built that contained a reference protein library in FASTA format with redundant sequences included that contained a single amino acid polymorphism in the protein sequence (Supplemental Material). Resulting peptide lists were screened for the presence of single amino acid polymorphisms and genetic and proteomic data collated together for each individual (Supplemental Material). Imputations made through the use of GPM manager or the use of the customized reference database, in either X!Tandem or MASCOT, were compared for redundancy (S2 Table).

Datasets obtained using the Thermo Hybrid FT/LTQ were analyzed using the MASCOT peptide spectra-matching algorithm (software version 2.2.03, Matrix Science, Inc., Boston, MA). Peaklists for database searching were generated for peptide precursor ions (i.e., +1, +2 and/or +3 charge states) and corresponding CID fragmentation data using SEQUEST (BioWorks Browser, revision 3.2, ThermoElectron Corp.) with the default parameters. Resulting DTA files from each application were combined for each species and analyzed using MASCOT (software version 2.2.03, Matrix Science, Inc.). The datasets were searched using the human taxonomy classification within the NCBI database and a customized database containing single amino acid polymorphisms in addition to wild type protein sequences. The following MASCOT search parameters were used in the analysis: tryptic-specific peptides, maximum of three missed cleavages, no fixed modifications, and variable methionine oxidation (+15.994919 Da).

**Creation of Custom Protein Database**

The PBIT database is a unique protein sequence database, developed for the express purpose of defining variant peptides that can then be detected for use in the identification of individuals. This database can be used in conjunction with any mass spectrometry analytical tool such as Xtandem, Sequest, Mascot, and SpectraST. The RefSeq protein database was used as a starting point for the PBIT protein reference database. The RefSeq protein sequence database human.protein.gpff.gz contains all known amino acid (aa) variant information, but is not in a format readily useful as a database for mass spectrometry software engines. From the UCSC ftp site <ftp://ftp.ncbi.nlm.nih.gov/refseq/H_sapiens/mRNA_Prot/>, the file snp137Common.txt.gz, which contains all of the common variants with frequencies >= 1%, was downloaded (<http://genome.ucsc.edu/cgi-bin/hgTables>; Human, assembly: Feb. 2009 (GRCh37/hg19). The human.protein.gpff file contains reference sequences, but not necessarily unique sequences. First, 4817 duplicated sequences were removed from the database. Then, for each sequence, the list of variants was gathered from two sources: the snp137Common.txt.gz file and the ESP 6500 db which contains SNPs, INDELs and coverage data for the ESP 6500 exomes (chromosomes 1-22, X, and Y). File [ESP6500SI-V2-SSA137.dbSNP138-rsIDs.snps_indels.txt.tar.gz](http://evs.gs.washington.edu/evs_bulk_data/ESP6500SI-V2-SSA137.dbSNP138-rsIDs.snps_indels.txt.tar.gz) comes from the ftp site <http://evs.gs.washington.edu/EVS/> at NHLBI. The snp137Common.txt file contains all of the common variants with MAF (minor allele frequency) >= 1%. The ESP6500 database contains data from various collaborators from 6503 samples for European American (EA) and African American (AA) individuals. The ESP 6500 database, with 3.47 million variants, was filtered to pull all variants with either EA or AA MAF >= 0.5%. All unique variants from these two sources were then used to create the variant sequences used in the PBIT database. Each reference sequence was duplicated once, and labeled the same as the reference sequence with the exception of the addition of the “.v1” string at the end of the NM number. The position of the variants in the sequence and their individual proximity was not a factor. If, however, two or more variants occurred in the same position, the first variant in the list at that position was used. Stop variants were not used. The final PBIT database contains a reference sequence and variant sequence, if one or more variants exist in the sequence, for each protein sequence. There are 34,383 NP_ loci, 1,833 XP_ loci, and 13 YP_ loci in human.protein.gpff for a total 36,229 unique locus names for homo sapiens. The NM numbers are an identifier that differentiates between multiple assignments to the same gene, and are used in the PBIT database as a way of identifying sequence. Large proteins presumably not involved in hair were removed from the file to facilitate run time (Gene Names = TTN, MUC16, OBSCN, NEB, MUC19, AHNAK, AHNAK2, MUC5B, MUC4, FCGBP, MUC12, LOC100289142, USH2A, MUC2, SSPO, HYDIN, RYR1). The database was formatted in FASTA format.

**Custom database file summary**

A characterization of the database includes: 37% of proteins do not have variants > 0.5%, 91% of proteins have 0 or 1 variants > 0.5%, and 99% of proteins have 0, 1 or 2 variants > 0.5%. There are 36,229 protein sequences represented by a unique NM number; 350 protein sequences have at least one peptide with three variants, 169 protein sequences have at least one peptide with four or more variants. The number of variants in the ESP db with a MAF > 0.5% was 106,000 variants. The unique list of these variants is 67,250. Of these, there are 31,230 which that were not identified in the snp137common.txt file. There are 13,585,949 rs numbers in the snp137Common.txt file and 31,230 NEW ESPdb rs numbers that have a frequency of > 0.5%, so the total number of rs numbers to use is 13,617,179. The human.protein.gpff file, which contains 36,229 genes and 732,776 variants, was compared to each of the 13,617,179 variants in the combined ESPdb and snp137common file. Some of these variants are not necessarily associated with genes. The human.protein.gpff file matched 80,598 variants with the variants in the snp137common/ESPdb file. There were 9,491 genes with no variants. There were 19,614 genes with a maximum of one variant variant in any peptide. There were 5,772 genes with one or more peptides with two variants. There were 996 genes with one or more peptides with three variants. This left 356 genes with one or more peptides with four variants or more per peptide. Even though there are 80,598 unique variants there are a total number of 127,099 variants in the database file, because of isoforms. Of these 127,099 variants, 1,518 are simple stop codons "*"; 47 more are not simple stops e.g., "y*w". Six variants are replaced by a '-', which is a deletion; these were not included in the variant database. 126,477 variants have a single amino acid replacement, and 622 have multiple amino acid insertions. The new variant database contains 53,476 sequences and the reference database contains 36,229 sequences. Of the 7,124 genes with two variants or more in a peptide(s), there were 1097 variants that share their variant position with only one other variant, 37 share with two other variants and three share their position with three other variants.

**Validation of Genetically Variant Peptides and Imputed nsSNP Alleles**

Genetically variant peptides were processed to reduce false-positive assignment using the following criteria for exclusion of a peptide from analysis: low-quality expectation scores (X!Tandem, log(e) < –2), nsSNPs occurring in less than 1% of the population, the presence of MS/MS fragmentation masses consistent with the major allele, and low variance between theoretical and observed primary masses (< 0.2 Da). Amino acid polymorphisms were excluded from the analysis due to lack of discrimination or likely chemical rather than genetic causes (www.unimod.org)[3-5]. Peptides that were potentially derived from paralogous sequences, or that were potentially expressed in more than one gene product, were removed from the analysis (S2 File)[6].

Validation of imputed DNA polymorphisms was carried out by first designing PCR primers that flanked the variant, using the Primer 3 program (S12 Table, Whitehead Institute for Biomedical Research). Each primer set was tested for specificity. PCR reactions were carried out using the AccuPrime™ *Taq* DNA Polymerase System (Invitrogen™) following the manufacturer’s specifications. PCR product was then treated with ExoSAP-IT® (Affymetrix) and subjected to Sanger Dideoxy Sequence analysis on an Applied Biosystems 3730xl 96-capillary DNA Analyzer by the DNA Sequencing Core Facility, University of Utah Health Science Cores. Polymorphisms in the DNA sequence were automatically called using CodonCode Corporation Aligner software and were subsequently viewed and individually verified by the user (Figure 1, S2 Table).

**Mitochondrial Haplotype Analysis**

The HV1 and HV2 segments of the D-Loop of mitochondrial DNA in each subject was sequenced, haplotyped, and sub-clade haplotype proportion determined from a database of the Utah population (S11 Table)[7].

**References**

1. Rice RH. Proteomic analysis of hair shaft and nail plate. Journal of cosmetic science. 2011;62(2):229-36. Epub 2011/06/04. PubMed PMID: 21635850; PubMed Central PMCID: PMC3227502.

2. Laatsch CN, Durbin-Johnson BP, Rocke DM, Mukwana S, Newland AB, Flagler MJ, et al. Human hair shaft proteomic profiling: individual differences, site specificity and cuticle analysis. PeerJ [Internet]. 2014 5 August 2014; 2. Available from: <http://dx.doi.org/10.7717/peerj.506>.

3. Jeong J, Jung Y, Na S, Jeong J, Lee E, Kim MS, et al. Novel oxidative modifications in redox-active cysteine residues. Molecular & cellular proteomics : MCP. 2011;10(3):M110 000513. Epub 2010/12/15. doi: 10.1074/mcp.M110.000513. PubMed PMID: 21148632; PubMed Central PMCID: PMC3047142.

4. Solazzo C, Wilson J, Dyer JM, Clerens S, Plowman JE, von Holstein I, et al. Modeling deamidation in sheep alpha-keratin peptides and application to archeological wool textiles. Analytical chemistry. 2014;86(1):567-75. doi: 10.1021/ac4026362. PubMed PMID: 24299235.

5. Ghesquiere B, Gevaert K. Proteomics methods to study methionine oxidation. Mass spectrometry reviews. 2014;33(2):147-56. doi: 10.1002/mas.21386. PubMed PMID: 24178673.

6. Musumeci L, Arthur JW, Cheung FS, Hoque A, Lippman S, Reichardt JK. Single nucleotide differences (SNDs) in the dbSNP database may lead to errors in genotyping and haplotyping studies. Human mutation. 2010;31(1):67-73. doi: 10.1002/humu.21137. PubMed PMID: 19877174; PubMed Central PMCID: PMC2797835.

7. Foundation TSMG. The Sorenson Molecular Genealogy Foundation Mitochondrial Database 2013. Available from: <http://www.smgf.org>.
