# Supplementary material for: Demonstration of Protein-Based Human Identification Using the Hair Shaft Proteome
Source: PLoS One. 2016 Sep 7;11(9):e0160653. doi: 10.1371/journal.pone.0160653 (PMC5014411; doi:10.1371/journal.pone.0160653)
Supplement: S3 Table — Individual genotypes for nsSNP loci (rs3894194, rs56030650) from the 1000 Genome Project (www.1000genomes.org, phase 1) were collated and genotype frequency (gf) of each combination calculated for both the European (EUR, n = 379) and African (AFR, n = 246) populations. Corresponding single amino acid polymorphisms are indicated in red. (PDF) [file pone.0160653.s014.pdf]

Gene Name	rs Number	Hg19 Coordinate of variant	Annealing temp	size	primer for seq	Forward Primer Sequence	Reverse Primer Sequence
KRT81	rs6580873	chr12:52681925	64	287	F	CTGCGGGTGACAATGTCG	GTGGGAGTGTTGGACAGGAT
KRT83	rs2852464	chr12:52710721	64	239	F	GTGTCCTGTGCCGCTCAC	GGGATTAGGAGAAGCCCAGA
KRT31	rs6503627	chr17:39553547	64	388	F	TTCTGCTGGAGCTCCTCAAT	TCTGTCTTCAGCCAGGCACT
LRRC15	rs13070515	chr3:194080916	61.4	274	F	GGGAGATGAAGCTGATCTGA	AACAACCACAACCTCCAGAGA
BLMH	rs1050565	chr17:28576076	58.3	250	F	TTCAGTCCCTGGATCTGTCC	TCATAAGTCTTCTGTTCTTTTCTCCA
HEXB	rs10805890	chr5:73992881	54	220	F	TCATTGAGTTCAAGACAACAGAAA	GGAGACATCTTCAGAATAGACAACA
KRT33A	rs12937519	chr17:39503163	64	336	F	GGTGGGGAAGATTGAGAGTG	GAGGCAGGGTCTGATCCTTT
GSTP1	rs1695	chr11:67352689	54	230	F	TCCCCAGTGACTGTGTGTTG	GCACAAGAAGCCCCTTTCT
SERPINB5	rs1455555	chr18:61170782	54	222	F	CCAATGCCAAGGTCAAACTC	CTCCTGGCACCTCTATGGAA
GSDMA	rs3894194	chr17:38121993	58.3	241	F	TCAGAGCCGGGTAAACTCTC	CTTCCTCTTCCTCAGCACCA
KRT34	rs2239710	chr17:39535859	54.8	244	R	TGAGTGGCCATGTGCTTAGA	CAGGAAAATGGCAGATCCA
JUP	rs143043662	chr17:39913771	63.4	179	F	GCTTGAAGAGGGAGTTGGTG	AGGAGGCTGGGTCCCACT
KRT81	rs2071588	chr12:52685096	63.4	247	F	GACCGACACGGTGGTGAT	GACCTGCGGATCAGGATTT
KRT31	rs112544857	chr17:39551763	58	323	F	AAGGGGAGAAAGGATCAGAC	CAGGGTTTCAACAAGCAATG
KRT1	rs17678945	chr12:53070174	58	226	F	GTCCCTTCCTCACCTGCTT	CTTGCAGCAGTCCATCAGTG
LRRC15	rs13060627	chr3:194080983	54.8	147	R	CCCAAAGAGAGTAAGACGGTTG	CAGAACCAGATTGGACTGCTC
GSDMA	rs56030650	chr17:38131187	54.8	267	F	ACATGAGGCCTCGAAAAAGA	TGAAGACCTTATTCCCCACTT
KRT32	rs2071563	chr17:39619115	54	295	F	TTCCTATAACCTCCCAGAATCC	TCCTTTGCCTTAGAGGGACT
KRT32	rs2071561	chr17:39622068	54.8	243	F	GTTCCTCCCCCTCTGCTTC	TTCACCCTTGGTTGCTGAC
DSP	rs6929069	chr6:7581636	64	349	F	TGGCCACCTGAGGGAAAAGC	CTGCAGCTGGCTCCTTAGTT
GSDMA	rs7212938	chr17:38122680	64	375	F	CTGCAGACCCAACAGACACT	GGAGGTGTGACTAGGAAATGG
JUP	rs41283425	chr17:39925713	62.1	387	F	GCCCCAGTCAGACTCATCAC	GAGTACCAGATGTCCACAACAGC
KRT35	rs743686	chr17:39637244	62.5	395	F	TCGTTCAGGGATTGCATGGT	CTGGGGTAAGGAGCTGCTTC
KRT40	rs150812789	chr17:39135207	62.1	408	F	CATCAGCCTCTTTGTGACTC	AACTGAGACTCACAGAAGC
S100A3	rs36022742	chr1:153520954	62	403	F	CCCCAGGTCTCTCTTTGCAG	AATTAGGAGGGGCTGGTGTG
TGM3	rs214803	chr20:2290333	64	310	F	GAATTTGGCTCTGCCCTTGAC	GGGAATGAGCAGGTACCTGTG
KRT85	rs112554450	chr12:52758810	64	383	F	CCACTGGATATCTGCTCTGGG	AGGCTCCCTCTGACTTCTTC
KRT32	rs11078993	chr17:39619193	60.2	273	R	GTCCAGCAGCACCTGGTAC	GGTGTAGGGAGGGCTCATTT
KRT35	rs2071601 & rs12451652	chr17:39633349 & chr17:39633354	54	388	F	TGGGTAGAGGCCAAGTTCAA	CATCACGTGACTCCAGCAAG
KRT85	rs61630004	chr12:52760957	Betaine20%TD	321	F	TCCTTCTCCTCCTGCTTCAC	CAGCTCCTGCTCAGCTGTG
KRT37	rs9910204	chr17:39580739	Betaine20%TD	367	F	CCATTCAGGGTGTTTTTGC	AGTCTCAGTGGCTCAGCCTTC
KRT32	rs72830046	chr17:39620565	54	330	R	GGCCCACCTGCATATTGAAC	TTGAAGAGCACTGGGATGCT

S3 Table
