# Supplementary material for: Demonstration of Protein-Based Human Identification Using the Hair Shaft Proteome
Source: PLoS One. 2016 Sep 7;11(9):e0160653. doi: 10.1371/journal.pone.0160653 (PMC5014411; doi:10.1371/journal.pone.0160653)
Supplement: S12 Table — Mitochondrial DNA in buffy coat DNA was isolated from a subset of European-American subjects (EA2) and HV1 and HV2 regions sequenced (S1 Methods). Mitochondrial DNA haplotypes and subclades were classified and percentage and population proportion determined relative to the Utah Population Database (Pr(mtDNA haplotype|Utah population)). Calculation of imputed nsSNP profile probabilities (Pr(imputed nsSNP-profile|EUR population)) were calculated relative to the European population as described in the Supplemental Methods (S1 Methods). The effect of binomial distribution on posterior allelic probabilities was determined and upper and lower limits (90% confidence interval) determined using parametric bootstrapping. Full Hardy-Weinberg equilibrium between gene boundaries, and full linkage-disequilibrium within them, were assumed. When independence between mitochondrial DNA haplotype and the imputed nsSNP allele profiles was assumed, the combined probability was calculated as the product of the two values. (PDF) [file pone.0160653.s023.pdf]

**LRRC15**		**EUR**		**AFR**	
**rs13070515**	**rs13060627**	**sum**	***gf***	**sum**	***gf***
G|G	C|C	210	0.554	154	0.626
G|G	(C|T, T|C)	19	0.050	51	0.207
G|G	T|T	0	0.000	3	0.012
(G|A, A|G)	C|C	1	0.003	0	0.000
(G|A, A|G)	(C|T, T|C)	125	0.330	30	0.122
(G|A, A|G)	T|T	3	0.008	4	0.016
A|A	C|C	0	0.000	0	0.000
A|A	(C|T, T|C)	0	0.000	0	0.000
A|A	T|T	21	0.055	4	0.016
G=ELSPGIFGPMPNLR<br>A=ELSlGIFGPMPNLR<br>**P286L**	C=LYLSNNHISQLPPSVFMQLPQLNR<br>T=LYLSNNHISQLPPSiFMQLPQLNR<br>**V264I**	**379**	**1.000**	**246**	**1.000**

S12 Table
